# Systematic meta-review of supported self-management for asthma: a healthcare perspective

**DOI:** 10.1186/s12916-017-0823-7

**Published:** 2017-03-17

**Authors:** Hilary Pinnock, Hannah L. Parke, Maria Panagioti, Luke Daines, Gemma Pearce, Eleni Epiphaniou, Peter Bower, Aziz Sheikh, Chris J. Griffiths, Stephanie J. C. Taylor, for the PRISMS and RECURSIVE groups, Stephanie J. C. Taylor, Hilary Pinnock, Chris J. Griffiths, Trisha Greenhalgh, Aziz Sheikh, Eleni Epiphaniou, Gemma Pearce, Hannah L. Parke, Anna Schwappach, Neetha Purushotham, Sadhana Jacob, Peter Bower, Maria Panagioti, Gerry Richardson, Elizabeth Murray, Anne Rogers, Anne Kennedy, Stanton Newman, Nicola Small

**Affiliations:** 1 Asthma UK Centre for Applied Research, Allergy and Respiratory Research Group, Usher Institute of Population Health Sciences and Informatics, University of Edinburgh, Doorway 3, Medical School, Teviot Place, Edinburgh, EH8 9AG UK; 2 Centre for Primary Care and Public Health, Barts and The London School of Medicine and Dentistry, Queen Mary University of London, London, UK; 3 NIHR School for Primary Care Research, Centre for Primary Care, Manchester Academic Health Science Centre, University of Manchester, Manchester, UK; 4 Centre for Technology Enabled Health Research (CTEHR), Coventry University, Coventry, UK

**Keywords:** Supported self-management, Asthma, Systematic meta-review, Health economic analysis, Meta-analysis

## Abstract

**Background:**

Supported self-management has been recommended by asthma guidelines for three decades; improving current suboptimal implementation will require commitment from professionals, patients and healthcare organisations. The Practical Systematic Review of Self-Management Support (PRISMS) meta-review and Reducing Care Utilisation through Self-management Interventions (RECURSIVE) health economic review were commissioned to provide a systematic overview of supported self-management to inform implementation. We sought to investigate if supported asthma self-management reduces use of healthcare resources and improves asthma control; for which target groups it works; and which components and contextual factors contribute to effectiveness. Finally, we investigated the costs to healthcare services of providing supported self-management.

**Methods:**

We undertook a meta-review (systematic overview) of systematic reviews updated with randomised controlled trials (RCTs) published since the review search dates, and health economic meta-analysis of RCTs. Twelve electronic databases were searched in 2012 (updated in 2015; pre-publication update January 2017) for systematic reviews reporting RCTs (and update RCTs) evaluating supported asthma self-management. We assessed the quality of included studies and undertook a meta-analysis and narrative synthesis.

**Results:**

A total of 27 systematic reviews (*n* = 244 RCTs) and 13 update RCTs revealed that supported self-management can reduce hospitalisations, accident and emergency attendances and unscheduled consultations, and improve markers of control and quality of life for people with asthma across a range of cultural, demographic and healthcare settings. Core components are patient education, provision of an action plan and regular professional review. Self-management is most effective when delivered in the context of proactive long-term condition management. The total cost (*n* = 24 RCTs) of providing self-management support is offset by a reduction in hospitalisations and accident and emergency visits (standard mean difference 0.13, 95% confidence interval −0.09 to 0.34).

**Conclusions:**

Evidence from a total of 270 RCTs confirms that supported self-management for asthma can reduce unscheduled care and improve asthma control, can be delivered effectively for diverse demographic and cultural groups, is applicable in a broad range of clinical settings, and does not significantly increase total healthcare costs. Informed by this comprehensive synthesis of the literature, clinicians, patient-interest groups, policy-makers and providers of healthcare services should prioritise provision of supported self-management for people with asthma as a core component of routine care.

**Systematic review registration:**

RECURSIVE: PROSPERO CRD42012002694; PRISMS: PROSPERO does not register meta-reviews

**Electronic supplementary material:**

The online version of this article (doi:10.1186/s12916-017-0823-7) contains supplementary material, which is available to authorized users.

## Background

Asthma is common, affecting 334 million people worldwide, and is responsible for substantial morbidity and an increasing burden on healthcare services globally [1]. In the UK, there are over 6 million primary care consultations, and 100,000 hospital admissions each year, at an estimated cost of £1 billion per year [2].

For a quarter of a century [3], national and international guidelines have recommended – unequivocally – that people with asthma should be provided with self-management education reinforced by a personalised asthma action plan and supported by regular review [4, 5], though mode of delivery, personnel delivering the support, the targeted group and the intensity of the intervention vary [6]. The 2014 UK National Review of Asthma Deaths provided a stark reminder of the importance of ensuring that people with asthma respond in a timely and appropriate manner to deteriorating symptoms: only 23% had documented evidence of having been provided with self-management education and 45% of people who died had not sought or received medical attention in their final attack [7].

However, despite self-management being highlighted as a core component of all models of care for people with long-term conditions (LTCs) [8–10] and the concept being well established in the context of asthma [4, 5], in practice only a minority of people with asthma have an action plan [11]. Effective implementation requires a whole systems approach, combining active engagement of patients with the training and motivation of professionals embedded within an organisation in which self-management is valued [12]. Patient organisations, healthcare professionals, policy-makers, commissioners and providers of healthcare services thus need an up-to-date systematic overview of the evidence to inform decisions about prioritisation of supported self-management and to underpin implementation strategies within diverse healthcare systems.

The data presented in this paper are derived from two parallel programmes of work on supported self-management in LTCs commissioned by the National Institute of Health Research: Practical Systematic Review of Self-Management Support (PRISMS) [13] and Reducing Care Utilisation through Self-management Interventions (RECURSIVE) [14]. In the context of asthma, we aimed to answer questions of importance to clinicians, patient-interest groups, managers responsible for developing healthcare services and policy-makers: can supported self-management reduce the use of healthcare resources and improve asthma control? More specifically, in which target groups has it been shown to work, which components are important, in what healthcare contexts, and at what cost?

## Methods

We used established methodology for undertaking a meta-review of systematic reviews (PRISMS) and a systematic review of randomised controlled trials (RCTs) (RECURSIVE) [15]. The PRISMS and RECURSIVE reviews were undertaken during 2012–2013 with initial searches completed in November 2012 and May 2012, respectively. We updated the PRISMS searches in March 2015 with a pre-publication update in January 2017, and the RECURSIVE searches in September 2015. RECURSIVE is registered on PROSPERO: CRD42012002694. (PRISMS could not be registered because PROSPERO does not register meta-reviews.)

### Search strategy

Table 1 summarises the PICOS criteria, search strategies, sources and search dates; further details are in Additional file 1. The PRISMS search strategy involved searching nine electronic databases using the terms: ‘self-management support’ AND ‘asthma’ AND ‘systematic review’. We defined self-management as ‘the tasks that individuals must undertake to live with one or more chronic conditions. These tasks include having the confidence to deal with medical management, role management and emotional management of their conditions’ [16]. For the update, we searched not only for systematic reviews published after our initial search date but also for RCTs published after the search dates used by the included systematic reviews (see Additional file 2 for the details of these dates). Included systematic reviews were grouped according to the populations studied (children, adults or ethnic minority groups) and the search dates of the reviews extracted. Dates for the update RCT search were set from the date of the latest review search within each population group.

**Table 1:** PICOS search strategy and sources for the reviews

	PRISMS systematic meta-review	RECURSIVE systematic review
Population	Adults/children with asthma, from all social and demographic settings. Multi-condition studies if asthma data reported.	Adults (≥18 years) with asthma (within a wider search of long-term conditions), excluding studies in the developing world.
Intervention	Self-management support interventions.	Self-management support interventions.
Comparator	Typically ‘usual care’ or less intense self-management interventions.	Typically ‘usual care’ or less intense self-management interventions.
Outcomes	Unscheduled use of healthcare services (admissions, A&E attendances, unscheduled consultations), health outcomes (asthma control), quality of life, process outcomes (ownership of action plans, self-efficacy).	Healthcare utilisation with comprehensive measures of costs or major cost drivers (i.e. hospitalisation, A&E attendances), quality of life.
Settings	Any healthcare setting.	Any healthcare setting.
Study design	Systematic reviews of RCTs. RCTs published after the date of the last search in the included systematic reviews (see Additional file 2).	RCTs
Dates	Initial database search: January 1993 (3 years before the publication of the earliest systematic review identified in scoping work) to July 2012. Manual and forward citations were completed in November 2012. Update search: March 2015. Pre-publication update January 2017.	Initial database search: inception to May 2012. Update search: September 2015.
Databases	MEDLINE, EMBASE, CINAHL, PsycINFO, AMED, BNI, Cochrane Database of Systematic Reviews, Database of Abstracts of Reviews of Effects, and ISI Proceedings (Web of Science).	CENTRAL, CINAHL, EconLit, EMBASE, Health Economics Evaluations Database, MEDLINE, MEDLINE In-Process & Other Non-Indexed Citations, NHS Economic Evaluation Database, and the PsycINFO.
Manual searching	Systematic Reviews, Health Education and Behaviour, Health Education Research, Journal of Behavioural Medicine, and Patient Education and Counseling.	Systematic Reviews.
Forward citations	On all included systematic reviews. Bibliographies of eligible reviews.	None.
In progress studies	Abstracts were used to identify recently published trials.	Abstracts were used to identify recently published trials.
Other exclusions	Previous versions of updated reviews. Papers not published in English.	Not applicable.

*A&E* accident and emergency, *RCT* randomised controlled trial

The RECURSIVE search strategy in nine databases comprised the terms: ‘self-management support’ AND ‘long-term condition’ AND ‘healthcare use’ AND ‘randomised controlled trial’. (RECURSIVE included asthma and other LTCs in a single search.) We also specifically sought health economic publications linked to included RCTs.

### Identification of relevant papers

Table 2 summarises the PRISMS and RECURSIVE processes. Following training (repeated cycles of duplicate screening of 100 titles, team discussion and clarification of exclusion rules), one reviewer (HLP or GP for PRISMS; LD for the update; MP for RECURSIVE) reviewed titles and abstracts and selected possibly relevant studies. A random sample of titles and abstracts (10% in PRISMS; 40% in RECURSIVE) was examined by a second reviewer (HP for PRISMS; PB or NS for RECURSIVE) working independently as a quality check. The agreement was 97% for the initial search and 99% for the update in PRISMS and 87% for the initial search and 88% for the update in RECURSIVE.

**Table 2:** PRISMS and RECURSIVE processes for selection of studies, quality assessment, data extraction, analysis and interpretation

	PRISMS systematic meta-review	RECURSIVE systematic review
Title and abstract screening	Initial training. One reviewer selected studies for full-text screening. Quality check: Random sample of 10% checked independently by second reviewer. Agreement: 97% for the initial search and 99% for the update. Uncertainties resolved by discussion.	Initial training. One reviewer selected studies for full-text screening. Quality check: Random sample of 40% checked independently by second reviewer. Agreement: 87% for the initial search and 88% for the update. Uncertainties resolved by discussion.
Full-text screening	Following training, one reviewer selected possibly relevant studies for inclusion. Quality check: Random sample of 10% checked independently by second reviewer. Agreement: 83%. Uncertainties resolved by discussion.	Following training, one reviewer selected possibly relevant studies for inclusion. Quality check: Random sample of 30% checked independently by second reviewer. Agreement: 85%. Uncertainties resolved by discussion.
Quality assessment	Duplicate quality assessment using: R-AMSTAR [17] for systematic reviews (‘high-quality’ defined as ≥31), combined with size of the review (‘large’ defined as ≥1000 participants) to give star rating (1* to 3*). Cochrane Risk of Bias tool for RCTs [15]. Disagreements resolved by discussion.	Duplicate quality assessment using: Drummond for economic evaluations [18, 19]. Allocation concealment for RCTs. Disagreements resolved by discussion.
Data extraction	Data extraction by one reviewer. Quality check: 100% checked for accuracy by a second reviewer. Disagreements resolved by discussion.	Data extraction by one reviewer. Quality check: Random sample of 40% extracted independently by second reviewer. Disagreements resolved by discussion.
Analysis	Reviews/RCTs categorised according to the question(s) that they answered: • Does supported self-management reduce healthcare utilisation and improve control? • For which target groups does it work? • Which components contribute to effectiveness? • In what healthcare contexts does supported self-management work? Meta-Forest plots for pooled statistics of the primary outcome (healthcare utilisation). Narrative synthesis within categories.	Meta-analysis: Standardised mean differences (random effects model) to examine the effects of self-management support interventions on hospitalisation rates, A&E attendances, quality of life and total costs. Permutation plots of the data from trials reporting both utilisation (hospitalisation rates, A&E attendances or total costs) and health outcomes (quality of life).
Interpretation	Monthly teleconferences to enable synergies between PRISMS and RECURSIVE. End-of-project stakeholder conference to discuss findings and implications for commissioning and providing services for people with LTCs.	

*A&E* accident and emergency, *LTC* long-term condition, *R-AMSTAR* Revised Assessment of Multiple Systematic Reviews, *RCT* randomised controlled trial

After a similar training process, the full texts of all potentially eligible studies were assessed against the eligibility criteria (see Additional file 3) by one reviewer (HLP for PRISMS; LD for update; MP for RECURSIVE). Second reviewers undertook a 10% check for PRISMS (HP) and a 30% check for RECURSIVE (PB or NS), achieving 83% and 85% agreement, respectively. Disagreements were because unclear papers were included by the reviewer pending discussion with a lead investigator. Uncertainties and disagreements were resolved by full team discussion.

### Assessment of methodological quality

We used the R-AMSTAR (Revised Assessment of Multiple Systematic Reviews [17]) quality appraisal tool to assess the methodological quality of the systematic reviews included in the PRISMS study. This reflects both the quality of the review process and the rigour with which the review assessed the quality of the studies it included. We used the Cochrane Risk of Bias tool to assess the quality of RCTs included in the updated search [15]. Quality assessment was undertaken by HLP or LD and independently by a second reviewer (HP) with disagreements resolved by discussion within the team (EE, GP, HLP, ST and HP).

To reflect both quality and size of the review, we developed a star weighting system based on (a) the R-AMSTAR score (≥31 was defined as ‘high-quality’) and (b) the number of participants (≥1000 participants was defined as ‘large’):

*** Large high-quality review
** Either small high-quality review or large low-quality review
* Small low-quality review

In the RECURSIVE study, quality assessment of formal economic evaluations was undertaken using the Drummond checklist [18, 19]; RCTs reporting healthcare utilisation were assessed by judging allocation concealment (the quality component most associated with treatment effect [20]) as adequate or inadequate according to the Cochrane Risk of Bias tool [15].

### Outcomes

The primary outcome in the PRISMS meta-review was unscheduled use of healthcare resources (specifically unscheduled consultations, accident and emergency (A&E) department attendances and hospital admissions). Other outcomes of interest were asthma control, asthma-related quality of life and process outcomes (specifically, ownership of action plans). Healthcare utilisation rates and costs were the primary focus of the RECURSIVE review, especially major cost drivers (i.e. hospitalisation rates and costs) and comprehensive summaries including multiple sources of cost. The results of formal cost-effectiveness, cost-utility and cost–benefit analyses were also of interest.

### Extraction of data

Data for the PRISMS review were extracted by HLP and LD (update) using a piloted data extraction form, and checked independently by HP for integrity and accuracy. Disagreements were resolved by team discussion. We extracted data on review rationale, the self-management intervention under review, review methodology, summary details of included RCTs (participant demographics, comparison groups, settings, service arrangements, components, duration/intensity of the intervention, follow-up arrangements) and the results of meta-analyses and narrative syntheses. We extracted the findings and conclusions as synthesised by the authors of the systematic reviews, specifically avoiding going back to the individual primary studies. The RCTs in the update review were extracted using similar headings.

A piloted data extraction sheet was devised for RECURSIVE that included descriptive data (characteristics of studies, populations and interventions) and quantitative data (for use in meta-analyses). All the descriptive data and approximately 40% of the quantitative data were double-extracted by two members of the research team working independently.

### Data analysis

Meta-analysis is inappropriate at the meta-review level owing to the overlap of included RCTs between reviews. However, for the primary outcome, where two or more systematic reviews (including the RECURSIVE meta-analyses) present pooled statistics, we displayed the results graphically by creating ‘meta-Forest plots’. We undertook narrative syntheses to answer our key questions: Does supported self-management reduce use of healthcare resources and improve asthma control? For which target groups does it work? Which components contribute to effectiveness? and In what contexts does supported self-management work? We categorised the reviews and RCTs included in the PRISMS meta-review according to the question(s) that they answered (see Tables 3 and 4: column 3) and synthesised the findings within these categories.

**Table 3:** Summary table of findings of PRISMS systematic reviews and their relevance to the meta-review questions

Reference and weighting*; RCTs, n; Participants, n; R-AMSTAR; Date range of included RCTs	Comparison	Relevance to meta-review questions:	Interventions included	Target group(s)	Synthesis	Main results
		What is the impact? Target groups? Which components? Context?				
Bailey 2009 [25]** 4 RCTs 617 participants R-AMSTAR 36 RCTs 2000–2008	Culturally orientated programmes vs. usual care or limited/generic education. FU (mode): 12 mo, range 4–12 mo	Impact Target: Ethnic groups	Education, action plans, triggers and avoidance, collaboration with healthcare services. Language-appropriate asthma educators.	Minority groups: Puerto Rican, African-American, Hispanic, Indian sub-continent. Adults and children.	Meta-analysis Narrative analysis	Reduced hospitalisation in children (RR 0.32, 95% CI 0.15–0.70; 1 RCT) but not reported in adults. Improved QoL in adults (WMD 0.25, 95% CI 0.09–0.41; 2 RCTs). 2 of 2 RCTs reported a reduction in A&E visits and hospitalisations: one reported no difference in ‘use of healthcare resources’; 2 of 3 reported improved QoL (adults).
Bernard-Bonnin 1995 [26]** 11 RCTs 1290 participants R-AMSTAR 27 RCTs 1981–1991	Interactive teaching on self-management vs. standard care.	Impact Target: Children	Interactive teaching (one-to-one or group) to support asthma self-management.	Children 1–18 y. Overall severity classified as ‘mild to moderate’.	Meta-analysis Narrative analysis	Reduced hospitalisation (ES 0.06 ± −0.08) and emergency visits (ES 0.14 ± 0.09); 5 RCTs. Children with high baseline numbers of hospitalisations and emergency visits had greatest subsequent reduction in morbidity.
Bhogal 2006 [23]** 4 RCTs 355 participants R-AMSTAR 41 RCTs 1990–2004	Symptom-based written PAAPs vs. peak flow-based PAAP. FU (mode): 3 mo, range 3–24 mo	Target: Children Components: PEF vs. symptom monitoring	Asthma education plus PAAPs for both parents and children. Generally contained 3 steps: often employing ‘traffic lights’. Monitoring varied: either daily or when symptomatic.	Children 6–19 y with mild to severe asthma.	Meta-analysis	Symptom-based PAAPs reduced unscheduled care compared to peak flow-based PAAPs (RR 0.73, 95% CI 0.55–0.99; 4 RCTs). No difference in hospital admissions (RR 1.51, 95% CI 0.35–6.65. Peak flow-based PAAPs reduced the number of symptomatic days/week (MD 0.45 days/week, 95% CI 0.04–0.26; 2 RCTs). No significant difference for adult or child QoL.
Zemek 2008 [24]** 5 RCTs 423 participants R-AMSTAR 41 RCTs 1990–2005	Written PAAPs vs. no PAAP. Symptom-based vs. PEF-based PAAP. FU (mode): 3 mo, range 0.5–24 mo	Impact: Target: Children Components: PAAP	Education for parents and children, plus PAAPs, with 3 steps: often employing ‘traffic lights’. Monitoring varied: either daily or when symptomatic.	School-aged children with mild to severe asthma.	Meta-analysis	A PEF-based PAAP reduced unscheduled care compared to no plan (WMD −0.50, 95% CI −0.83 to −0.17; 1 RCT). A PEF-based PAAP compared to no plan reduced symptom scores (WMD −11.80, 95% CI −18.22 to −5.38) and number of school days missed (WMD −1.03, 95%CI −1.85 to −0.21; 1 RCT).
Boyd 2009 [27]*** 38 RCTs 7843 participants R-AMSTAR 39 RCTs 1985–2007	Education targeting children/parents vs. low intensity education. FU (mode): 12 mo range 4–12 mo	Impact: Target: Children, A&E attendees	Education plus therapy review, self-monitoring, PAAPs, and trigger avoidance. Range of settings and professionals and mode of delivery.	Children 0–18 y who had attended A&E for asthma within the previous 12 mo.	Meta-analysis Subgroup analyses	Education reduced A&E attendances (RR 0.73, 95% CI 0.65–0.81; 17 RCTs), admissions (RR 0.79, 95% CI 0.69–0.92; 18 RCTs) and unscheduled consultations (RR 0.68, 95% CI 0.57–0.81; 7 RCTs). No effect on QoL (WMD 0.13, 95% CI 0.73–0.99; 2 RCTs). Subgroup analyses (type and timing of intervention, timing of outcome assessment or age of participants) did not change findings.
Bussey Smith 2009 [28]* 9 RCTs 957 participants R-AMSTAR 26 RCTs 1986 - 2005	Computerised education vs. traditional self-management FU (mode): 12 mo, range 3–12 mo	Impact: Components: Technology-based interventions	Interactive computerised educational asthma programmes (games tailored to the individual, web-based education, interactive communication devices).	Patients 3–75 y. 7 RCTs in children, 2 in adults; 4 RCTs in urban or inner-city populations.	Narrative analysis	1 of 4 improved hospitalisation, and 1 of 5 reduced unscheduled care. 5 of 9 studies found statistical improvements in asthma symptoms compared to control.
Chang 2010 [29]** 1 RCT 113 participants R-AMSTAR 40 RCT 2010	Education by IHWs vs. education no IHW. FU: 12 mo	Impact: Target: Ethnic groups	Initial clinical consultation, reinforced by home visits from a trained IHW. Personalised, child-friendly, culturally appropriate education materials.	African-American and Hispanic communities. Children 1–17 y; mean ~7 y.	Narrative analysis	There was no effect on hospitalisations (OR 1.58, 95% CI 0.37–6.79) or A&E attendances (OR 0.30, 95% CI −0.17 to 0.77; 1 RCT). Days absent from school were reduced by 21% in the intervention group (95% CI 5–36%; 1 RCT). Carer asthma QoL was not significantly different (MD 0.25, 95% CI −0.39 to 0.89).
Coffman 2009 [30]** 18 asthma RCTs 8077 participants R-AMSTAR 29 RCTs 1987-2007	School-based asthma education vs. usual care.	Impact: Target: Schoolchildren	School-based education on asthma, medication, monitoring, avoiding triggers. Delivered by nurses, health educators, peer counsellors, teachers, ± computer programmes.	Children 4–17 y. Severity: mild to severe, majority were Black or Latino.	Narrative analysis	Unscheduled healthcare was not reported. School absences significantly reduced in 5 of 13 RCTs. Days with symptoms were reduced in 3 of 8 RCTs. Nights with symptoms improved in 1 of 4 RCTs: 1 found improvement in the control group. QoL improved in 4 of 6 RCTs.
Gibson 2002 [31]*** 36 RCTs 6090 participants R-AMSTAR 39 RCTs 1986 –2001	Self-management programmes vs. usual care.	Impact: Components: Regular review Context: LTC care	Education (100%); self-monitoring of symptoms or PEF (92%); regular review by a medical practitioner (67%); PAAP (50%). Subgroup analyses based on these service models.	Adults and children. Range of settings, including hospital, emergency room, outpatients, community setting, general practice.	Meta-analysis Subgroup analysis	Self-management reduced hospitalisations (RR 0.64, 95% CI 0.50–0.82; 12 RCTs), A&E visits (RR 0.82, 95% CI 0.73–0.94; 13 RCTs] and unscheduled consultations (RR 0.68, 95% CI 0.56–0.81; 7 RCTs). Self-management reduced days off work/school (RR 0.79, 95% CI 0.67–0.93; 7 RCTs) and improved QoL (SMD 0.29, 95% CI 0.11–0.47; 6 RCTs). Optimal self-management (supported by a PAAP and regular review) reduced hospitalisations (RR 0.58, 95% CI 0.43–0.77; 9 RCTs), and A&E visits (RR 0.78, 95% CI 0.67–0.91; 9 RCTs).
Gibson 2004 [32]*** 26 RCTs 6090 participants R-AMSTAR 39 RCTs 1987–2002	Different components of written PAAPs vs. usual care.	Components: PAAPs	Complete PAAPs specified when/how to increase treatment (n = 17); incomplete omitted advice on increasing ICS (n = 4); non-specific (n = 5) only had general instructions.	Adults and children. Variety of settings, including hospital, emergency room, outpatients, community setting, general practice.	Action points % predicted vs. % best Treatment advice Non-specific plans	Benefits were found for any number of action points (2 to 4). Both % predicted and % best reduced hospitalisations, but only % personal best reduced A&E visits. PAAPs which included advice on increasing ICS and starting oral steroids reduced hospitalisations and A&E visits. Efficacy of incomplete and non-specific PAAPs was inconclusive.
Moullec 2012 [33]** 18 RCTs 3006 participants R-AMSTAR 27 RCTs 1990–2010	Interventions to improve inhaled steroid adherence vs. usual care. FU (mode): 12 mo, range 0.25–24 mo	Context: LTC care	All studies included self-management; some included components of CCM: decision support, delivery system design, clinical information systems.	Moderate to severe asthma (one RCT included COPD). Aged 35–50 y. Women over-represented.	Meta-analysis	Effect size for adherence to ICS compared by number of components of the CCM in the study: 1 CCM component (n = 13): small ES 0.29 (95% CI 0.16–0.42) 2 CCM components (n = 5): large ES 0.53 (95% CI 0.40–0.66) 3 CCM components (no studies) 4 CCM components (n = 4) very large ES 0.83 (95% CI 0.69–0.98).
Newman 2004 [34]** 18 asthma RCTs (of 63 RCTs) 2004 participants R-AMSTAR 23 RCTs 1997 –2002	Self-management interventions vs. standard care/basic information.	Impact:	Individual/group interventions, focused on symptom monitoring, trigger avoidance and adherence to medication. A few used techniques to address barriers to effective self-management.	Adults with 3 LTCs (including asthma).	Narrative analysis and comparison between interventions	7 of 11 studies reported a reduction in unscheduled healthcare. 6 of 12 studies reported improved QoL. 3 of 8 studies reported reductions in severity of symptoms, all used education and action plans. 8 of 14 reported improved adherence.
Postma 2009 [35]** 7 RCTs 2316 participants R-AMSTAR 23 RCTs 2004–2008	CHWs vs. usual care. FU (mode): 12 mo, range 4–24 mo	Impact: Target: Ethnic groups, children	CHWs from the same community as participants. Education on asthma, lifestyle and trigger avoidance, with resources to reduce allergen exposure.	Children 5–9 y with allergies and low-income. Mainly African-American and Hispanic.	Narrative review	3 of 6 studies reported reduced hospitalisation and reduced unscheduled consultations. 4 of 6 reported reduced A&E attendances ‘Consistent and significant decrease in caregiver-reported asthma symptoms among intervention subjects compared with control subjects in 6 studies.’
Powell 2009 [36]*** 15 RCTs 2460 participants R-AMSTAR 34 RCTs 1990–2001	Self-management vs. physician-reviewed management. Comparison of modified PAAPs.	Components: PAAP, regular review Context: LTC care	Self- vs. physician adjustment of medication (n = 6 studies). PEF vs. symptoms PAAPs (n = 6). Other variations (n = 3).	Adults with asthma recruited from a range of primary, community, A&E and secondary care.	Self- vs. physician management Symptoms vs. PEF-modified PAAPs	Of 6 studies: 4 reported no difference in hospitalisation, 1 reported no difference in A&E visits, 3 reported inconsistent effects on unscheduled consultations. Of 6 studies, 6 reported no difference in hospitalisation, 5 reported inconsistent effects on A&E visits. Omitting regular review (1 RCT) or reducing intensity of education (1 RCT) increased unscheduled consultations. Verbal (vs. written) PAAPs had no effect on hospitalisations or A&E visits (1 RCT).
Ring 2007 [37]*** 14 RCTs 4588 participants R-AMSTAR 35 RCTs 1993– 2005	Interventions encouraging use of PAAPs vs. usual care.	Context: Organisation of care	Interventions promoting PAAP ownership or use. Diverse interventions (educational, prompting, asthma clinics, asthma management systems, quality improvement).	Adults or children with moderate to severe asthma; some post-exacerbation.	Narrative analysis	4 of 5 studies of education, 1 of 2 studies of telephone consultations, 1 of 2 studies of asthma clinics and 1 of 2 studies of asthma management systems reported increased PAAP ownership. 1 study of self-management education, 1 of 2 studies of telephone consultations and 1 of 2 studies of asthma management systems increased understanding/use of PAAPs.
Tapp 2007 [38]*** 13 RCTs 2157 participants R-AMSTAR 39 RCTs 1979–2009	Asthma education at A&E visit vs. usual care. FU (mode): 6 mo, range 2–18 mo	Impact: Target: Post A&E attendance	Asthma education provided by asthma or A&E nurses within a week of A&E visit included PAAPs, triggers, monitoring, inhalers and medication.	Adults recruited during A&E attendance.	Meta-analysis Narrative analysis	The intervention reduced hospital admissions (RR 0.50, 95% CI 0.27–0.91; 5 RCTs), A&E visits (RR 0.66, 95% CI 0.41–1.07; 8 RCTs). Effect on QoL (2 RCTs) was inconsistent. There was no effect on days off work/school.
Toelle 2004 [39]** 7 RCTs 967 participants R-AMSTAR 38 RCTs 1990– 2001	Written PAAP vs. no plan. Symptom vs. PEF-based PAAP. FU (mode): 12 mo, range 6–12 mo	Components: PAAP	Peak flow-based written PAAP or symptom-based written PAAP delivered in primary or tertiary care.	Adults 28–45 y and children in 1 RCT.	Meta-analysis Subgroup analysis	Unscheduled healthcare: assessed in 1 RCT, not reported by systematic review. No difference between symptom and peak flow-based PAAPs in hospitalisations (RR 1.17, 95% CI 0.31–4.43; 3 RCTs) or A&E attendances (RR 1.17, 95% CI 0.31–4.43; 3 RCTs). Symptom-based PAAPs were more effective at reducing unscheduled consultations (RR 1.34, 95% CI 1.01–1.77; 2 RCTs).
Welsh 2011 [40]*** 12 RCTs 2342 participants R-AMSTAR 41 RCTs 1986–2010	Home-based self-management vs. routine care or general education. FU (mode): 12 mo, range 6–24 mo	Impact: Target: Children	Language-appropriate education (asthma, triggers, medication, inhalers, self-management with PAAPs). Also homework, technology devices, 24-hour hotline.	Children (mostly <12 y) recruited from recent healthcare visit. Mainly ethnic and/or deprived communities in USA.	Meta-analysis Narrative analysis	No difference between groups in mean number of A&E visits (MD 0.04, 95% CI −0.20 to 0.27; 2 RCTs). 2 of 5 studies reported hospitalisation: one found a reduction and one an increase in the intervention group. Effect on A&E visits (6 RCTs) was inconsistent. Overall no effect on QoL was found in 5 studies.
Bravata 2009 [41]*** 63 RCTs 13,476 participants R-AMSTAR 40 RCTs 1966–2006	Self-management QI vs. other QI strategies.	Impact: Target: Children	Self-monitoring or self-management. Patient/caregiver education. Provider education. Organisational change and interventions with multiple QI strategies.	Children <18 y.	Meta-analysis	Interventions targeting parents/caregivers reduced hospitalisation rates by 1.2% per year (95% CI 0.1–2.4; n = 5). Self-management intervention studies improved symptom-free days by 2.8% (95% CI 0.6–5.0), which equalled 0.8 days per month (n = 7); and reduced monthly school absenteeism by 0.4% (95% CI 0–0.7), which equalled 0.1 day per month (n = 16). Longer duration of intervention increased the effect on school absences.
Denford 2014 [43]*** 38 RCTs 7883 participants R-AMSTAR 36 RCTs 1993–2000	Asthma self-care vs. usual/less intensive intervention. FU (mode): 12 mo, range 3–18 mo	Impact: Components: Behaviour change	Commonest behavioural change techniques including: self-monitoring (n = 30), instruction (n = 27), goal-setting (n = 26) and inhaler technique (n = 24).	Adults ≥18 y with a diagnosis of asthma.	Meta-analysis	Intervention group participants had reduced asthma symptoms (SMD −0.38, 95% CI −0.52 to 0.24; 27 RCTs) and unscheduled healthcare use (OR 0.71, 95% CI 0.56–0.9; 23 RCTs). Increased adherence to preventative medication compared to control (OR 2.55, 95% CI 2.11–3.10; 16 RCTs).
de Jongh 2012 [42]** 1 asthma RCT (of 4) 16 participants R-AMSTAR 35 RCTs 1993–2009	Mobile phone messaging for self- management vs. usual care. FU: range 4–12 mo	Components: Mobile phone messaging	Self-management interventions delivered by mobile phone messaging.	Participants of all ages, gender or ethnicity. Included any LTC (one asthma study).	Narrative synthesis	In the single asthma study, there were fewer admissions (2 vs. 7) but more unscheduled consultations (21 vs. 15) in the intervention group compared to the usual care group. The pooled asthma symptom score showed a significant difference between groups, favouring the intervention group (MD −0.36, 95% CI −0.56 to −0.17).
Kirk 2012 [44]** 10 asthma RCTs 2195 participants R-AMSTAR 23 RCTs 1995–2010	Self-care support vs. usual care. FU (mode): 12 mo, range 3–24 mo	Impact: Target: Children	Interventions aiming to help children take control of and manage their condition, promote their capacity for self-care and/or improve their health.	Children ≤18 y with a LTC: asthma (10 RCTs), cystic fibrosis (2) or diabetes (1).	Narrative synthesis	Of 8 RCTs, 2 reported fewer asthma admissions, 5 reported fewer A&E attendances and 2 of 3 reported fewer unscheduled consultations. Control improved in 5 of 8 RCTs. Qol improved in 2 of 5 RCTs.
Marcano Belisario 2013 [45]** 2 RCTs 408 participants R-AMSTAR 39 RCTs 2000–2013	Self-management apps vs. traditional self-management. FU: 6 mo	Components: Smartphone Apps	Self-management support interventions provided by smartphone app.	Adults with clinician-diagnosed asthma.	Narrative synthesis	Of 2 RCTs, 2 reported no difference in hospital admissions; 1 reported fewer A&E attendances compared to control; 1 found no difference in unscheduled GP consultations or out of hours consultations, but reduced primary care nurse consultations; 1 reported no difference in MD in Asthma Control Questionnaire scores between the intervention and control group at 6 months; 1 found improved QoL in the intervention group.
Press 2012 [46]*** 5 RCTs (of 15 studies) 1459 participants R-AMSTAR 34 RCTs 1950–2010	Interventions targeted at ethnic minority groups vs. usual care. FU (mode): 6 mo, range 0.25–32 mo	Impact: Target: Ethnic groups	Interventions targeting ethnic populations in US. 15 were education-based, 9 were system-level interventions, 5 were culturally tailored and community-based, 10 were hospital-based.	Adults ≥18 y. Ethnic minority groups: African-Americans (10 studies, Latinos (4 studies).	Narrative synthesis	An education intervention reduced A&E attendance in 2 of 4 RCTs and hospital admissions in 2 of 3 RCTs. Symptoms were not reduced in any of the 3 RCTs that measured control. QoL was improved in 3 of 4 RCTs that used an asthma-related QoL outcome.
Stinson 2009 [47]* 4 asthma RCTs (of 9 studies) 826 asthma participants R-AMSTAR 28 RCTs 1993–2008	Internet-based self-management vs. usual care. FU (mode): 12 mo, range 3–12 mo	Target: Children Components: Internet-based	Any Internet-based or enabled self-management intervention.	Children 6–12 y or adolescents 13–18 y with LTCs: asthma (4 RCTs), pain (1), encopresis (1), brain injury (1) or obesity (1).	Narrative synthesis	1 RCT reported no difference in hospitalisations compared to control, 1 RCT reported significant reductions in A&E visits and 1 of 2 RCTs showed fewer unscheduled consultations. 4 out of 4 reported significant improvement in a measure of control. 1 of 4 asthma RCTs reported a significant benefit on QoL.

*Abbreviations*: *A&E* accident and emergency, *CCM* chronic care model, *CHW* community health workers, *CI* confidence interval, *COPD* chronic obstructive pulmonary disease, *ES* effect size, *FU* follow-up, *ICS* inhaled corticosteroid, *IHW* indigenous healthcare workers, *LTC* long-term condition, *MD* mean difference, *mo* months, *OR* odds ratio, *PAAP* personalised asthma action plan, *PEF* peak expiratory flow, *QI* quality improvement, *QoL* quality of life, *RR* risk ratio, *SMD* standardised mean difference, *WMD* weighted mean difference, *y* years

**Table 4:** Summary table of findings of update randomised controlled trials and their relevance to the meta-review questions

Reference and weighting; Participants, n; Risk of bias	Comparison	Relevance to meta-review questions:	Study type and interventions included	Target group(s)	Main results [1^o^] is the defined primary outcome
		What is the impact? Target groups? Which components? Context?			
Al-Sheyab 2012 [48] n = 261 HIGH risk of bias	Adolescent Asthma Action programme vs. standard care. FU: 3 mo	Target: Adolescents Components: Peer education	Cluster RCT. Triple A. Peer leaders from year 11 were trained to deliver programme to years 8, 9 and 10.	Adolescents in Jordanian high school. I group had fewer females, fewer symptoms and higher English proficiency.	Compared to control improvements QoL score improved [I: 5.42 (SD 0.14) vs C: 4.07 (SD 0.14) MD 1.35 (95%CI 1.04–1.76)].
Baptist 2013 [49] n = 70 HIGH risk of bias	Personalised asthma self-regulation intervention vs. education session. FU 12 mo	Target: Older adults Components: Health educator	RCT. 6-session programme (group telephone). Patients selected an asthma-specific goal, and addressed potential barriers. Control is single session basic education + 2 telephone calls.	Aged ≥65 y. Physician diagnosis of asthma, no restriction in severity. Majority Caucasian.	No between-group differences in A&E visits or hospitalisations. Healthcare utilisation was lower at 6 mo but not 12 mo. ACQ was similar at 1 mo and 6 mo. At 12 mo, I participants were 4.2 times more likely to have an ACQ score <0.75. [1^o^] QoL (mAQLQ) was significantly higher in the I than in C at all time points (1, 6 and 12 mo).
Ducharme 2011 [50] n = 219 LOW risk of bias	‘Take-home plan’ post A&E visit with PAAP + prescription information vs. prescription but no PAAP/information. FU: 28 days	Target: Children, A&E attendees Components: PAAP with prescription	RCT. Intervention is written PAAP with a ‘formatted’ prescription for ICS (i.e. including information about use) issued by A&E doctor on discharge following asthma exacerbation.	Canadian children 1–17 y recruited during A&E attendance for acute asthma (78% were under the age of 6 y).	No between-group differences in unscheduled care at 28 days. Compared to control, at 28 days children given the PAAP had better asthma control (proportion with Asthma Quiz Score <2 I: 58% vs. C: 41%; RR 1.36, 95% CI 1.04–1.86). No between-group differences in child/caregiver QoL at 28 days. [1^o^] Adherence to ICS declined from 90% (day 1) to 50% at day 14, with no significant group difference.
Goeman 2013 [51] n = 114 Low risk of bias	Person-centred education vs. written information. FU: 12 mo	Target: Older adults Components: Personalised education	RCT. Personally tailored education session with asthma educator based on responses to a questionnaire; inhaler technique.	≥55 y, community-based asthmatics with no restriction in asthma severity.	[1^o^] At 12 mo I participants had better asthma control than C (ACQ MD 0.3, 95% CI 0.06–0.5, *p* = 0.01) and better asthma-related QoL (*p* = 0.01). No significant difference in number of steroid courses (*p* = 0.17). At 12 mo, more I participants (n = 36, 61%) owned a PAAP compared to C (n = 21, 38%; *p* = 0.015). [1^o^] Similar adherence to ICS at 12 mo (*p* = 0.015).
Halterman 2014 [52] n = 638 LOW risk of bias	Personalised prompts for clinicians and parents, practice training and feedback vs. written guidelines. FU: 6 mo	Target: Children, deprived communities Components: Feedback Context: Community-based, clinical training	Cluster RCT. Intervention practices received personalised clinician and parent prompts + blank PAAP; practice training; feedback. Control practices sent guidelines.	Urban, primary care practices in deprived communities. Parents/children 2–12 y with persistent, poorly controlled asthma. Recruited from waiting room over 4 ystudy.	11% in both groups had an A&E visit or hospitalisation. [1^o^] Compared to control practices, at 2 mo children in the PAIR-UP practices had more symptom-free days [I: 10.2 days/2 weeks (SD 4.8) vs. C: 9.5 days/2 weeks (SD 5.1); MD 0.78, 95% CI 0.29–1.27] but the difference was not significant at 6 mo. Nights with symptoms remained significant at 6 mo [I: 1.4 (SD 3.0) vs. C: 1.8 (SD 3.2); MD −0.43; 95% CI −0.77 to −0.09].
Horner 2014 [53] n = 183 UNCLEAR risk of bias	Asthma plan for kids vs. teaching on general health and well-being. FU: 12 mo	Target: Children, rural communities	Cluster RCT. Programme delivered in 16 × 15 min sessions, 3 days/week for 5.5 weeks, by school nurses during lunch break + home visit.	Grades 2–5 (ages 7–11 y) with physician diagnosis of asthma.	No between-group difference for admissions or A&E visits. No between-group difference in QoL scores. Inhaler skill improved in the intervention group compared to control after 4 mo, with reported higher self-efficacy.
Joseph 2013 [54] n = 422 UNCLEAR risk of bias	Web-based asthma management intervention vs. control. FU: 12 mo	Target: Adolescents, urban deprived, ethnic groups Components: Web-based, behavioural change	RCT. Internet-based programme targeted at African-Americans/urban adolescents with traits (low motivation; low perceived emotional support; resistance to change; rebelliousness).	Grades 9–12 (ages 14–18 y) with physician diagnosis of asthma and report >4 days of restricted activity in the past 30 days at baseline.	No difference in reported A&E visits/hospitalisations at 12 mo. [1^o^] Compared to C, at 12 mo the I participants had fewer symptom-days (RR 0.8, 95% CI 0.6–1.0). No difference in nights with symptoms, schooldays missed, days of restricted activity or days had to change plans. Students characterised with rebelliousness or low perceived emotional support reported fewer symptom-days.
Khan 2014 [55] n = 91 HIGH risk of bias	Asthma education + individualised written PAAP vs. asthma education (excluding PAAP).	Target: Ethnic groups Components: Written PAAP	RCT. Both groups received individual asthma education during an OPD visit from a paediatrician + monthly FU. Intervention group trained in using a PAAP.	1–14 y. Recruited via A&E OPD with partly controlled asthma (daytime or nocturnal symptoms, activity limitation, lung function < 0% best or exacerbation in previous year).	[1^o^] Trend for improved outcomes at 6 mo but no significant between-group difference in proportion of children attending A&E (I: 36% vs. C: 52%; *p* = 0.141). There was no between-group difference in unscheduled doctor visits, asthma attacks, missed school days or night-time awakenings.
Rhee 2011 [56] n = 112 UNCLEAR risk of bias	Peer-led asthma education provided by peers at a day camp vs. adult-led camp.	Target: Adolescents. Components: Peer leaders	RCT. Asthma self-management skills + psychosocial skills taught at a day camp by peer leaders + monthly peer telephone contact. Control: Similar education delivered by adults. No telephone.	13–17 y (including low-income families). Mild/moderate/severe asthma. Asthma diagnosis for 1 y. Able to understand spoken and written English.	[1^o^] Both groups reported significantly increased QoL over time (F = 4.31, *p* = 0.002), with I group having significantly higher QoL at 6 mo (MD 11.38, 95% CI 0.96–21.79, *p* = 0.03) and 9 mo (MD 12.97, 95% CI 3.46–22.48, *p* = 0.008). Both groups reported improved attitude to asthma (F = 11.94, *p* = 0.001), with greater improvement in I at 6 mo (MD 4.11, 95% CI 0.65–7.56, *p* = 0.02).
Rikkers-Mutsaerts 2012 [57] n = 90 UNCLEAR risk of bias	Internet-based self –management vs. usual care. FU: 12 mo	Target: Adolescents. Components: Internet-based	RCT. Internet-based self-monitoring with algorithm-based advice. Programme included education (web-based + group), self-monitoring (FEV_1_ + ACQ), PAAP and 3–6 mo review.	12–18 y with mild to severe persistent asthma on regular ICS medication and poorly controlled at recruitment.	No between-group differences in exacerbations, physicians’ visits or telephone contacts. [1^o^] QoL was better in I group at 3 mo (PAQLQ I: 6.00 vs. C: 5.68; MD 0.40, 95% CI 0.17–0.62) but not at 12 mo (I: 5.93 vs. C: 6.05; MD 0.05, 95% CI 0.50–0.41). Asthma control was improved in I group at 3 mo (ACQ I: 0.96 vs. C: 1.19; MD −0.32, 95% CI −0.56 to −0.08) but not at 12 mo (I: 0.83 vs. C: 0.79; MD −0.05, 95% CI −0.35 to 0.25).
Shah 2011 [58] 150 GPs and 201 children LOW risk of bias	GP training (PACE study) vs. no training. FU: 12 mo	Targets: Children Components: GP training	Cluster RCT. GPs participated in 2 × 3-h workshops on communication and education strategies to facilitate quality asthma care.	150 GPs and 221 children with asthma in their care.	No between-group difference in hospitalisation/A&E visits (I: 18% vs. C: 12%; difference 6%, 95% CI −4 to 15). No between-group differences in school absence or parent absenteeism for child’s asthma. [1^o^] More patients in I group GPs had a PAAP (I: 61% vs. C: 46%; difference 15%, 95% CI 2–28).
van Gaalen 2013 [59] n = 107 HIGH risk of bias	Internet-based self –management vs. control (FU of SMASHING trial). FU: 30 mo	Target: Adults Components: Internet-based	RCT (FU study). Education + PAAP, self-monitoring and regular review. The 200 patients in original 12-mo trial were invited for FU after 18 mo.	Adults with asthma aged 18–50 y, using ICS. 107/200 (54%) participated: I group: 47/101 (47%); C group: 60/99 (61%). Participants ACQ was similar, but AQLQ was greater than in non-participants.	At 30 mo after baseline, there was a slightly attenuated improvement for both QoL (AQLQ adjusted between-group MD 0.29, 95% CI 0.01–0.57) and ACQ (adjusted MD of −0.33, 95% CI −0.61 to −0.05) scores in favour of the intervention. No between-group differences in FEV_1_.
Wong 2012 [60] n = 80 HIGH risk of bias	Symptom-based written PAAP vs. verbal counselling. FU: 6 mo	Target: Children, ethnic groups Components: Written PAAP	Single blinded RCT. Intervention was symptom-based PAAP given out at initial contact. Outcomes measured at baseline, 3, 6 and 9 mo.	Malaysian children (mix of Malay, Chinese and Indian) with all severities of asthma. Aged 6–17 y. Recruitment process not described.	At 6 mo there was no difference in A&E visits/unscheduled care [intervention 4 (SD 10.8) vs. control 6 (SD 21.1); *p* = 0.35]. At 6 mo there was no difference in proportion controlled (ACT ≥ 20 I: 81% vs. C: 87%; *p* = 0.50), with no exacerbations (ACT ≥ 20 I: 89% vs. C: 82%; *p* = 0.62) or in QoL [mean PAQLQ I: 6.11 (SD 0.88) vs. 6.11 (SD 1.09); *p* = 0.99].

*Abbreviations*: *A&E* accident and emergency, *ACQ* Asthma Control Questionnaire, *ACT* Asthma Control Test, *AQLQ* Asthma-related Quality of Life Questionnaire, *C* control, *CI* confidence interval, *FEV*_*1*_ forced expiratory volume in one second, *FU* follow-up, *GP* general practitioner, *I* intervention, *ICS* inhaled corticosteroid, *mAQLQ* mini Asthma-related Quality of Life Questionnaire, *MD* mean difference, *mo* months, *PAAP* personalised asthma action plan, *PAQLQ* paediatric asthma-related quality of life, *QoL* quality of life, *RCT* randomised controlled trial, *RR* risk ratio, *SD* standard deviation, *y* years

The final question (What is the effect of self-management on healthcare utilisation and costs?) was answered by a meta-analysis of the RECURSIVE RCT data. The primary analysis explored whether self-management support could reduce utilisation without compromising outcomes. Standardised mean differences (SMD) were computed using a random effects model meta-analysis due to anticipated heterogeneity. Four meta-analyses examined the effects of self-management support interventions on hospitalisation rates, A&E attendances, quality of life and total costs, respectively. We then constructed permutation plots of the data from the subset of trials reporting both utilisation (hospitalisation rates, A&E attendances or total costs) and health outcomes (quality of life). Further details about the analytic approach are described in the RECURSIVE report [14]. Forest plots and permutation plots [21] for the subset of studies reporting both health outcomes and utilisation outcomes were constructed in STATA version 14.

### Interpretation and end-of-project workshop

The PRISMS and RECURSIVE teams worked independently, but held regular teleconferences to enable synergies between the findings of the parallel reviews to be developed. Frequent meetings of the multidisciplinary teams aided interpretation of the emerging findings. Finally, we held an end-of-project stakeholder conference at which the findings and over-arching conclusions from PRISMS and RECURSIVE were presented to 34 multidisciplinary stakeholders, including people with LTCs, clinicians, commissioners, providers of healthcare services and policy-makers. Small discussion groups discussed and advised on practical implications for commissioning and providing services for people with LTCs.

### Lay involvement

The PRISMS project (which reviewed evidence from 14 LTCs) benefited from a lay collaborator who was involved from the inception of the project. She and other lay representatives from a range of LTC interest groups (including Asthma UK) contributed to an initial stakeholder workshop at which the choice of LTCs studied in the project and self-management interventions of interest were discussed. Lay members also participated in the end-of-project workshop (described above), which aided interpretation and guided dissemination. The PRIMER patient and public involvement group at the University of Manchester, UK, collaborated with the RECURSIVE project.

### Updating of searches prior to publication

We updated our PRISMS searches in January 2017 by undertaking forward citation of the original included reviews using Web of Science. Forward citation has been shown to be an efficient and effective method of identifying relevant papers in systematic reviews of complex and heterogeneous evidence [22]. We considered it was very unlikely that a subsequent systematic review or RCT would be published without citing at least one of the previously published reviews. One reviewer (HP) undertook focused data extraction of key findings, which were checked by MP. The additional data were added into the syntheses as appropriate. Had we identified studies that substantially changed our conclusions we planned to undertake full duplicate data extraction, quality assessment and revise our synthesis.

## Results

### Description of the studies in the meta-review

**Fig. 1:**
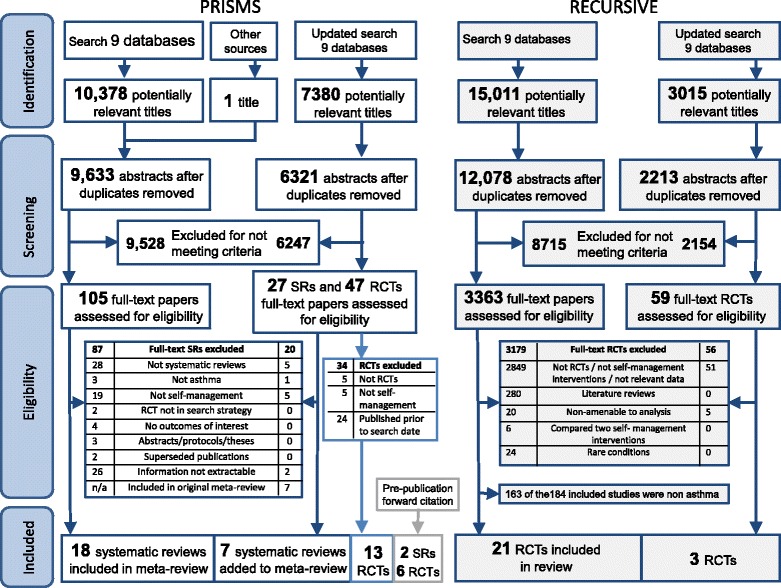
PRISMA flowchart. Note: The initial RECURSIVE search included all long-term conditions: papers reporting asthma randomised controlled trials (*RCTs*) were identified from 184 studies included in the full RECURSIVE report [14]

Figure 1 illustrates the PRISMA flow chart for both reviews. After removal of duplicates, 9633 references were identified from the initial PRISMS search and an additional 6321 from the update search. From these, 25 systematic reviews [23–47] were included in the PRISMS meta-review, representing data from 240 unique RCTs. The year of review publication ranged from 1995 to 2013, and included RCTs dated from 1979 to 2013. In addition we included 13 RCTs published since the last search dates of the included reviews (2010 for children, 2012 for adults and 2011 for ethnic groups; see Additional file 2 for details) [48–60]. (For clarity we refer to these as “update RCTs”.) A further two systematic reviews (which included a further four RCTs) [61, 62] and six RCTs [63–68] were added after the pre-publication update. The RECURSIVE study included 24 RCTs with publication dates from 1993 to 2015 [49, 69–91].

After excluding overlap, this represents 270 unique trials undertaken in at least 29 high- or middle-income countries: Argentina, Australia, Belgium, Brazil, Canada, Chile, Denmark, Finland, France, Germany, India, Israel, Italy, Jordan, Malaysia, Malta, Netherlands, New Zealand, Norway, Russia, Spain, Sweden, Switzerland, Taiwan, Trinidad, Turkey, UK, USA and Venezuela.

In the 18 systematic reviews that reported the duration of follow-up in their included RCTs [23–25, 27–29, 33, 35, 38–40, 42–47, 61], the modal duration (in 10 of the reviews) was 12 months, with only 3% of reported RCTs falling outside the range of 3–24 months. The update RCTs had a similar profile, with 6 of 13 update RCTs having a duration of 12 months (range 3–30 months).

### Study quality and weight of evidence

Taking into consideration both study quality and total population size, 10 PRISMS reviews received an evidence weighting of three stars [27, 31, 32, 36–38, 40, 41, 43, 46], 13 were weighted two star [23–26, 29, 30, 33–35, 39, 42, 44, 45] and two were weighted one star [28, 47]. Of the PRISMS update RCTs, four were judged to be at low risk of bias [50–52, 58], five at high risk of bias [48, 49, 55, 59, 60] and in four the risk of bias was unclear [53, 54, 56, 57]. Allocation concealment was judged as adequate in six of the 24 asthma studies included in the RECURSIVE review [74, 76, 80, 83–85]. Study quality is indicated in the first columns of Tables 3, 4 and 5, with details of the quality assessments in Additional file 4.

**Table 5:** Summary table of studies included in the RECURSIVE health economic analysis

Reference; Country; Allocation concealment	Study type; Participants, n; Intervention(s)	Comparison	Target group(s)	Health economic results				Formal health economic evaluation, cost-effectiveness (societal and health service perspective)
				Quality of life/asthma control	Healthcare utilisation (hospitalisation)	Total healthcare costs	Unscheduled care	
Baptist 2013 [49] US Concealment not adequate	RCT n = 70 Personalised 6-session self-regulation education.	Usual care. FU: 12 mo	Older adults with asthma (>65 y). Mean age 74 y. 14% male.	Proportion with ACQ <0.75 was greater in I group than C group [I: 13 (41.9%) vs. C: 5 (15.6%)].	I group had fewer hospitalisations (I: 0 vs. C: 4; *p* = 0.04).	n/a	No difference in A&E visits (I: 1 vs. C: 2; *p* = 0.58). I group had fewer unscheduled visits (I: 6 vs. C:14; *p* = 0.048).	n/a
Castro 2003 [69] US Concealment not adequate	RCT n = 96 Education, psychosocial support, PAAP and co-ordination of care.	Usual (private) primary care. FU: 12 mo	Inpatients, adults with asthma. Mean age 38 y. 15% male.	No between-group difference in mean AQLQ [I: 4.0 (SD 1.3) vs. C: 3.9 (SD 1.5); *p* = 0.55].	I group had fewer re-admissions/patient [I: 0.4 (SD 0.9) vs. C: 0.9 (SD 1.5); *p* = 0.04].	I group had lower costs/patient [I: $5726 (SD 5679) vs. C: $12,188 (SD 19,352); MD $6,462; *p* = 0.03].	No between-group differences in number A&E visits/patient [I:1.9 (SD 4.3) vs. C: 1.4 (SD = 1.5); *p* = 0.52].	n/a
Clark 2007 [70] US Concealment not adequate	RCT n = 808 Self-regulation intervention; nurse telephone-delivered.	Usual care. FU: 12 mo	Adult women with asthma. Mean age 49 y. 100% female	No between-group difference in mean AQLQ [I: 2.1 (SD 0.9) vs. C: 2.1 (SD 0.9].	No between-group difference in admissions/patient [I: 0.2 (SD 0.7) vs. C: 0.1 (SD 0.5)]	n/a	I group had greater reduction in unscheduled visits [mean change: I: −0.63 (SD 2.4) vs. C: −0.24 (SD 1.5)].	n/a
de Oliveira 1999 [71] Brazil Concealment not adequate	RCT n = 52 Outpatient education programme, including a written PAAP.	Usual care. FU: 6 mo	Adults; moderate to severe asthma. Mean age 38 y. 15% male.	No between-group differences in QoL score [I: 28 (SD 17) vs. C: 50 (SD 15); *p* = 0.0005].	No between-group differences in admissions/patient [I: 0 vs. C: 0.5 (SD 0.8); *p* = 0.08].	n/a	I group had fewer A&E visits/patient [I: 0.7 (SD 1.0) vs. C: 2 (SD 2)].	n/a
Gallefoss 2001 [72] Norway Concealment not adequate	RCT n = 78 Group-plus individual self-management education with a written PAAP.	Usual primary care. FU: 12 mo	Adults with asthma. Mean age 44 y. 21% male.	Better QoL (SGRQ) in I group at 12 mo [I: 20 (SD 15) vs. C: 36.5 (SD 18); MD 16.3, 95% CI 16.3–24.4]	n/a	No between-group differences in total costs (in NOK) [I: 10,500 (SD 20,500) vs. C: 16,000 (SD 35,400); *p* = 0.510].	n/a	Incremental SGRQ gain 16.3; health costs difference NOK1900; all cost diff NOK −5500.
Gruffydd-Jones 2005 [73] UK Concealment not adequate	RCT n = 174 Targeted nurse-led telephone reviews, including PAAPs.	Usual primary care. FU: 12 mo	Adults with asthma. Mean age 50 y. 40% male.	No between-group difference in mean change in ACQ [I: −0.11 (95% CI −032 to 0.11) vs. C: −0.18 (95% CI −0.38 to 0.02); *p* = 0.349].	n/a	No between-group difference in total costs [I: £209.85 (SD 220.94) vs. C: £333.85 (SD 410.64); MD £122.35; *p* = 0.071].	n/a	n/a
Honkoop 2015 [74] Netherlands Adequate concealment	RCT n = 611 Nurse-led care to symptom control (I) (or FeNO controlled).	Usual care (partially controlled). FU: 12 mo	Adults with asthma. Mean age 40 y. 28% male.	No between-group difference in EQ5D (QALYs) (I: 0.91 vs. C: 0.89; MD 0.01, 95% CI −0.02 to 0.04).	n/a	No between-group difference in total costs [I: $4591 vs. C: $4180; MD $411, 95% CI −904 to 1797; *p* > 0.05].	n/a	n/a
Kauppinen 1998 [75] Finland Concealment not adequate	RCT n = 162 Intensive education (use of inhaled drugs, PEF, monitoring and PAAP).	Conventional education. FU: 12 mo	Adults, newly diagnosed asthma. Mean age 43 y. 44% male.	No between-group difference in 15D [I: 0.93 (95% CI 0.90–0.94) vs. C: 0.91 (95% CI 0.89 to 0.94); *p* = 0.47].	n/a	I group had greater total costs than control [I: £345 (95% CI 247–1758) vs. C: £294 (95% CI 0–8078); p < 0.001].	n/a	Intensive education: incremental gain of 0.02 15D. Incremental difference in health costs of £51.
Krieger 2015 [76] US Adequate concealment	RCT n = 366 Community health worker-supported self-management.	Usual care. FU: 12 mo	Adults with asthma. Mean age 41 y. 27% male.	Intervention improved QoL. Mean change in mAQLQ (I: 0.95 vs. C: 0.36; MD 0.50, 95% CI 0.28–0.71; *p* <0.001).	No difference in mean change in number of urgent care episodes. (I: −1.50 vs. C: −1.60; difference 0.09, 95% CI −0.59 to 0.73; *p* = 0.78.)	n/a	n/a	n/a
Lahdensuo 1996 [77] Finland Concealment not adequate	RCT n = 122 Self-management, including breathing exercises, education and PEF monitoring.	Traditional treatment. FU: 12 m	Adults with asthma. Mean age 43 y. 48% male.	Intervention improved QoL SGRQ (symptom domain) [I: 16.6 (SD 15.9) vs. C: 8.4 (SD 18.4); *p* = 0.009].	n/a	n/a	I group had fewer unscheduled care visits/patient/year (I: 0.5 vs. C:1; *p* = 0.04).	n/a
Levy 2000 [78] UK Concealment not adequate	RCT n = 211 Structured education with PAAP by A&E specialist nurses.	Usual primary care. FU: 6 mo	Adults with asthma. Mean age 40 yrs. 43% male.	No between-group difference in SGRQ (I: 30.25 vs. C: 28.73; MD 1.52, 95% CI −4.05 to 7.09).	No between-group difference in hospital consultations [median (IQR) I: 0 (1–3) vs. C: 0 (1–6); *p* = 0.17].	n/a	No between-group difference in GP consultations [median (IQR) I: 0 (1–7) vs. C: 0 (1–7); *p* = 0.14].	n/a
Mancuso 2011 [79] US Concealment not adequate	RCT n = 296 Self-management workbook, behavioural contract, telephone calls.	Information/PEF training. FU: 12 mo	Adults attending A&E with asthma. Mean age 43 y. 23% male.	No between-group difference in change in AQLQ at 1 y (I: 0.04 vs. C: 0.18; MD 0.22, 95% CI −0.15 to 0.60).	n/a	n/a	No between-group difference in proportion with A&E visits (I: 13% vs. C: 11%).	n/a
McLean 2003 [80] Canada Adequate concealment	RCT n = 225 Pharmacist-led self-management, with PAAP.	Usual pharmacist care. FU: 7 mo	Adults with asthma. Mean age 38 y. 47% male.	Intervention improved QoL as mean AQLQ (I: 5.13 vs. C: 4.40; *p* = 0.0001).	No between-group difference in hospitalisations (I: 0.078 vs. C: 0.16; *p* = 0.94).	Intervention reduced total costs (costs per patient I: $150 vs. C: $351).	No between-group difference in A&E visits (I: 0.04 vs. C: 0.21; *p* = 0.48).	n/a
Moudgil 2000 [81] UK Concealment not adequate	RCT n = 689 Individual education and optimisation of drug therapy.	Usual primary care. FU: 12 mo	Adults with asthma. Mean age 35 y. 47% male.	Greater improvement in QoL in I group (MD in change in AQLQ 0.22 , 95% CI 0.15–0.29).	No between-group difference in hospitalisations (OR 0.51, 95% CI 0.22–1.14).	n/a	No between-group difference in A&E visits (OR 0.63, 95% CI 0.23–1.68).	n/a
Pilotto 2004 [82] Australia Concealment not adequate	Cluster RCT n = 170 Nurse-run asthma clinics including provision of PAAPs.	Usual primary care. FU: 9 mo	Adults with asthma. Mean age 50 y. 48% male.	No between-group difference in SGRQ (I: 27.3 vs. C: 27.0; MD −0.5 (−4.0 to 2.9).	No between-group difference in number admitted (I: 2 vs. C: 0; *p* = 0.499).	n/a	No between-group difference in number attending A&E (I: 2 vs. C: 0; *p* = 0.499).	n/a
Pinnock 2003 [83] UK Adequate concealment	RCT n = 278 Nurse-delivered, routine telephone review.	Usual primary care. FU: 3 mo	Adults with asthma. Mean age 57 y. 41% male.	No between-group difference in mAQLQ (I: 5.17 vs. C: 5.17; MD 0.22, 95% CI −0.15 to 0.60).	No patients in either group had a hospital admission for asthma.	n/a	No patients in either group had an A&E attendance for asthma	n/a
Price 2004 [84] UK Adequate concealment	Cluster RCT n = 1553 Use of PAAPs with adjustable maintenance dosing.	Usual care. FU: 3 mo	Adults with asthma. Mean age 48 y. 41% male.	No between-group difference in proportion with improved QoL (I: 22.5% vs. C: 23.6%).	No between-group difference in hospital admissions (I: 2 vs. C: 2).	Intervention reduced total costs (cost/day/patient I: £1.13 vs. C: £1.31; MD − £0.17, 95% CI -£0.11 to -£0.23).	No between-group difference in A&E visits (I: 5 vs. C: 11).	n/a
Ryan 2012 [85] UK Adequate concealment	RCT n = 288 Mobile phone supported self-management.	Paper-based PAAPs. FU: 6 mo	Adults with asthma. Mean age 52 y. 41% male.	No between-group difference in mean change in mAQLQ (difference −0.10, 95% CI −0.16 to 0.34).	No between-group difference in hospital admissions for asthma (I: 3 vs. C: 1).	n/a	No between-group difference in A&E attendances for asthma (I: 3 vs. C: 0).	n/a
Schermer 2002 [86] Netherlands Concealment not adequate	RCT n = 193 Guided self-management with education and PEF monitoring.	Usual primary care. FU: 24 mo	Adults with asthma. Mean age 39 y. 42% male.	No between-group difference in total AQLQ (I: 39 vs. C: 29; MD 10, 95% CI −3 to 23).	No hospital admissions in either treatment group.	No between-group difference in total costs (I: €1084 vs. C: €1097; MD − €13).	No A&E visits in either treatment group.	Incremental QALY gain 0.015. Incremental total cost − €13. Incremental health cost €11. Incremental health ICER €33/QALY.
Shelledy 2009 [87] US Concealment not adequate	RCT n = 166 Nurse- (N) vs. respiratory therapist-(RT) led education and management.	Usual primary care. FU: 6 mo	Adults: A&E or admitted with asthma. Mean age 44 y. 22% male.	RT I group had greater change in SGRQ [I(RT) −11.0 vs. I(N) −6.0 vs. C: −2.5, *p* < 0.05).	I group had fewer hospitalisations [I(RT): 0.04 vs. I(N): 0 vs. C: 0.20; *p* < 0.05).	I group had lower hospitalisation costs [I(RT): $202 vs. I(N): $0 vs. C: $1065; *p* < 0.05].	No between-group difference in A&E visits [I(RT): 0.09 vs. I(N): 0.26 vs. C: 0.37)].	n/a
Sundberg 2005 [88] Sweden Concealment not adequate	RCT n = 97 Interactive computer-based education plus nurse support.	Usual care. FU: 12 mo	Young adults with asthma. Mean age 19 y. 55% male.	No between-group difference in Living with Asthma Questionnaire (I: 163.6 vs. C: 166.2, *p* > 0.05).	No between-group difference in hospital admissions (1 admission in each group).	n/a	No between-group difference in A&E visits (I: 17 vs. C: 16).	n/a
van der Meer 2011 [89] Netherlands Concealment not adequate	RCT n = 200 Internet-based self-management programme, including electronic PAAP.	Usual outpatient care. FU: 12 mo	Adults with asthma. Mean age 37 y. 55% male.	No between-group difference in EQ5D (I: 0.93 vs. C: 0.89; difference 0.006, 95% CI −0.042 to 0.054).	No between-group difference in hospital admissions (mean cost: I: $571 vs. C: $589; MD −17; *p* = 0.95).	No between-group difference in total healthcare costs (I: $2555 vs. C: $2518; MD − $37; *p* = 0.94).	n/a	Incremental QALY gain 0.024. Incremental total cost $641. Incremental health cost $37. Incremental health ICER $1541/QALY.
Yilmaz 2002 [90] Turkey Concealment not adequate	RCT n = 80 Outpatient clinic, special education programme.	Usual primary care. FU: 36 mo	Adults with asthma. Mean age 29 y. 37% male.	I group had greater improvements in AQLQ (I: 197.1 vs. C: 176.7; *p* = 0.009).	No between-group difference in hospitalisations (I: 0 vs. C: 4); *p* > 0.05.	n/a	I group had fewer A&E visits (I: 0 vs. C: 7; *p* = 0.01).	n/a
Yoon 1993 [91] Australia Concealment not adequate	RCT n = 76 Brief, group-based, education with a PAAP.	Usual outpatient care. FU: 10 mo	Inpatient adults. Mean age not reported. 28% male.	No between-group difference in QoL [I: 4.0 (SD 4.38) vs. C: 3.96 (SD = 3.34); *p* > 0.05).	I group had fewer participants with hospital admissions (I: 1 vs. C: 7; *p* < 0.001).	n/a	No between-group difference in A&E visits (I: 3 vs. C: 7).	n/a

*Abbreviations*: *A&E* accident and emergency, *ACQ* Asthma Control Questionnaire, *AQLQ* Asthma Quality Of Life Questionnaire, *C* control, *CI* confidence interval, *EQ5D* EuroQol Five Dimensions Questionnaire, *FeNO* fractional exhaled nitric oxide, *FU* follow-up, *GP* general practitioner, *I* intervention, *ICER* incremental cost-effectiveness ratio, *IQR* interquartile range, *mAQAL* mini Asthma Quality Of Life Questionnaire, *MD* mean difference, *mo* month, *N* nurse, *n/a* not available, *PAAP* personalised asthma action plan, *PEF* peak expiratory flow, *QALY* quality-adjusted life years, *QoL* quality of life, *RCT* randomised controlled trial, *RT* respiratory therapist, *SD* standard deviation, *SGRQ* St George’s Respiratory Questionnaire, *y* year

### Overview of presentation of results

Tables 3, 4, 5 and 6 provide summaries of the studies included in the PRISMS meta-review, update RCTs, the RECURSIVE review and pre-publication update with more detailed tables in Additional file 5.

**Table 6:** Focused data extraction from additional studies identified by forward citation prior to publication

Reference; RCTs, n; Participants, n; Date range RCTs	Comparison	Relevance to meta-review questions:	Interventions included	Target group(s)	Synthesis	Main results
		What is the impact? Target groups? Which components? Context?				
Systematic reviews						
Coelho 2016 [61] 17 RCTs; 5879 participants RCTs 2005–2013	School-based asthma education vs. usual care. FU: minimum 1 mo	Target: Schoolchildren	Educational interventions to individuals, groups or classes by healthcare professionals, teachers, educators and/or IT.	Schoolchildren with asthma and/or whole school.	Narrative analysis	6/17 showed a reduction in unscheduled care; 5/17 showed a reduction of the asthma symptoms; 5/17 reduced school absenteeism; 7/17 improved QoL of the individuals; 8/17 showed that asthma education improved knowledge.
McLean 2016 [62] 5 RCTs 595 participants RCTs 2011–2013	Interactive digital interventions vs. usual care. FU: 10 weeks to 12 mo	Impact Components: Technology-based interventions	Interactive intervention (i.e. entering data, receiving tailored feedback, making choices) accessed through an app that provides self-management information.	Adults (≥16 y) with asthma.	Meta-analysis	Meta-analyses (3 studies) showed no significant difference in asthma control (SMD 0.21, 95% CI −0.05 to 0.42) or asthma QoL (SMD 0.05, 95% CI −0.22 to 0.32) but heterogeneity was very high. Removal of the outlier study reduced heterogeneity and indicated significant improvement for both asthma control (SMD 0.54, 95% CI 0.22–0.86) and asthma QoL (SMD 0.45, 95% CI 0.13–0.77).
**Randomised trials**						
Hoskins 2016 [63] 48 participants	Goal-setting + SM/PAAPs vs. usual care.	Components: Goal-setting	Practice asthma nurses trained in goal-setting approach.	Primary care patients due a review.	Cluster feasibility RCT. FU: 6 mo	Difficulty recruiting: 10/124 practices participated and 48 patients. No between-group difference in QoL [mAQLQ I: 6.20 (SD 0.76, 95% CI 5.76–6.65) vs. C: 6.1 (SD 0.81, 95% CI 5.63–6.57), MD 0.1].
Morawska 2016 [64] 107 participants	Generic parenting skills vs usual care.	Components: Parenting skills	Parenting skills for managing LTCs + asthma ‘take-home tips sheets’.	Parents of children 2–10 y with asthma and/or eczema.	RCT. FU: 6 mo	Between-group improvement in parents’ self-efficacy and childs’ ‘eczema behaviour’, but not equivalent asthma outcomes. Parent and family generic QoL improved (*p* = 0.01).
Plaza 2015 [65] 230 participants	Trained practices (I) vs. specialist unit (I^s^) vs. usual care (C).	Impact: Components: Education programme	Basic information on asthma, inhaler technique; provision of a PAAP.	Adults with persistent asthma.	Cluster RCT. FU: 12 mo	I groups had fewer unscheduled visits [I: 0.8 (SD 1.4) and I^s^: 0.3 (SD 0.7) vs. C:1.3 (SD 1.7); *p* = 0.001], and greater improvements in asthma control (*p* = 0.042) and QoL (*p* = 0.019).
Rice 2015 [66] 711 participants	PAAP + inpatient lay educator vs. PAAP.	Components: Inpatient lay educator	Encourage FU attendance, build self-efficacy, set goals, overcome barriers.	Children 2–17 y admitted with asthma.	RCT. FU: 1 mo	No difference in attendance at FU appointment. I group had greater preventer use (OR 2.4, 95% CI 1.3–4.2), PAAP ownership (OR 2.0, 95% CI 1.3–3.0) and improved self-efficacy (*p* = 0.04).
Yeh 2016 [67] 76 participants	Family programme (+PAAP) vs. usual care (+PAAP).	Components: Family empowerment	Family empowerment to reduce parental stress, increase family functioning.	Children 6–12 y with asthma.	RCT. FU: 3 mo	I families had reduced parental stress index (*p* = 0.026) and improved family environment scores (*p* < 0.0001), improved lung function, less disturbed sleep, less cough but no difference in wheeze.
Zairina 2016 [68] 72 participants	Telehealth supported PAAP vs. usual care.	Components: Telehealth	Telehealth (FEV_1_, symptoms) monitored weekly.	Pregnant women with moderate/severe asthma	RCT. FU: 6 mo	Telehealth improved ACQ [MD 0.36 (SD 0.15, 95% CI −0.66 to −0.07)] and mAQLQ [MD 0.72 (SD 0.22; 95% CI 0.29–1.16)]. No difference in perinatal outcomes.

*Abbreviations*: *ACQ* Asthma Control Questionnaire, *AQLQ* Asthma Quality Of Life Questionnaire, *C* control, *CI* confidence interval, *FEV*_*1*_ forced expiratory volume in one second, *FU* follow-up, *I* intervention, *LTC* long-term condition, *mAQAL* mini Asthma Quality Of Life Questionnaire, *MD* mean difference, *mo* month, *OR* odds ratio, *PAAP* personalised asthma action plan, *QoL* quality of life, *RCT* randomised controlled trial, *SD* standard deviation, *SMD* standardised mean difference, *y* year

### Can supported self-management reduce the use of healthcare resources and improve asthma control?

#### Use of healthcare resources

**Fig. 2:**
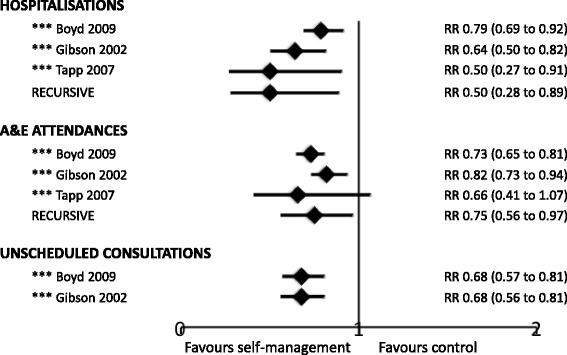
Meta-Forest plot of healthcare resource use from meta-analyses. This meta-Forest plot displays the summary data from the PRISMS systematic reviews that reported relative risk (*RR*). Note that meta-analysis is inappropriate at meta-review level owing to the overlap of included randomised controlled trials between reviews

Figure 2 is a meta-Forest plot illustrating the meta-analyses (including three PRISMS 3* reviews and RECURSIVE) that report relative risks of admissions, A&E attendances and/or unscheduled consultations [27, 31, 38]. Treatment event rates from the meta-analyses are in Table 7. These results suggest similar effects in adults [38], children [27] and mixed populations [31].

**Table 7:** Treatment event rates from the meta-analyses

	Events/total participants		Percentage of participants with the event	
	Intervention	Control	Intervention	Control
Proportion hospitalised				
Boyd 2009 [27]***	276/2009	351/2010	13.7	17.4
Gibson 2002 [31]***	85/1200	139/1218	7.1	11.4
Tapp 2007 [38]***	40/286	74/286	14.0	25.9
RECURSIVE	80/1727	124/1734	4.6	7.2
Proportion with A&E attendances				
Boyd 2009 [27]***	337/1505	462/1503	22.4	30.7
Gibson 2002 [31]***	291/1457	354/1445	20.0	24.5
Tapp 2007 [38]***	74/472	104/474	15.7	22.0
RECURSIVE	153/1171	227/1170	13.1	19.4
Proportion with unscheduled visits				
Boyd 2009 [27]***	128/515	181/494	24.9	36.6
Gibson 2002 [31]***	112/784	170/772	14.3	22.0

*Abbreviations*: *A&E* accident and emergency

Hospitalisations were reported in 12 reviews [25–29, 31, 35, 38, 40, 41, 44, 46]. Six meta-analyses (four 3*, two 2*) showed that self-management support interventions led to fewer hospital admissions [25–27, 31, 38, 41]. Six narrative reviews of variable quality, reporting heterogeneous interventions, showed inconsistent effects on hospitalisations [28, 29, 35, 40, 44, 46].

Ten reviews reported A&E attendances [25–27, 29, 31, 35, 38, 40, 44, 46]. Four meta-analyses (three 3* [27, 31, 38], one 2* [26]) reported a reduction in A&E attendances in the self-management intervention compared to control groups. Four narrative reviews (one 3* [46], three 2* [25, 35, 44]) showed a reduction in A&E attendances in at least half of their included RCTs; one 3* review showed inconsistent results [40], and one 2* review showed no benefit on A&E attendances [29].

Of the eight reviews that reported unscheduled care [24, 27, 28, 31, 34, 35, 43, 44], three 3* meta-analyses reported fewer unscheduled consultations in participants who received a self-management intervention when compared to control [27, 31, 43]. Furthermore, three 2* narrative reviews reported that self-management reduced unscheduled care in at least half their included trials [34, 35, 44]. The remaining two small or poor quality reviews had inconsistent results [24, 28].

#### Asthma control

Of the 10 reviews that reported measures of control [24, 28, 30, 31, 34, 35, 38, 41, 44, 46], three meta-analyses (two 3* [31, 41], one 2* [24]) and three narrative reviews [28, 35, 44] reported a reduction in symptoms in participants who received self-management interventions compared to control groups. The other four narrative reviews (two 3* [30, 34], two 2* [38, 46]) had inconsistent results [30, 34, 38] or showed no benefit on symptom control [46]. The broader concept of quality of life was reported as improved in some reviews [25, 30, 34, 46], but not others [27, 29, 40, 44].

Six reviews reported a reduction in days missed from school or work [24, 29–31, 38, 41]. Two 3* meta-analyses [31, 41], two small reviews each with only one RCT [24, 29] and five of the 13 RCTs in a 2* narrative synthesis of school-based interventions [30] concluded that self-management interventions reduced absenteeism. A single RCT reported in a 3* narrative review in adults concluded that asthma education following A&E attendance had no effect on absenteeism [38].

### In which target groups has supported self-management been shown to work?

The systematic reviews encompassed a broad range of populations in diverse healthcare and demographic settings with consistently positive findings. For example, the reviews included all ages [28, 31] or only children [24, 26, 27, 29, 30, 35, 40, 41] or adults [34, 38, 43, 46]. Some focused on lower socioeconomic groups [35, 40] or ethnic minority communities [25, 29, 35]. The reviews and RCTs identified in the PRISMS update typically built on this extensive generic evidence base and investigated interventions targeting specific groups such as urban [52, 54], rural [53], deprived communities [46, 52, 54], cultural groups [46, 54, 55, 60], adolescents [48, 54, 56, 57] or older adults [49, 51]. Table 8 summarises the key strategies used in trials to tailor interventions, or their mode of delivery, to different groups.

**Table 8:** Tailoring of self-management support for targeted populations

Group	Key strategies	Description of tailoring of self-management intervention	Relevant systematic reviews/update RCTs	Evidence
Cultural groups	Cultural tailoring	Culturally orientated self-management programmes including individual sessions with language-appropriate asthma educators, videos/workbooks featuring culturally appropriate role models, education appropriate to socioeconomic context, strategies for use of local healthcare services, asthma action plans.	**Bailey 2009 [25] Adults and children from minority groups	Culture-specific programmes are more effective than generic programmes in improving QoL, knowledge and asthma control but not all asthma outcomes.
		Culturally tailored, community-based interventions in which healthcare providers (pharmacists, asthma educator, social workers, respiratory nurses) provided language-appropriate education programmes including health literacy-focused teaching, use of videos, asthma physiology and management, inhaler technique, PAAP.	***Press 2012 [46] Adults from minority groups in the USA	The 5 (of 15) education studies that were culturally tailored showed reduced use of unscheduled care and improved QoL, but this is not compared to non-tailored interventions.
		Internet-based programme developed to deliver education and a behaviour change intervention to African-Americans adolescents. Strategies include voice-overs to accommodate literacy limitations and advice delivered by a ‘disc jockey’.	(RCT) Joseph 2013 [54] Young teens	The intervention reduced symptom-free days but had no effect on A&E visits/hospitalisations.
	Community workers	Community health worker from the same/very similar community as participating families provided individually tailored education at home visits. Topics included asthma, lifestyle and trigger avoidance, with resources to reduce allergen exposure and smoking cessation support.	**Postma 2009 [35] Ethnic minority children with asthma	Interventions involving community health workers reduced emergency and urgent care use in some but not all studies.
		Indigenous healthcare workers provided personalised, child-friendly, culturally appropriate education materials at home visits to reinforce clinical consultations.	**Chang 2010 [29] Ethnic minority children with asthma	The involvement of indigenous healthcare workers in asthma programmes (1 RCT) improved control and QoL but not unscheduled care.
A&E attendees	Education during the A&E attendance	Education sessions conducted by asthma or A&E nurses, or, less often, respiratory specialists or a physiotherapist. Content varied, usually including triggers, PAAPs and/or inhaler technique.	***Tapp 2007 [38] Adult A&E attendees	Education delivered in A&E reduced subsequent hospital admissions but not A&E attendances. Effect on QoL was inconsistent.
		PAAP, completed by the A&E physician, coupled with the prescription provided on discharge from A&E.	(RCT) Ducharme 2011 [50] Children 1–17 y, A&E attendees	Provision of a PAAP increased patient adherence to steroids (oral/inhaled), and improved asthma control.
	Education after A&E	Education delivered by a healthcare professional or asthma educator shortly after an A&E attendance, including triggers and PAAPs, to the child and their carers.	***Boyd 2009 [27] Children, A&E attendees	Asthma education reduced A&E attendances and admissions, but had no effect on QoL.
Schoolchildren	School-based programmes	School-based group education, the majority including education for classmates without asthma.	**Coffman 2009 [30] Children	The intervention improves knowledge, self-efficacy and self-management behaviours, but inconsistent effect on asthma control.
		16 short group educational sessions, including strategies for problem solving, delivered in the school lunch break.	Horner 2014 [53] Grades 2–5 (7–11 y)	Compared to generic health education, the intervention improved self-efficacy but had no effect on admissions, A&E visits or QoL.
	Peer-led programmes	Year 11 pupils were trained to deliver the school-based asthma educational lessons to younger pupils.	Al-Sheyab 2012 [48] Adolescents	Compared to children in control schools, knowledge and QoL improved. Also increased self-efficacy to resist smoking.
		Asthma self-management skills and psychosocial skills taught at a day camp by peer leaders followed by monthly peer telephone contact.	Rhee 2011 [56] Adolescents 13–17 y	The intervention group had improved QoL and positive ‘attitude to illness’ compared to those attending adult-led camps.
	Technology-based	Internet-based interventions, delivered at home, clinic or school, which delivered a psycho-educational programme involving information and skills training modules targeting improved health outcomes.	**Stinson 2009 [47] Children 4–17 y	The majority of studies reported improvement in symptoms, but impact on other outcomes was inconsistent.
		Theoretically based asthma computer programme with core modules (adherence, inhaler use, smoking reduction), with tailored sub-modules to address specific behavioural traits.	Joseph 2013 [54] 9–12 grade (14–18 y)	The intervention improved symptom control, but had no effect on A&E visits/hospitalisations.
		Internet-based self-management programme covering education, self-monitoring and an electronic action plan, and encouraging regular medical review. Supported by 2 face-to-face groups.	Rikkers-Mutsaerts 2012 [57] Adolescents 12–18 y	QoL and asthma control improved compared to usual care, but no difference in use of healthcare resources.
Elderly	Goal-setting	Six-session programme, conducted by a health educator in groups (*n* = 3) and telephone calls (*n* = 3). Participants selected an asthma-specific goal, identified problems and addressed potential barriers.	(RCT) Baptist 2013 [49] ≥65 y	Compared to education alone, the intervention improved asthma control and QoL, but not unscheduled care.
	Addressing individual concerns	Specific concerns, identified with the Patient Assessment and Concerns Tool (PACT), were addressed in an hour-long session. Both groups had standard education (inhaler technique, PAAP).	(RCT) Goeman 2013 [51] ≥55 y	Compared to usual care, asthma control and QoL was improved by education tailored to individual patient concerns and unmet needs.

*Abbreviations*: *A&E* accident and emergency, *PAAP* personalised asthma action plan, *QoL* quality of life, *RCT* randomised controlled trial

#### Cultural groups

Four reviews explored the impact of self-management in cultural groups [25, 29, 35, 46]. A 2* meta-analysis reported that culture-specific programmes reduced hospitalisations in children and improved quality of life in adults compared to generic interventions [25]. A 3* narrative synthesis found only two RCTs testing culturally tailored interventions, one of which improved quality of life [46]. The involvement of community health workers reduced use of healthcare resources in two thirds, and improved symptoms in all seven RCTs included in a 2* narrative review [35]. An inpatient visit from a lay educator to Black or Latino children improved self-efficacy and action plan ownership 1 month post-discharge [66]. In contrast, three generic interventions in US minority populations showed no improvement [46]. Update RCTs, some underpowered, in indigenous populations had inconsistent outcomes [29, 48, 55, 60].

#### A&E attendees

Two 3* meta-analyses demonstrated reduced use of healthcare resources (admissions, A&E attendances and unscheduled consultations) in adults recruited during A&E attendance (13 RCTs) [38] and in children with a history of A&E attendance in the previous 12 months (38 RCTs) [27]. Neither review found improved markers of asthma control [27, 38], though an update RCT in paediatric A&E attendees (low risk of bias) found that children discharged with an action plan had fewer symptoms at 28 days compared with usual care [50].

#### Specific age groups

School-based interventions [30], often using information technology-based programmes [30] or delivered by peers [48, 56], improved quality of life and, in some cases, reduced absenteeism [30, 48, 56, 61]. Generic parenting skills initiatives improved self-efficacy in families struggling to manage young children with asthma, with inconsistent effect on asthma outcomes [64, 67].

Two update RCTs reported interventions in older people that improved control and quality of life [49, 51], and one reduced use of unscheduled care [49]. A key feature of both complex interventions was a structured approach to tailoring in order to meet personal goals or address individual problems.

### Which components of supported self-management are important?

A 3* meta-analysis (36 RCTs; 6090 participants of all ages recruited from primary and secondary care settings) defined optimal self-management as education including advice on self-monitoring and a written action plan that was supported by regular professional review [31]. There is evidence that reducing the intensity of self-management education or level of clinical review may reduce its effectiveness [36].

#### Components of an action plan

The components of an action plan were further defined in two 3* and three 2* reviews [23, 24, 32, 36, 39]. In adults, self-monitoring based on peak flow or symptoms is equally effective [32, 36, 39]. In a comparison in children, symptom-based plans were more effective at reducing unscheduled healthcare [23], and equally effective at improving most measures of asthma control; the exception was days with symptoms, which were reduced more by peak-flow-based than symptom-based plans [23]. A 3* review concluded that action plans with between two and four action points, including recommendations on increasing inhaled corticosteroids and initiating oral corticosteroids, were consistently effective in reducing admissions and A&E attendances [32].

#### Behavioural change techniques

One 3* meta-analysis demonstrated that self-management interventions that incorporated specific behaviour change techniques reduced unscheduled care and improved control [43]. Meta-regression of the data from the 38 RCTs (7883 participants) concluded that active involvement of participants in the intervention was a key factor in reducing unscheduled healthcare [43]. More specifically, identifying individual behavioural traits (e.g. rebelliousness, low perceived emotional support) in adolescents enabled targeted use of behavioural change techniques [54]. A goal-setting approach proved challenging to implement in primary care settings [63].

#### Technology

Two 1* narrative reviews investigated computer- or internet-based interactive self-management programmes [28, 47]. The effect on healthcare utilisation was inconsistent, confirmed by a recent review identified in the pre-publication update [62], though both showed improvement in symptoms [28] and/or quality of life [28, 47]. Two update RCTs of web-based self-management programmes for adolescents also showed improved asthma control [54, 57], and an extended follow-up of RCT participants concluded that these effects could be sustained 18 months after conclusion of the trial [59]. Several school-based programmes used technology-based interventions to improve control and reduce absenteeism [30]. Supported self-management using mobile phone technology currently has a limited and inconclusive evidence base [42, 45], though a recent RCT in pregnancy demonstrated improved asthma control and quality of life [68].

### Which contextual factors influence effectiveness?

Resonating with the concept of ‘optimal’ self-management (education, an action plan and regular review) [31], a 3* meta-analysis identified that omitting regular review (1 RCT) or reducing intensity of education (1 RCT) was associated with a smaller reduction in unscheduled consultations [36]. A 2* meta-analysis analysed the findings of 18 RCTs (3006 participants) according to the components of the Chronic Care Model [92]. Interventions that included all four components had a greater effect on adherence to inhaled corticosteroids compared to trials including self-management unsupported by the organisational components [33].

#### Organisational role in promoting supported self-management

A 3* narrative review of 14 RCTs (4588 participants) concluded that proactive organisational systems can increase action plan ownership by promoting uptake of asthma reviews and implementing (and monitoring) structured management systems for asthma care [37]. A recent RCT of a structured approach to self-management education in both primary care and specialist units improved asthma control and reduced unscheduled care [65], and a large cluster RCT at low risk of bias showed an increased adherence to guidelines and reduced asthma symptoms by systematically providing individualised prompts to general practitioners and parents of children with asthma [52]. Automatically linking an action plan to prescriptions given to patients being discharged from A&E improved clinician management and patient uptake of steroid courses [50].

### What is the effect of supported self-management on healthcare utilisation and costs?

**Fig. 3:**
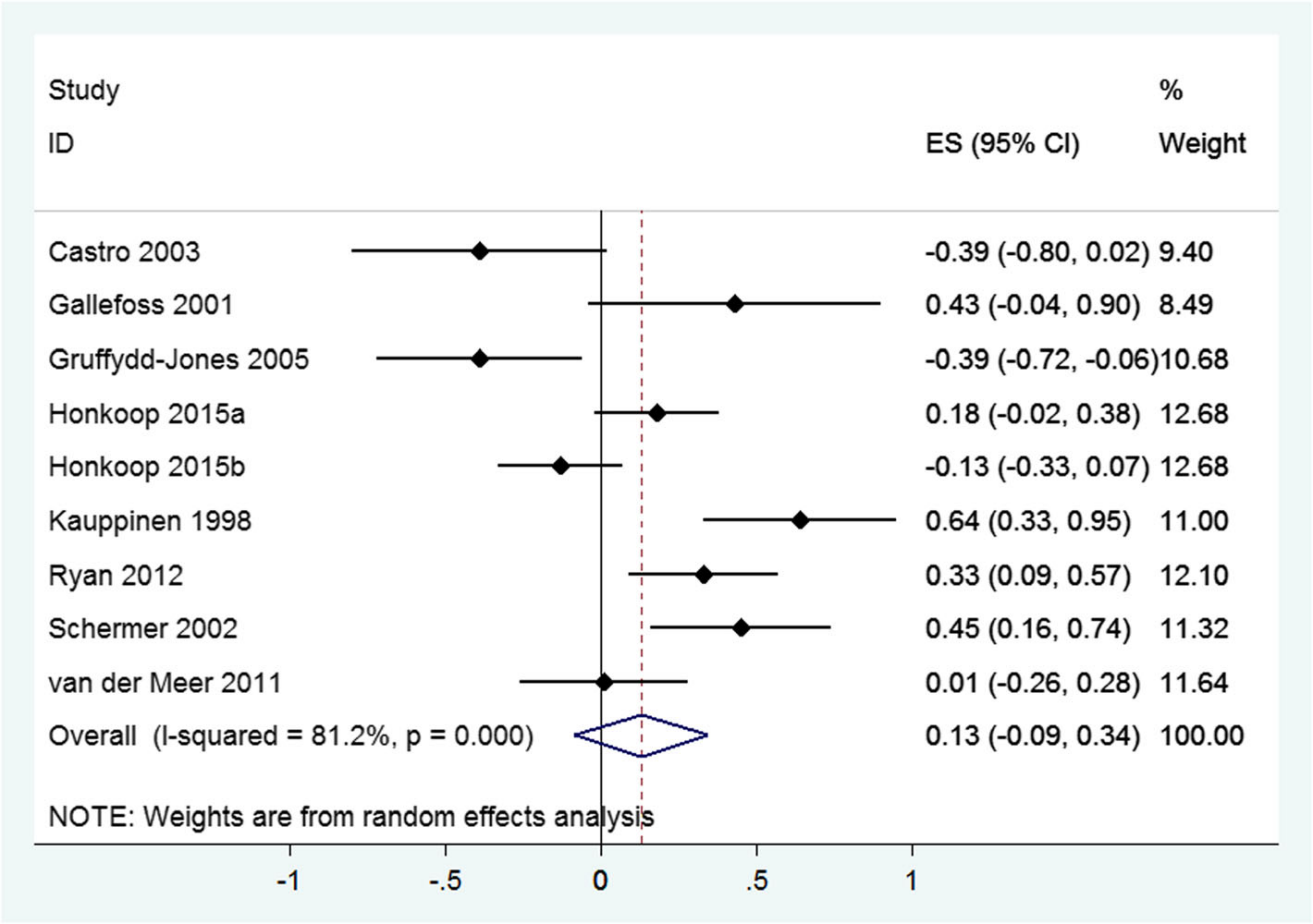
Meta-analysis of total costs. *CI* confidence interval, *ES* effect size

The RECURSIVE meta-analysis confirmed that self-management support interventions for people with asthma are associated with significant improvements in quality-of-life outcomes (SMD 0.26, 95% CI 0.12–0.39), significant small decreases in hospitalisation rates and costs (SMD −0.21, 95% CI −0.40 to −0.01), significant small decreases in A&E visits (SMD −0.25, 95% CI −0.49 to −0.01), and non-significant small increases in total healthcare costs (SMD 0.13, 95% CI −0.09 to 0.34). Figure 3 shows a Forest plot of the total costs.

#### What is the evidence that supported self-management for asthma can reduce costs without compromising outcomes?

**Fig. 4:**
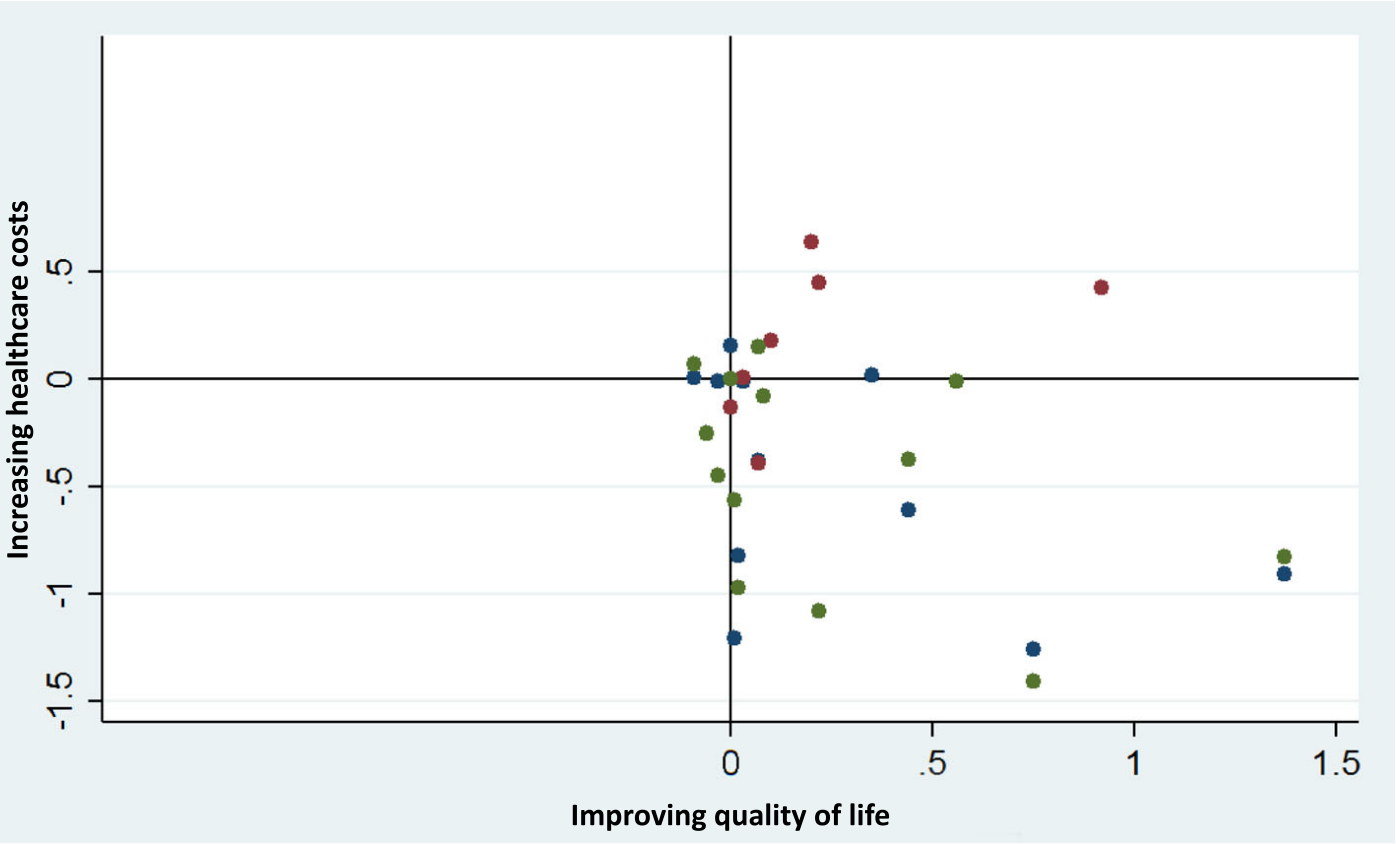
Permutation plot. Quality of life (x-axis), hospitalisations (y-axis *blue*) and total costs (y-axis *red*). In this permutation plot, the effects of self-management interventions on outcomes (quality of life) and utilisation (hospitalisations and total costs) can be visualised simultaneously by placing them in quadrants of the cost-effectiveness plane depending on the pattern of outcomes. Such plots identify studies in the appropriate quadrant (i.e. those that reduce costs without compromising outcomes) and those in problematic quadrants (i.e. those that reduce costs but also compromise outcomes, or those that compromise both outcomes and costs).

Figure 4 shows the overall permutation plot of the studies (n = 21) reporting data on both quality of life and healthcare utilisation. The majority of the studies on quality of life versus costs related to hospitalisations and A&E attendances were in the right-down quadrant, indicating cost-effectiveness (reduced healthcare utilisation and improved quality of life). However, in terms of total costs (n = 7), the picture was mixed with more studies around zero and the right-up quadrant, indicating that similar costs or small cost increases are necessary to achieve better quality of life.

#### What is the evidence that supported self-management for asthma is cost-effective?

Four studies applied formal economic analyses; two showed that self-management support interventions were dominant (i.e. significantly better health outcomes with significantly lower costs) [72, 86], and two produced non-significant ratios between costs and benefits at levels likely to appeal to decision-makers (better outcomes with non-significant increases in costs) [75, 89] (see Additional file 5 for more details).

Thus, the benefits derived by supported self-management interventions are associated with reductions in key areas of healthcare utilisation such as hospitalisations and A&E attendances and can be delivered at similar levels of total costs to usual care.

## Discussion

### Summary of findings

Extensive evidence (*n* = 270 RCTs) derived from a broad range of demographic and healthcare settings reveals that supported self-management can reduce hospitalisations, A&E attendances and unscheduled consultations, and improve markers of control and quality of life for people with asthma. Core components of effective self-management are education, provision of an action plan and the support of regular professional review. Effectiveness has been demonstrated in diverse cultural, clinical and demographic groups, with evidence that tailored programmes have greater impact than generic interventions. A range of modes of delivery (including telehealthcare) may be employed to suit preferences and context. The cost of providing self-management support is offset by a reduction in hospitalisations and unscheduled healthcare.

### Interpretation of findings

The literature on asthma self-management is particularly well developed and may thus be an exemplar for other LTCs [13, 14]. The 16 systematic reviews reporting effectiveness were typically large (five included data from >5000 participants [27, 30, 31, 41, 43]) and had consistently positive results, suggesting a mature evidence base, unlikely to be influenced by further trials. Outcomes in subgroups were more often the subject of the update RCTs as the field moves on from demonstrating overall effectiveness to investigating the impact in specific target groups [48–58, 60, 61, 72], demographic contexts [52–54, 66], or mode of delivery [54, 59, 62, 72].

Self-management support for asthma is a complex intervention and successful interventions were multi-component, including education, trigger avoidance, teaching self-monitoring, optimal treatment strategies, promotion of adherence and behaviour change techniques, many of which are common to self-management in other LTCs [6]. Appropriately in a variable condition [4], the hallmark of asthma self-management is the provision of an action plan with advice on recognising and responding to deterioration in control [4, 32]. People with asthma, however, have broader concerns as they accommodate the condition within their lives and the action plan needs to be embedded in support for ‘living with asthma’ [93].

Individuals with LTCs adjust medical regimes and self-management strategies to fit into their own lives and health beliefs [13]. Meta-reviews, for example in type 2 diabetes [94, 95], hypertension [96] and asthma [25], have emphasised the importance of culturally tailored interventions. Self-management support can be provided by many different professionals, often specialist nurses [38, 63] or LTC educators [25, 27, 95], but in some contexts the key personnel were community health workers [35, 97] or peer counsellors [30, 56, 66]. Traditionally education is delivered face-to-face, but increasingly technology-based interventions are being developed as alternatives [27, 28, 30, 42, 45, 47, 54, 57, 59, 62, 68].

Self-management support interventions are an integral component of high-quality care for people with LTCs [8–10]. Several of the systematic reviews demonstrated the synergy between self-management education and regular clinical review [31, 33, 36], and supported self-management is most effective when delivered within a proactive asthma management programme [33, 37, 65], or integrated within organisational routines [50, 52]. Only a minority of trials had follow-up periods over 12 months, and studies are needed to confirm long-term sustainability. Costs associated with self-management interventions are similar to usual care.

### Strengths and limitations

Meta-reviews have some intrinsic strengths and limitations. The methodology enables the efficient review of a large body of evidence and thus provision of a comprehensive overview to inform policy and practice. However, it relies on the quality of the included systematic reviews (e.g. comprehensive search strategies, accurate data extraction and synthesis). We used the validated R-AMSTAR instrument to assess the quality of included systematic reviews [17]. In contrast to GRADE [98] (now recommended by the Cochrane Handbook [15]), R-AMSTAR assesses the overall quality of the review, rather than assessing the quality of evidence individually for each outcome.

Re-synthesising materials that have already been synthesised risks further loss of detail and has the potential for erroneous assumptions, especially if the primary focus of the review did not directly align with the questions of the meta-review. Overlap between the RCTs included in the systematic reviews may result in undue emphasis on commonly cited papers.

Whilst some reviews and update RCTs directly compared interventions with or without specific components [23–25, 32, 36, 39, 43], or a specific mode of delivery [28, 29, 41, 45], often the different interventions were compared to usual care, allowing only indirect comparison [31, 33, 35, 37, 42, 46, 47]. A further limitation is that ‘usual care’ is rarely defined in RCTs [99], and the definition is even more unclear at meta-review level. Typically usual care is enhanced in the context of a trial, reducing the apparent impact of an intervention [100].

Systematic reviews are only as current as their most-recent search, and meta-reviews add an additional time delay. In the PRISMS meta-review we therefore not only updated our search for systematic reviews, but also searched for RCTs published after the date of the last search used by the included systematic reviews. In addition, prior to publication we undertook forward citation on all the included systematic reviews, which identified two recent systematic reviews and six RCTs [61–68]. None of these changed our conclusions, confirming the maturity of the evidence base.

The two reviewers who undertook the screening and data extraction were not working independently; however, both projects ensured all the reviewers were fully trained and instituted random checks at every stage. Restricting inclusion to reviews with extractable RCT data maintained the quality of evidence, but may have resulted in some lower-grade but useful evidence being rejected.

RECURSIVE was not restricted to formal cost-effectiveness studies – it had a broader focus and included studies reporting data on healthcare utilisation only, without a full effectiveness analysis including costs and quality of life. Some of the RCTs in the RECURSIVE meta-analysis used a more comprehensive definition of ‘total costs’ (e.g. based on societal perspective) compared to others; to account for this inconsistency, we also present the results on key sources of costs such as hospitalisation and A&E attendance rates.

The PRISMS and RECURSIVE teams worked independently, but met regularly throughout the studies to optimise synergies. A further strength was the multidisciplinary team, including backgrounds in public health, general practice, epidemiology and health psychology, enabling a balanced interpretation.

## Conclusions

Supported self-management for asthma can reduce unscheduled care, improve asthma control and quality of life, and does not lead to significant increases in total healthcare costs. Effective self-management should be tailored to cultural, clinical and demographic characteristics and is most effective when delivered in the context of proactive LTC management. Healthcare organisations should prioritise and promote the provision of supported self-management for people with asthma.

## Additional files

Additional file 1: Detailed search terms: PRISMS and RECURSIVE (all databases). (DOCX 88 kb)
Additional file 2: Dates of initial and update searches. (DOCX 21 kb)
Additional file 3: Detailed PICOS table and inclusion/exclusion criteria. (DOCX 22 kb)
Additional file 4: Quality assessment and weighting. (DOCX 43 kb)
Additional file 5: Characteristics of included studies and key outcomes. (DOCX 169 kb)

## Abbreviations

A&E: Accident and emergency
LTC: Long-term condition
RCTs: Randomised controlled trials
SMD: Standardised mean difference
