# Supplementary material for: Systematic meta-review of supported self-management for asthma: a healthcare perspective
Source: BMC Med. 2017 Mar 17;15:64. doi: 10.1186/s12916-017-0823-7 (PMC5356253; doi:10.1186/s12916-017-0823-7)
Supplement: Additional file 1: — Detailed search terms: PRISMS and RECURSIVE (all databases). (DOCX 88 kb) [file 12916_2017_823_MOESM1_ESM.docx]

Additional file 1. Search strategies

2.1 PRISMS Basic search strategy

General supported self-management (SMS) terms *or* LTC specific SMS terms

*AND*

LTC terms

*AND*

Quantitative or Qualitative review filter

*AND*

Apply limits to exclude articles that: study animals and do not include humans; were published before 1993; and are not published in English language.

All searches in [Title/Abstract]

2.2 PRISMS detailed search terms: a) general SMS terms

| **General Self Management Support terms: Medline, AMED, EMBASE, PsychINFO** | | | | |
| --- | --- | --- | --- | --- |
|  | **Medline** | **AMED** | **EMBASE** | **PsycINFO** |
|  | Exp Self care/ | Exp Self care/ | Exp Self care/ | Exp Self care skills/ |
|  | Exp Communication/ | Exp Education professional/ | Exp Health education/ | Exp Self management/ |
|  | Exp Professional Family Relations/ | Exp Education nonprofessional/ | Exp Patient education/ | Exp Health behavior/ |
|  | Exp Telephone/ | Exp Human activities/ | Exp Telehealth/ | Exp Self efficacy/ |
|  | Exp Professional Patient Relations/ | Exp Self concept/ | Exp Interpersonal communication/ | Exp Self help techniques/ |
|  | Exp Health education/ | Exp Self help groups/ | Exp Empowerment/ | Exp Coping behavior/ |
|  | Exp Attitude of health personnel/ | Exp Telemedicine/ | Exp Self concept/ | Exp Behavior modification/ |
|  | Exp Cellular phone/ | Exp Communication/ | Exp Patient participation/ | Exp Self monitoring/ |
|  | Exp Patient education as topic/ | Exp Rehabilitation/ |  | Exp Health knowledge/ |
|  | Exp Handheld computer/ | Exp Professional patient relations/ |  | Exp Health education/ |
|  | Exp Self efficacy/ | Exp Professional family relations/ |  | Exp Telemedicine/ |
|  | Exp Activities of Daily Living/ |  |  | Exp Client education/ |
|  | Exp Self help devices/ |  |  |  |
|  | Exp Community health services/ |  |  |  |
|  | Exp Rehabilitation/ |  |  |  |
|  | (Self ADJ2 (car* or manag* or help or administ* or monitor* or medicat*)) or self-car* or self-manag* or self-help or self-administ* or self-monitor* or self-medicat* or selfcar* or selfmanagement or selfhelp or selfadminist* or selfmonitor* or selfmedicat* | | | |
|  | SM or SMS | | | |
|  | Responsib* or Autonom* | | | |
|  | Manag* or copes or coping | | | |
|  | “Disease management” | | | |
|  | “expert patient” | | | |
|  | (professional or clinician) ADJ2 development | | | |
|  | Educat* or training or skill* or knowledge | | | |
|  | Confidence or self-efficacy | | | |
|  | (Access* or provi*) ADJ3 (information or records or results) | | | |
|  | Monitor* or self-monitor* or selfmonitor* | | | |
|  | ((patient or individual* or person* or client*) ADJ3 (remind* or feedback)) | | | |
|  | (Tele ADJ2 (health or medicine or care)) or tele-health or tele-medicine or tele-care or telehealth or telemedicine or telecare | | | |
|  | “Short message service” or SMS or “mobile phone” or “text message*” | | | |
|  | (home or environment* or living or assistive) ADJ2 (adaptation or modif* or equipment or technolog*) | | | |
|  | “Care plan*” | | | |
|  | “Action plan*” | | | |
|  | Hypno* ADJ1 (self or home) | | | |
|  | (cognitive or psychological or interpersonal or relaxation or biofeedback) ADJ3 (therap* or intervention* or program*) | | | |
|  | CBT | | | |
|  | Psychoeducation* | | | |
|  | (Peer or patient or emotional or social or psychosocial) ADJ1 (support or group) | | | |
|  | “Expert patient” | | | |
|  | Financial ADJ1 control | | | |
|  | “personal health budget*” | | | |
|  | (Financial or monetary or payment* or discount or service*) ADJ5 incentiv* | | | |

| **General Self Management Support terms: BNI, CINAHL** | | |
| --- | --- | --- |
|  | **BNI** | **CINAHL** |
|  | Exp Self care/ | Exp Self care/ |
|  | Exp Self medication/ | Exp Self concept/ |
|  | Exp Patients: education/ | Exp Patient education/ |
|  | Exp Personal care/ | Exp Health education/ |
|  | Exp Self help groups/ | Exp Attitude of Health Personnel/ |
|  | Exp Patients: empowerment/ | Exp Telehealth/ |
|  | Exp Interpersonal relations/ | Exp Communication skills/ |
|  | Exp Technology in health care/ | Exp Assistive technology devices/ |
|  | Exp Disabilities: aids and appliances/ | Exp Support groups/ |
|  | Exp Telemedicine/ | Exp Rehabilitation/ |
|  | Self ADJ2 (car* or manag* or help or admistrat* or monitor* or medicat*) | Self ADJ2 car* |
|  | or self-car* or self-manag* or self-help or self-adminisrat* or self-monitor* or self-medicat* | Self ADJ2 manag* |
|  | SM or SMS | Self ADJ2 help |
|  | Responsib* or Autonom* | Self ADJ2 administrat* |
|  | Manag* or copes or coping | Self ADJ2 monitor* |
|  | “Disease management” | Self ADJ2 medicat* |
|  | “expert patient” | self-car* |
|  | (professional or clinician) ADJ2 development | self-manag* |
|  | Educat* or training or skill* or knowledge | SM |
|  | Confidence or self-efficacy | SMS |
|  | (Access* or provi*) ADJ3 (information or records or results) | Autonom* |
|  | Monitor* or self-monitor* or selfmonitor* | Responsib* |
|  | ((patient or individual* or person* or client*) ADJ3 (remind* or feedback)) | Manag* |
|  | (Tele ADJ2 (health or medicine or care)) or tele-health or tele-medicine or tele-care or telehealth or telemedicine or telecare | copes |
|  | “Short message service” or SMS or “mobile phone” or “text message*” | coping |
|  | (home or environment* or living or assistive) ADJ2 (adaptation or modif* or equipment or technolog*) | “Disease management” |
|  | “Care plan*” | “expert patient” |
|  | “Action plan*” | Professional ADJ2 development |
|  | Hypno* ADJ1 (self or home) | Clinician ADJ2 development |
|  | (cognitive or psychological or interpersonal or relaxation or biofeedback) ADJ3 (therap* or intervention* or program*) | Educat* |
|  | CBT | knowledge |
|  | Psychoeducation* | skill* |
|  | (Peer or patient or emotional or social or psychosocial) ADJ1 (support or group) | training |
|  | “Expert patient” | self-efficacy |
|  | Financial ADJ1 control | Confidence |
|  | “personal health budget*” | Access* ADJ3 information |
|  | (Financial or monetary or payment* or discount or service*) ADJ5 incentiv* | Access* ADJ3 records |
|  |  | Access* ADJ3 results |
|  |  | Monitor* |
|  |  | Patient ADJ3 remind* |
|  |  | Patient ADJ3 feedback |
|  |  | Individual* ADJ3 remind |
|  |  | Individual* ADJ3 feedback |
|  |  | Tele ADJ2 health |
|  |  | Tele ADJ2 medicine |
|  |  | Tele ADJ2 care |
|  |  | “text message*” |
|  |  | Home ADJ2 adaptation |
|  |  | Home ADJ2 modif* |
|  |  | Assistive ADJ2 technolog* |
|  |  | “Care plan*” |
|  |  | “Action plan*” |
|  |  | Hypno* ADJ1 self |
|  |  | Cognitive ADJ3 therap* |
|  |  | Psychological ADJ3 intervention* |
|  |  | Relaxation ADJ3 program* |
|  |  | CBT |
|  |  | Psychoeducation* |
|  |  | Peer ADJ3 support |
|  |  | Patient ADJ3 group |
|  |  | “Expert patient” |
|  |  | Financial ADJ1 control |
|  |  | “personal health budget*” |
|  |  | Financial ADJ5 incentiv* |
|  |  | Monetary ADJ5 incentiv* |

b) LTC specific SMS terms

| **Asthma SMS terms: Medline, AMED, EMBASE, PsycINFO** | |
| --- | --- |
|  | Exercise or training or rehabilitati* |
|  | (Lifestyle or occupational) ADJ1 (intervention* or modification* or therapy) |
|  | Action plan* |
|  | (Reduce or remove) ADJ2 (dust or mite) |
|  | Buteyko or “breathing technique*” |
|  | (Smok* or nicotine or tobacco) ADJ3 (cessation or quit*) |

| **Asthma SMS terms: BNI & CINAHL** | | |
| --- | --- | --- |
|  | **BNI** | **CINAHL** |
|  | Exercise or training or rehabilitati* | Exercise |
|  | (Reduce or remove) ADJ2 (dust or mite) | training |
|  | Buteyko or “breathing technique” | rehabilitati* |
|  | (Lifestyle or occupational) ADJ1 (intervention* or modification* or therapy) | Lifestyle ADJ1 intervention* |
|  | (Smok* or nicotine or tobacco) ADJ3 (cessation or quit*) | Lifestyle ADJ1 modification* |
|  |  | Reduce ADJ2 dust |
|  |  | Buteyko |
|  |  | “breathing technique” |
|  |  | Smok* ADJ3 cessation |

c) LTC

(Note The initial PRISMS search included asthma and COPD in a single search)

| 1. **Asthma & COPD LTC terms: Medline, AMED, EMBASE, PsycINFO** | | | | |
| --- | --- | --- | --- | --- |
|  | **Medline** | **AMED** | **EMBASE** | **PsychINFO** |
|  | Exp Asthma/ | Asthma/ | Asthma/ | Asthma/ |
|  | Bronchial Spasm/ |  |  |  |
|  | Bronchoconstriction/ |  |  |  |
|  | Exp Pulmonary Disease, Chronic Obstructive/ | Exp Pulmonary Disease Chronic Obstructive/ | Chronic Obstructive Lung Disease/ | Exp Chronic Obstructive Pulmonary Disease/ |
|  | ((bronchial* or respiratory or airway* or lung*) ADJ3 (hypersensitive* or hyperreactiv* or allerg* or insufficiency)) | | | |
|  | Bronch* ADJ3 (constrict* or spas*) | | | |
|  | Asthma* | | | |
|  | Wheez* | | | |
|  | Bronchoconstrict* | | | |
|  | Antiasthma* | | | |
|  | Anti-asthma* | | | |
|  | “Respiratory sounds” | | | |
|  | “Bronchial hyperreactivity” | | | |
|  | Bronchospas* | | | |
|  | (Obstruct* ADJ3 (pulmonary or lung* or airway* or airflow* or bronch* or respirat*)) | | | |
|  | Chronic* ADJ3 bronchiti* | | | |
|  | “Chronic Obstructive Pulmonary Disease” | | | |
|  | “Chronic Obstructive Airways Disease” | | | |
|  | “Lung disease*” | | | |
|  | Emphysema* | | | |
|  | COPD or CAL or COAD or COLD or COBD or AECB | | | |

| 1. **Asthma & COPD LTC Terms: BNI, CINAHL** | | |
| --- | --- | --- |
|  | **BNI** | **CINAHL** |
|  | Asthma/ | Exp Asthma/ |
|  | COPD/ | Exp Pulmonary Disease, Chronic Obstructive/ |
|  | ((bronchial* or respiratory or airway* or lung*) ADJ3 (hypersensitiv* or hyperreactiv* or allerg* or insufficiency)) | Bronchial* ADJ3 hypersensitiv* |
|  | Bronch* ADJ3 (constrict* or spas*) | Bronchial* ADJ3 hyperreactiv* |
|  | Asthma* | Airway* ADJ3 hypersensitiv* |
|  | Wheez* | Respiratory ADJ3 insufficiency |
|  | Bronchoconstrict* | Airway* ADJ3 hyperreactiv* |
|  | Antiasthma* | Bronch* ADJ3 spas* |
|  | Anti-asthma* | Bronch* ADJ3 constrict* |
|  | “Respiratory sounds” | Asthma* |
|  | “Bronchial hyperreactivity” | Wheez* |
|  | Bronchospas* | Bronchoconstrict* |
|  | (Obstruct* ADJ3 (pulmonary or lung* or airway* or airflow* or bronch* or respirat*)) | Antiasthma* |
|  | Chronic* ADJ3 bronchiti* | Anti-asthma* |
|  | “Chronic Obstructive Pulmonary Disease” | “Respiratory sounds” |
|  | “Chronic Obstructive Pulmonary Disease” | “Bronchial hyperreactivity” |
|  | “Lung disease*” | Bronchospas* |
|  | Emphysema* | Obstruct* ADJ3 respirat* |
|  | COPD or CAL or COAD or COLD or COBD or AECB | Obstruct* ADJ3 lung* |
|  |  | Obstruct* ADJ3 airway* |
|  |  | Chronic* ADJ3 bronchiti* |
|  |  | “Chronic Obstructive Pulmonary Disease” |
|  |  | “Chronic Obstructive Airways Disease” |
|  |  | “Lung disease*” |
|  |  | Emphysema* |
|  |  | COPD |
|  |  | COAD |

d) Quantitative and Qualitative Review Filter

(The initial PRISMS search included quantitative and qualitative systematic reviews)

|  | **Quantitative and qualitative review filter: Medline, AMED, EMBASE, PsycINFO** | | | | | | |
| --- | --- | --- | --- | --- | --- | --- | --- |
|  | **Medline** | | **AMED** | | **EMBASE** | | **PsychINFO** |
|  | meta-analysis/ | | meta-analysis/ | | systematic review/ | | meta-analysis/ |
|  | meta analysis as topic/ | |  | | meta-analysis/ | | literature review/ |
|  | review literature as topic/ | |  | |  | |  |
|  | MEDLINE | | | | | | |
|  | (systematic review* or meta-analy* or metaanaly* or "research synthesis" or “literature review”) | | | | | | |
|  | systematic ADJ3 literature | | | | | | |
|  | data ADJ2 extract* | | | | | | |
|  | ((information or data) ADJ3 synthesis) | | | | | | |
|  | cochrane | | | | | | |
|  | (qualitative or narrative or thematic or evidence or realist or interpret* or induct* or refutational or framework or systematic or textual) adj2 (approach or review* or synthes* or meta-summary or “meta summary” or summary) | | | | | | |
|  | Meta adj1 (summary or narrative or synthesis or ethnograph* or study or data or interpretation or aggregation or needs-assessment or ‘needs assessment’) | | | | | | |
|  | meta-summary or meta-narrative or meta-synthesis or meta-ethnograph* or meta-study or meta-data-analysis or meta-data-synthesis or meta-interpretation or meta-aggregation | | | | | | |
|  | ‘reciprocal translational analysis’ | | | | | | |
|  | ‘lines-of-arg?ment synthesis’ or ‘lines of arg?ment synthesis’ | | | | | | |
|  | ‘LOA synthesis’ | | | | | | |
|  | ‘grounded formal theory’ | | | | | | |
|  | ‘grounded theory synthesis’ | | | | | | |
|  | ecological adj2 (triangulation or sentence or synthesis) | | | | | | |
|  | Phenomenography | | | | | | |
|  | ((mixed or multi* or cross) adj1 (method* or design* or research or strategy)) adj2 (synthesis or review) | | | | | | |
|  | (mixed-method* or multi-method* or mixed-design or multi-design or multiple-methods or multi-strategy or cross-design) adj2 (synthesis or review) | | | | | | |
|  | Bayesian adj1 (meta-analysis or ‘meta analysis’) | | | | | | |
|  | ‘case survey’ | | | | | | |
|  | “qualitative comparative analysis” | | | | | | |
|  | Or/ 1-25 | | | | | | |
|  | letter.pt. | Letter.pt | | letter.pt | | - | |
|  | comment.pt. | Comment.pt or commentary.pt | | - | | - | |
|  | editorial.pt. | editorial.pt. | | editorial.pt | | - | |
|  | Or/26-28 | | | | | | |
|  | 25 not 29 | | | | | | |

**2.3 RECURSIVE Detailed search strategy**

**a) CENTRAL – via Cochrane Library**

| **ID** | **Search** |
| --- | --- |
| #1 | (self NEXT administer*) in Trials |
| #2 | [MeSH descriptor Self Administration, this term only](http://onlinelibrary.wiley.com/o/cochrane/searchHistory?mode=runquery&qnum=2) |
| #3 | [MeSH descriptor Self Care, this term only](http://onlinelibrary.wiley.com/o/cochrane/searchHistory?mode=runquery&qnum=3) |
| #4 | ["self care" or (selfcare) or (self NEXT manage*) or (selfmonitor*) or (self NEXT monitor*) in Trials](http://onlinelibrary.wiley.com/o/cochrane/searchHistory?mode=runquery&qnum=4) |
| #5 | [(selfhelp) or "self help" or (self NEXT diagnos*) or (selfdiagnos*) in Trials](http://onlinelibrary.wiley.com/o/cochrane/searchHistory?mode=runquery&qnum=5) |
| #6 | [(self NEXT assess*) or (selfassess*) in Trials](http://onlinelibrary.wiley.com/o/cochrane/searchHistory?mode=runquery&qnum=6) |
| #7 | [MeSH descriptor Blood Glucose Self-Monitoring, this term only](http://onlinelibrary.wiley.com/o/cochrane/searchHistory?mode=runquery&qnum=7) |
| #8 | ["self inititiated intervention" in Trials](http://onlinelibrary.wiley.com/o/cochrane/searchHistory?mode=runquery&qnum=8) |
| #9 | [(self NEXT initiated NEXT intervent*) in Trials](http://onlinelibrary.wiley.com/o/cochrane/searchHistory?mode=runquery&qnum=9) |
| #10 | [MeSH descriptor Self Efficacy, this term only](http://onlinelibrary.wiley.com/o/cochrane/searchHistory?mode=runquery&qnum=10) |
| #11 | [MeSH descriptor Self Medication explode all trees](http://onlinelibrary.wiley.com/o/cochrane/searchHistory?mode=runquery&qnum=11) |
| #12 | ["self efficacy" or (pharmacist* or pharmacy or pharmacies) NEAR/2 support* in Trials](http://onlinelibrary.wiley.com/o/cochrane/searchHistory?mode=runquery&qnum=12) |
| #13 | [(pharmacist* or pharmacy or pharmacies) NEAR/2 assist* or (pharmacist* or pharmacy or pharmacies) NEAR/2 (advice or advis* or inform*) or "pharmaceutical care" in Trials](http://onlinelibrary.wiley.com/o/cochrane/searchHistory?mode=runquery&qnum=13) |
| #14 | [(self NEXT medicat*) or (selfmedicat*) or (self NEXT remed*) or (selfremed*) in Trials](http://onlinelibrary.wiley.com/o/cochrane/searchHistory?mode=runquery&qnum=14) |
| #15 | [(self NEXT treat*) or (selftreat*) or "self cure" or (selfcure) in Trials](http://onlinelibrary.wiley.com/o/cochrane/searchHistory?mode=runquery&qnum=15) |
| #16 | [MeSH descriptor Self-Help Groups, this term only](http://onlinelibrary.wiley.com/o/cochrane/searchHistory?mode=runquery&qnum=16) |
| #17 | [MeSH descriptor Social Support explode all trees](http://onlinelibrary.wiley.com/o/cochrane/searchHistory?mode=runquery&qnum=17) |
| #18 | [(social NEXT support*) in Trials](http://onlinelibrary.wiley.com/o/cochrane/searchHistory?mode=runquery&qnum=18) |
| #19 | [(group NEAR/1 (support* or advice or advis* or monitor* or intervention* or train* or instruction or consult* or assist* or education or information)) in Trials](http://onlinelibrary.wiley.com/o/cochrane/searchHistory?mode=runquery&qnum=19) |
| #20 | [(peer NEAR/1 (support* or advice or advis* or monitor* or intervention* or train* or instruction or consult* or assist* or education or information)) in Trials](http://onlinelibrary.wiley.com/o/cochrane/searchHistory?mode=runquery&qnum=20) |
| #21 | [(expert NEXT patient*) or "psychosocial support" or (befriend*) or (health NEXT trainer*) in Trials](http://onlinelibrary.wiley.com/o/cochrane/searchHistory?mode=runquery&qnum=21) |
| #22 | [MeSH descriptor Telemedicine, this term only](http://onlinelibrary.wiley.com/o/cochrane/searchHistory?mode=runquery&qnum=22) |
| #23 | [(telemedicine) or (telecare) or (telenursing) or (telemonitor*) or (telehealth) in Trials](http://onlinelibrary.wiley.com/o/cochrane/searchHistory?mode=runquery&qnum=23) |
| #24 | [MeSH descriptor Remote Consultation, this term only](http://onlinelibrary.wiley.com/o/cochrane/searchHistory?mode=runquery&qnum=24) |
| #25 | [(telephon* or remote or phone) NEAR/2 (follow* or support or consult* or advice or advis* or intervention or train* or instruction or assist* or educate or education or information or monitor*) in Trials](http://onlinelibrary.wiley.com/o/cochrane/searchHistory?mode=runquery&qnum=25) |
| #26 | ["case management" or (action NEXT plan*) or (management NEXT plan*) or (management NEXT program*) or (care NEXT plan*) in Trials](http://onlinelibrary.wiley.com/o/cochrane/searchHistory?mode=runquery&qnum=26) |
| #27 | [(nurse NEAR/2 educator*) in Trials](http://onlinelibrary.wiley.com/o/cochrane/searchHistory?mode=runquery&qnum=27) |
| #28 | ["patient education" in Trials](http://onlinelibrary.wiley.com/o/cochrane/searchHistory?mode=runquery&qnum=28) |
| #29 | [MeSH descriptor Patient Education as Topic, this term only](http://onlinelibrary.wiley.com/o/cochrane/searchHistory?mode=runquery&qnum=29) |
| #30 | [MeSH descriptor Case Management, this term only](http://onlinelibrary.wiley.com/o/cochrane/searchHistory?mode=runquery&qnum=30) |
| #31 | [(patient NEAR/2 (education or advice or advis* or instruct* or educate or train*)) in Trials](http://onlinelibrary.wiley.com/o/cochrane/searchHistory?mode=runquery&qnum=31) |
| #32 | ["consumer health information" or "patient information" in Trials](http://onlinelibrary.wiley.com/o/cochrane/searchHistory?mode=runquery&qnum=32) |
| #33 | [(financial or monetary or money) NEAR/2 (incentive* or competition* or contest* or lotter* or reward* or prize*) in Trials](http://onlinelibrary.wiley.com/o/cochrane/searchHistory?mode=runquery&qnum=33) |
| #34 | [(contingent NEXT payment*) or (deposit NEXT contract*) or (decision NEAR/2 support*) or (decision NEAR/2 aid*) or (shared NEAR/2 decision*) in Trials](http://onlinelibrary.wiley.com/o/cochrane/searchHistory?mode=runquery&qnum=34) |
| #35 | [MeSH descriptor Decision Making, this term only](http://onlinelibrary.wiley.com/o/cochrane/searchHistory?mode=runquery&qnum=35) |
| #36 | [(#1 OR #2 OR #3 OR #4 OR #5 OR #6 OR #7 OR #8 OR #9 OR #10 OR #11 OR #12 OR #13 OR #14 OR #15 OR #16 OR #17 OR #18 OR #19 OR #20 OR #21 OR #22 OR #23 OR #24 OR #25 OR #26 OR #27 OR #28 OR #29 OR #30 OR #31 OR #32 OR #33 OR #34 OR #35)](http://onlinelibrary.wiley.com/o/cochrane/searchHistory?mode=runquery&qnum=36) |
| #37 | [MeSH descriptor Hospitalization explode all trees](http://onlinelibrary.wiley.com/o/cochrane/searchHistory?mode=runquery&qnum=37) |
| #38 | [MeSH descriptor Health Resources, this term only](http://onlinelibrary.wiley.com/o/cochrane/searchHistory?mode=runquery&qnum=38) |
| #39 | [(length NEAR/2 stay) or (duration NEAR/2 stay) or (hospital NEAR/1 (visit* or contact* or attendance* or admission* or episode*)) or (time NEAR/2 discharge) or (hospital NEXT day*) in Trials](http://onlinelibrary.wiley.com/o/cochrane/searchHistory?mode=runquery&qnum=39) |
| #40 | [(patient* or inpatient* or in-patient*) NEAR/1 (cost* or stay) or (number NEAR/2 (nights or days)) in Trials](http://onlinelibrary.wiley.com/o/cochrane/searchHistory?mode=runquery&qnum=40) |
| #41 | ["primary care" NEAR/2 (visit* or contact* or attendance* or admission* or episode*) or (surgery NEAR/2 (visit* or contact* or attendance* or admission* or episode*)) in Trials](http://onlinelibrary.wiley.com/o/cochrane/searchHistory?mode=runquery&qnum=41) |
| #42 | [(clinic or surgery or hospital or "accident and emergency") NEAR/2 (work-flow or "work flow") in Trials](http://onlinelibrary.wiley.com/o/cochrane/searchHistory?mode=runquery&qnum=42) |
| #43 | [(consultation* NEAR/2 (time or length)) or (hospitalization* or hospitalisation* or rehospitalization* or rehospitalisation* or re-hospitalization* or re-hospitalisation*) or "hospital costs" in Trials](http://onlinelibrary.wiley.com/o/cochrane/searchHistory?mode=runquery&qnum=43) |
| #44 | [(#37 OR #38 OR #39 OR #40 OR #41 OR #42 OR #43)](http://onlinelibrary.wiley.com/o/cochrane/searchHistory?mode=runquery&qnum=44) |
| #45 | [(#36 AND #44)](http://onlinelibrary.wiley.com/o/cochrane/searchHistory?mode=runquery&qnum=45) |
| #46 | [MeSH descriptor Economics, this term only](http://onlinelibrary.wiley.com/o/cochrane/searchHistory?mode=runquery&qnum=46) |
| #47 | [MeSH descriptor Costs and Cost Analysis explode all trees](http://onlinelibrary.wiley.com/o/cochrane/searchHistory?mode=runquery&qnum=47) |
| #48 | [MeSH descriptor Value of Life, this term only](http://onlinelibrary.wiley.com/o/cochrane/searchHistory?mode=runquery&qnum=48) |
| #49 | [MeSH descriptor Economics, Dental, this term only](http://onlinelibrary.wiley.com/o/cochrane/searchHistory?mode=runquery&qnum=49) |
| #50 | [MeSH descriptor Economics, Hospital explode all trees](http://onlinelibrary.wiley.com/o/cochrane/searchHistory?mode=runquery&qnum=50) |
| #51 | [MeSH descriptor Economics, Medical, this term only](http://onlinelibrary.wiley.com/o/cochrane/searchHistory?mode=runquery&qnum=51) |
| #52 | [MeSH descriptor Economics, Nursing, this term only](http://onlinelibrary.wiley.com/o/cochrane/searchHistory?mode=runquery&qnum=52) |
| #53 | [MeSH descriptor Economics, Pharmaceutical, this term only](http://onlinelibrary.wiley.com/o/cochrane/searchHistory?mode=runquery&qnum=53) |
| #54 | [(#46 OR #47 OR #48 OR #49 OR #50 OR #51 OR #52 OR #53)](http://onlinelibrary.wiley.com/o/cochrane/searchHistory?mode=runquery&qnum=54) |
| #55 | [econom* or cost or costs or costly or costing or price or prices or pricing or pharmacoeconomic* in Trials](http://onlinelibrary.wiley.com/o/cochrane/searchHistory?mode=runquery&qnum=55) |
| #56 | [expenditure NOT energy in Trials](http://onlinelibrary.wiley.com/o/cochrane/searchHistory?mode=runquery&qnum=56) |
| #57 | [value NEAR/2 money in Trials](http://onlinelibrary.wiley.com/o/cochrane/searchHistory?mode=runquery&qnum=57) |
| #58 | [budget* in Trials](http://onlinelibrary.wiley.com/o/cochrane/searchHistory?mode=runquery&qnum=58) |
| #59 | [(#55 OR #56 OR #57 OR #58)](http://onlinelibrary.wiley.com/o/cochrane/searchHistory?mode=runquery&qnum=59) |
| #60 | [(#54 OR #59)](http://onlinelibrary.wiley.com/o/cochrane/searchHistory?mode=runquery&qnum=60) |
| #61 | [metabolic NEAR/1 cost in Trials](http://onlinelibrary.wiley.com/o/cochrane/searchHistory?mode=runquery&qnum=61) |
| #62 | [(energy or oxygen) NEAR/1 cost in Trials](http://onlinelibrary.wiley.com/o/cochrane/searchHistory?mode=runquery&qnum=62) |
| #63 | [(#61 OR #62)](http://onlinelibrary.wiley.com/o/cochrane/searchHistory?mode=runquery&qnum=63) |
| #64 | [(#60 AND NOT #63)](http://onlinelibrary.wiley.com/o/cochrane/searchHistory?mode=runquery&qnum=64) |
| #65 | [(#45 AND #60)](http://onlinelibrary.wiley.com/o/cochrane/searchHistory?mode=runquery&qnum=65) |

Note: MeSH searches cannot be limited to CENTRAL, so some results apply the whole library

**b) CINAHL via EBSCO**

| **ID** | **Search** |
| --- | --- |
| 1 | TI ( econom* or cost or costs or costly or costing or price or prices or pricing or pharmacoeconomic* ) OR AB ( econom* or cost or costs or costly or costing or price or prices or pricing or pharmacoeconomic* ) |
| 2 | TI ( expenditure* not energy ) OR AB ( expenditure* not energy ) |
| 3 | TI value N1 money OR AB value N1 money |
| 4 | TI budget* OR AB budget* |
| 5 | S1 or S2 or S3 or S4 |
| 6 | TI metabolic N1 cost OR AB metabolic N1 cost |
| 7 | TI ( (energy or oxygen) N1 cost ) OR AB ( (energy or oxygen) N1 cost ) |
| 8 | S6 or S7 |
| 9 | S5 not S8 |
| 10 | (MH "Economics") OR (MH "Costs and Cost Analysis+") OR (MH "Economic Value of Life") OR (MH "Economics, Dental") OR (MH "Economics, Pharmaceutical") OR (MH "Health Resource Allocation") |
| 11 | (MH "Hospitalization") OR (MH "Patient Admission") OR (MH "Length of Stay") |
| 12 | (MH "Readmission") |
| 13 | (MH "Health Resource Utilization") |
| 14 | TI length N2 stay OR AB length N2 stay |
| 15 | TI duration N2 stay OR AB duration N2 stay |
| 16 | TI ( hospital N1 (visit* or contact* or attendance* or admission* or episode*) ) OR AB ( hospital N1 (visit* or contact* or attendance* or admission* or episode*) ) |
| 17 | TI hospital costs OR AB hospital costs |
| 18 | TI time N2 discharge OR AB time N2 discharge |
| 19 | TI hospital day* OR AB hospital day* |
| 20 | TI ( (patient* or inpatient* or in-patient*) N1 (cost* or stay) ) OR AB ( (patient* or inpatient* or in-patient*) N1 (cost* or stay) ) |
| 21 | TI ( (number N2 (nights or days) ) OR AB ( (number N2 (nights or days) ) |
| 22 | TI ( "primary care" N1 (visit* or contact* or attendance* or admission* or episode*) ) OR AB ( "primary care" N1 (visit* or contact* or attendance* or admission* or episode*) ) |
| 23 | TI ( surgery N1 (visit* or contact* or attendance* or admission* or episode*) ) OR AB ( surgery N1 (visit* or contact* or attendance* or admission* or episode*) ) |
| 24 | TI ( consultation* N2 (time or length) ) OR AB ( consultation* N2 (time or length) ) |
| 25 | TI ( hospitalization* or hospitalisation* or rehospitalisation* or rehospitalisation* or re-hospitalization* or re-hospitalisation* ) OR AB ( hospitalization* or hospitalisation* or rehospitalisation* or rehospitalisation* or re-hospitalization* or re-hospitalisation* ) |
| 26 | S9 or S10 or S11 or S12 or S13 or S14 or S15 or S16 or S17 or S18 |
| 27 | S19 or S20 or S22 or S23 or S24 or S25 |
| 28 | S26 or S27 |
| 29 | (MH "Blood Glucose Self-Monitoring") |
| 30 | (MH "Support Groups") |
| 31 | (MH "Self Administration") |
| 32 | (MH "Self Medication") |
| 33 | (MH "Self Diagnosis") |
| 34 | (MH "Access to Information+") |
| 35 | (MH "Patient Education") |
| 36 | (MH "Telemedicine") OR (MH "Telehealth") OR (MH "Telenursing") |
| 37 | (MH "Patient Care Plans") |
| 38 | TI "self care" OR AB "self care" OR TI selfcare OR AB selfcare |
| 39 | TI "self manag*" OR AB "self manag*" OR TI "selfmanag*" OR AB "selfmanag*" |
| 40 | TI "self monitor*" OR AB "self monitor*" OR TI "selfmonitor*" OR AB "selfmonitor*" |
| 41 | TI "self help" OR AB "self help*" OR TI "selfhelp*" OR AB "selfhelp*" |
| 42 | TI "self diagnos*" OR AB "self diagnos*" OR TI "selfdiagnos*" OR AB "selfdiagnos*" |
| 43 | TI "self assess*" OR AB "self assess*" OR TI "selfassess*" OR AB "selfassess*" |
| 44 | TI "Self initiated intervention*" OR AB "Self initiated intervention*" |
| 45 | TI "Self efficacy" OR AB "Self efficacy" |
| 46 | TI pharmacist* N2 support* OR AB pharmacist* N2 support* OR TI pharmacy N2 support* OR AB pharmacy N2 support* OR TI pharmacies N2 support* OR AB pharmacies N2 support* |
| 47 | TI pharmacist* N2 assist* OR AB pharmacist* N2 assist* OR TI pharmacy N2 assist* OR AB pharmacy N2 assist* OR TI pharmacies N2 assist* OR AB pharmacies N2 assist* |
| 48 | TI pharmacist* N2 advice OR AB pharmacist* N2 advice OR TI pharmacy N2 advice OR AB pharmacy N2 advice OR TI pharmacies N2 advice OR AB pharmacies N2 advice |
| 49 | TI pharmacist* N2 advis* OR AB pharmacist* N2 advis* OR TI pharmacy N2 advis* OR AB pharmacy N2 advis* OR TI pharmacies N2 advis* OR AB pharmacies N2 advis* |
| 50 | TI pharmacist* N2 inform* OR AB pharmacist* N2 inform* OR TI pharmacy N2 inform* OR AB pharmacy N2 inform* OR TI pharmacies N2 inform* OR AB pharmacies N2 inform* |
| 51 | TI "pharmaceutical care" OR AB "pharmaceutical care" |
| 52 | TI ( "self medicat*" or selfmedicat* or "self remed*" or selfremed* ) OR AB ( "self medicat*" or selfmedicat* or "self remed*" or selfremed* ) |
| 53 | TI ( "self treat*" or selftreat* or "self cure" or selfcure ) OR AB ( "self treat*" or selftreat* or "self cure" or selfcure ) |
| 54 | TI "Social support*" OR AB "Social support*" |
| 55 | TI ( group N1 (support* or advice or advis* or monitor* or intervention* or train* or instruction or consult* or assist* or education or educate or information) ) OR AB ( group N1 (support* or advice or advis* or monitor* or intervention* or train* or instruction or consult* or assist* or education or educate or information) ) |
| 56 | TI ( peer N1 (support* or advice or advis* or monitor* or intervention* or train* or instruction or consult* or assist* or education or educate or information) ) OR AB ( peer N1 (support* or advice or advis* or monitor* or intervention* or train* or instruction or consult* or assist* or education or educate or information) ) |
| 57 | TI "expert patient*" OR AB "expert patient*" |
| 58 | TI "Psychosocial support" OR AB "Psychosocial support" |
| 59 | TI Befriend* OR AB Befriend* |
| 60 | TI "Health trainer*" OR AB "Health trainer*" |
| 61 | TI telemedicine OR AB telemedicine |
| 62 | TI telecare OR AB telecare |
| 63 | TI telenursing OR AB telenursing |
| 64 | TI telemonitor* OR AB telemonitor* |
| 65 | TI telehealth OR AB telehealth |
| 66 | TI ( telephon* N2 (follow* or support or consult* or advice or advis* or intervention* or train* or instruction or assis* or educate or education or information or monitor*) ) OR AB ( telephon* N2 (follow* or support or consult* or advice or advis* or intervention* or train* or instruction or assis* or educate or education or information or monitor*) ) OR TI ( remote N2 (follow* or support or consult* or advice or advis* or intervention* or train* or instruction or assis* or educate or education or information or monitor*) ) OR AB ( remote N2 (follow* or support or consult* or advice or advis* or intervention* or train* or instruction or assis* or educate or education or information or monitor*) ) OR TI ( phone N2 (follow* or support or consult* or advice or advis* or intervention* or train* or instruction or assis* or educate or education or information or monitor*) ) OR AB ( phone N2 (follow* or support or consult* or advice or advis* or intervention* or train* or instruction or assis* or educate or education or information or monitor*) ) |
| 67 | TI "case management" OR AB "case management" |
| 68 | TI "Action plan*" OR AB "Action plan*" |
| 69 | TI "Management plan*" OR AB "Management plan*" |
| 70 | TI "care plan*" OR AB "care plan*" |
| 71 | TI "nurse adj2 educator*" OR AB "nurse adj2 educator*" |
| 72 | TI ( patient N2 (education or advice or advis* or instruct* or educate or train*) ) OR AB ( patient N2 (education or advice or advis* or instruct* or educate or train*) ) |
| 73 | TI "Consumer health information" OR AB "Consumer health information" |
| 74 | TI "patient information" OR AB "patient information" |
| 75 | TI ( financial N2 (incentive* or competition* or contest* or lotter* or reward* or prize*) ) OR AB ( financial N2 (incentive* or competition* or contest* or lotter* or reward* or prize*) ) OR TI ( monetary N2 (incentive* or competition* or contest* or lotter* or reward* or prize*) ) OR AB ( monetary N2 (incentive* or competition* or contest* or lotter* or reward* or prize*) ) OR TI ( money N2 (incentive* or competition* or contest* or lotter* or reward* or prize*) ) OR AB ( money N2 (incentive* or competition* or contest* or lotter* or reward* or prize*) ) |
| 76 | TI ( financial N2 (incentive* or competition* or contest* or lotter* or reward* or prize*) ) OR AB ( financial N2 (incentive* or competition* or contest* or lotter* or reward* or prize*) ) OR TI ( monetary N2 (incentive* or competition* or contest* or lotter* or reward* or prize*) ) OR AB ( monetary N2 (incentive* or competition* or contest* or lotter* or reward* or prize*) ) OR TI ( money N2 (incentive* or competition* or contest* or lotter* or reward* or prize*) ) OR AB ( money N2 (incentive* or competition* or contest* or lotter* or reward* or prize*) ) |
| 77 | TI ( "contingent payment*" or "deposit contract*" ) OR AB ( "contingent payment*" or "deposit contract*" ) |
| 78 | TI decision* N2 support* OR AB decision* N2 support* |
| 79 | TI decision* N2 aid* OR AB decision* N2 aid* |
| 80 | TI shared N2 decision* OR AB shared N2 decision* |
| 81 | S29 or S30 or S31 or S32 or S33 or S34 or S35 or S36 or S37 or S38 or S39 or S40 or S41 or S42 or S43 or S44 or S45 or S46 or S47 or S48 or S49 or S50 or S51 or S52 or S53 or S54 or S55 or S56 or S57 or S58 or S59 or S60 or S61 or S62 or S63 or S64 or S65 or S66 or S67 or S68 or S69 or S70 or S71 or S72 or S73 or S74 or S75 or S76 or S77 or S78 or S79 or S80 |
| 82 | S28 and S81 |
| 83 | (MH "Clinical Trials+") |
| 84 | PT Clinical trial |
| 85 | TX clinic* n1 trial* |
| 86 | TX ( (singl* n1 blind*) or (singl* n1 mask*) or (doubl* n1 blind*) or (doubl* n1 mask*) or (tripl* n1 blind*) or (tripl* n1 mask*) or (trebl* n1 blind*) or (trebl* n1 mask*) ) |
| 87 | TX "randomi* control* trial*" |
| 88 | (MH "Random Assignment") |
| 89 | TX "random* allocat*" |
| 90 | TX placebo* |
| 91 | (MH "Placebos") |
| 92 | (MH "Quantitative Studies") |
| 93 | TX "allocat* random*" |
| 94 | S83 or S84 or S85 or S86 or S87 or S88 or S89 or S90 or S91 or S92 or S93 |
| 95 | S82 and S94 |

**c) Econlit (1961 to April 2012) Via OvidSP**

| **ID** | **Search** |
| --- | --- |
| 1 | ((self administer$ adj2 questionnaire$) or (self administer$ adj2 survey$) or (selfadminister$ adj2 interview$)).ti,ab. (63) |
| 2 | self administer$.ti,ab. (80) |
| 3 | 2 not 1 (17) |
| 4 | (self care or selfcare).ti,ab. (24) |
| 5 | (self manag$ or selfmanag$).ti,ab. (374) |
| 6 | (self monitor$ or selfmonitor$).ti,ab. (33) |
| 7 | (self help or selfhelp).ti,ab. (292) |
| 8 | (self diagnos$ or selfdiagnos$ or self assess$ or selfassess$).ti,ab. (357) |
| 9 | Self initiated intervention$.ti,ab. (0) |
| 10 | Self efficacy.ti,ab. (138) |
| 11 | ((pharmacist$ or pharmacy or pharmacies) adj2 support$).ti,ab. (2) |
| 12 | ((pharmacist$ or pharmacy or pharmacies) adj2 assist$).ti,ab. (3) |
| 13 | ((pharmacist or pharmacy or pharmacies) adj2 (advice or advis$ or inform$)).ti,ab. (2) |
| 14 | pharmaceutical care.ti,ab. (20) |
| 15 | (self medicat$ or selfmedicat$ or self remed$ or selfremed$).ti,ab. (19) |
| 16 | (self treat$ or selftreat$ or self cure or selfcure).ti,ab. (7) |
| 17 | Social support$.ti,ab. (223) |
| 18 | (group adj1 (support$ or advice or advis$ or monitor$ or intervention$ or train$ or instruction or consult$ or assist$ or education or educate or information)).ti,ab. (240) |
| 19 | (peer adj1 (support$ or advice or advis$ or monitor$ or intervention$ or train$ or instruction or consult$ or assist$ or education or educate or information)).ti,ab. (86) |
| 20 | expert patient$.ti,ab. (1) |
| 21 | Psychosocial support.ti,ab. (3) |
| 22 | Befriend$.ti,ab. (5) |
| 23 | Health trainer$.ti,ab. (0) |
| 24 | telemedicine.ti,ab. (18) |
| 25 | telecare.ti,ab. (2) |
| 26 | telenursing.ti,ab. (0) |
| 27 | telemonitor$.ti,ab. (3) |
| 28 | telehealth.ti,ab. (3) |
| 29 | ((telephon$ or remote or phone) adj2 (follow$ or support or consult$ or advice or advis$ or intervention$ or train$ or instruction or assis$ or educate or education or information or monitor$)).ti,ab. (51) |
| 30 | case management.ti,ab. (68) |
| 31 | Action plan$.ti,ab. (372) |
| 32 | Management plan$.ti,ab. (220) |
| 33 | Management program$.ti,ab. (319) |
| 34 | care plan$.ti,ab. (146) |
| 35 | (nurse adj2 educator$).ti,ab. (0) |
| 36 | patient education.ti,ab. (4) |
| 37 | (patient adj2 (education or advice or advis$ or instruct$ or educate or train$)).ti,ab. (8) |
| 38 | Consumer health information.ti,ab. (8) |
| 39 | patient informat$.ti,ab. (6) |
| 40 | ((financial or monetary or money) adj2 (incentive$ or competition$ or contest$ or lotter$ or reward$ or prize$)).ti,ab. (1622) |
| 41 | (contingent payment$ or deposit contract$).ti,ab. (100) |
| 42 | (decision$ adj2 support$).ti,ab. (1016) |
| 43 | (decision$ adj2 aid$).ti,ab. (275) |
| 44 | (shared adj2 decision$).ti,ab. (20) |
| 45 | or/3-44 (5975) |
| 46 | trial$.ti,ab. (1962) |
| 47 | random$.ti,ab. (14667) |
| 48 | placebo$.ti,ab. (105) |
| 49 | 46 or 47 or 48 (16281) |
| 50 | 45 and 49 (226) |

**d) Embase <1974 to 2012 May 17> Via OvidSP**

| **ID** | **Search** |
| --- | --- |
| 1 | ((self administer$ adj2 questionnaire$) or (self administer$ adj2 survey$) or (selfadminister$ adj2 interview$)).ti,ab. (14115) |
| 2 | self administer$.ti,ab. (23363) |
| 3 | 2 not 1 (9253) |
| 4 | drug self administration/ (6495) |
| 5 | self care/ (24244) |
| 6 | (self care or selfcare).ti,ab. (10752) |
| 7 | (self manag$ or selfmanag$).ti,ab. (8754) |
| 8 | (self monitor$ or selfmonitor$).ti,ab. (5119) |
| 9 | (self help or selfhelp).ti,ab. (5355) |
| 10 | (self diagnos$ or selfdiagnos$ or self assess$ or selfassess$).ti,ab. (11236) |
| 11 | self help/ (10320) |
| 12 | Self initiated intervention$.ti,ab. (0) |
| 13 | Self efficacy.ti,ab. (11617) |
| 14 | self medication/ (7565) |
| 15 | ((pharmacist$ or pharmacy or pharmacies) adj2 support$).ti,ab. (531) |
| 16 | ((pharmacist$ or pharmacy or pharmacies) adj2 assist$).ti,ab. (559) |
| 17 | ((pharmacist or pharmacy or pharmacies) adj2 (advice or advis$ or inform$)).ti,ab. (705) |
| 18 | pharmaceutical care.ti,ab. (2633) |
| 19 | (self medicat$ or selfmedicat$ or self remed$ or selfremed$).ti,ab. (3548) |
| 20 | (self treat$ or selftreat$ or self cure or selfcure).ti,ab. (1673) |
| 21 | social support/ (48288) |
| 22 | Social support$.ti,ab. (22290) |
| 23 | (group adj1 (support$ or advice or advis$ or monitor$ or intervention$ or train$ or instruction or consult$ or assist$ or education or educate or information)).ti,ab. (27340) |
| 24 | 24 (peer adj1 (support$ or advice or advis$ or monitor$ or intervention$ or train$ or instruction or consult$ or assist$ or education or educate or information)).ti,ab. (2699) |
| 25 | expert patient$.ti,ab. (181) |
| 26 | Psychosocial support.ti,ab. (2046) |
| 27 | Befriend$.ti,ab. (157) |
| 28 | Health trainer$.ti,ab. (33) |
| 29 | telemedicine/ or telemonitoring/ (8783) |
| 30 | telemedicine.ti,ab. (6025) |
| 31 | telecare.ti,ab. (334) |
| 32 | telenursing.ti,ab. (73) |
| 33 | telemonitor$.ti,ab. (629) |
| 34 | telehealth.ti,ab. (1305) |
| 35 | teleconsultation/ or telehealth/ (4351) |
| 36 | (telephon$ or remote or phone) adj2 (follow$ or support or consult$ or advice or advis$ or intervention$ or train$ or instruction or assis$ or educate or education or information or monitor$)).ti,ab. (9507) |
| 37 | Case Management/ (6305) |
| 38 | case management.ti,ab. (7581) |
| 39 | Action plan$.ti,ab. (3954) |
| 40 | Management plan$.ti,ab. (4582) |
| 41 | Management program$.ti,ab. (9326) |
| 42 | care plan$.ti,ab. (9014) |
| 43 | (nurse adj2 educator$).ti,ab. (2285) |
| 44 | patient education.ti,ab. (12644) |
| 45 | Patient Education/ (78754) |
| 46 | (patient adj2 (education or advice or advis$ or instruct$ or educate or train$)).ti,ab. (17190) |
| 47 | Consumer health information.ti,ab. (217) |
| 48 | patient informat$.ti,ab. (5706) |
| 49 | patient information/ (16505) |
| 50 | ((financial or monetary or money) adj2 (incentive$ or competition$ or contest$ or lotter$ or reward$ or prize$)).ti,ab. (3925) |
| 51 | (contingent payment$ or deposit contract$).ti,ab. (27) |
| 52 | Decision Making/ (118692) |
| 53 | (decision$ adj2 support$).ti,ab. (9560) |
| 54 | (decision$ adj2 aid$).ti,ab. (2972) |
| 55 | (shared adj2 decision$).ti,ab. (2079) |
| 56 | or/3-55 (439409) |
| 57 | economics/ (203011) |
| 58 | "cost benefit analysis"/ (60778) |
| 59 | socioeconomics/ (100782) |
| 60 | health economics/ (31596) |
| 61 | pharmacoeconomics/ (4331) |
| 62 | or/57-61 (360904) |
| 63 | (econom$ or cost or costs or costly or costing or price or prices or pricing or pharmacoeconomic$).tw. (507165) |
| 64 | (expenditure$ not energy).tw. (20336) |
| 65 | (value adj1 money).tw. (22) |
| 66 | budget$.tw. (20992) |
| 67 | or/63-66 (528132) |
| 68 | 62 or 67 (772561) |
| 69 | (metabolic adj cost).ti,ab,sh. (747) |
| 70 | ((energy or oxygen) adj cost).ti,ab,sh. (3288) |
| 71 | 68 not (69 or 70) (768749) |
| 72 | hospitalization/ or "length of stay"/ or patient admission/ or patient readmission/ (298948) |
| 73 | Health Resources/ (71535) |
| 74 | (length adj2 stay).ti,ab. (31313) |
| 75 | (duration adj2 stay).ti,ab. (2318) |
| 76 | (hospital adj (visit$ or contact$ or attendance$ or admission$ or episode$)).ti,ab. (28409) |
| 77 | hospital costs.ti,ab. (4555) |
| 78 | (time adj2 discharge).ti,ab. (4432) |
| 79 | hospital day$.ti,ab. (5004) |
| 80 | ((patient$ or inpatient$ or in-patient$) adj (cost$ or stay)).ti,ab. (4612) |
| 81 | (number adj2 (nights or days)).ti,ab. (9578) |
| 82 | (primary care adj (visit$ or contact$ or attendance$ or admission$ or episode$)).ti,ab. (839) |
| 83 | (surgery adj (visit$ or contact$ or attendance$ or admission$ or episode$)).ti,ab. (248) |
| 84 | ((clinic or surgery or hospital or "accident and emergency") adj2 (work-flow or work flow)).ti,ab. (5) |
| 85 | (consultation$ adj2 (time or length)).ti,ab. (1090) |
| 86 | (hospitalization$ or hospitalisation$ or rehospitalization$ or rehospitalisation$ or re-hospitalization$ or re-hospitalisation$).ti,ab. (109959) |
| 87 | or/72-86 (449902) |
| 88 | 71 or 87 (1147344) |
| 89 | 56 and 88 (76004) |
| 90 | double-blind$.mp. (168936) |
| 91 | placebo$.tw. (179392) |
| 92 | blind$.tw. (237886) |
| 93 | or/90-92 (348272) |
| 94 | 89 and 93 (1127) |

**e) Ovid MEDLINE(R) <1946 to May Week 2 2012> Via OvidSP**

| **ID** | **Search** |
| --- | --- |
| 1 | ((self administer$ adj2 questionnaire$) or (self administer$ adj2 survey$) or (selfadminister$ adj2 interview$)).ti,ab. (11407) |
| 2 | self administer$.ti,ab. (18685) |
| 3 | 2 not 1 (7279) |
| 4 | self administration/ (8219) |
| 5 | self care/ (20482) |
| 6 | (self care or selfcare).ti,ab. (8196) |
| 7 | (self manag$ or selfmanag$).ti,ab. (6120) |
| 8 | (self monitor$ or selfmonitor$).ti,ab. (3580) |
| 9 | (self help or selfhelp).ti,ab. (3903) |
| 10 | (self diagnos$ or selfdiagnos$ or self assess$ or selfassess$).ti,ab. (7806) |
| 11 | blood glucose self-monitoring/ (3603) |
| 12 | Self initiated intervention$.ti,ab. (0) |
| 13 | Self efficacy.ti,ab. (9233) |
| 14 | Self Efficacy/ (9738) |
| 15 | self medication/ (3692) |
| 16 | ((pharmacist$ or pharmacy or pharmacies) adj2 support$).ti,ab. (260) |
| 17 | ((pharmacist$ or pharmacy or pharmacies) adj2 assist$).ti,ab. (298) |
| 18 | ((pharmacist or pharmacy or pharmacies) adj2 (advice or advis$ or inform$)).ti,ab. (404) |
| 19 | pharmaceutical care.ti,ab. (1085) |
| 20 | (self medicat$ or selfmedicat$ or self remed$ or selfremed$).ti,ab. (2260) |
| 21 | (self treat$ or selftreat$ or self cure or selfcure).ti,ab. (1234) |
| 22 | self help groups/ (7313) |
| 23 | Social Support/ (44651) |
| 24 | Social support$.ti,ab. (17533) |
| 25 | (group adj1 (support$ or advice or advis$ or monitor$ or intervention$ or train$ or instruction or consult$ or assist$ or education or educate or information)).ti,ab. (19902) |
| 26 | (peer adj1 (support$ or advice or advis$ or monitor$ or intervention$ or train$ or instruction or consult$ or assist$ or education or educate or information)).ti,ab. (2096) |
| 27 | expert patient$.ti,ab. (124) |
| 28 | Psychosocial support.ti,ab. (1391) |
| 29 | Befriend$.ti,ab. (127) |
| 30 | Health trainer$.ti,ab. (16) |
| 31 | Telemedicine/ (9037) |
| 32 | telemedicine.ti,ab. (4870) |
| 33 | telecare.ti,ab. (266) |
| 34 | telenursing.ti,ab. (68) |
| 35 | telemonitor$.ti,ab. (411) |
| 36 | telehealth.ti,ab. (1095) |
| 37 | Remote Consultation/ (3255) |
| 38 | ((telephon$ or remote or phone) adj2 (follow$ or support or consult$ or advice or advis$ or intervention$ or train$ or instruction or assis$ or educate or education or information or monitor$)).ti,ab. (6832) |
| 39 | Case Management/ (7610) |
| 40 | case management.ti,ab. (6359) |
| 41 | Action plan$.ti,ab. (2774) |
| 42 | Management plan$.ti,ab. (3054) |
| 43 | Management program$.ti,ab. (6606) |
| 44 | care plan$.ti,ab. (7054) |
| 45 | (nurse adj2 educator$).ti,ab. (2129) |
| 46 | patient education.ti,ab. (9527) |
| 47 | Patient Education as Topic/ (64554) |
| 48 | (patient adj2 (education or advice or advis$ or instruct$ or educate or train$)).ti,ab. (12724) |
| 49 | Consumer health information.ti,ab. (189) |
| 50 | patient informat$.ti,ab. (4002) |
| 51 | ((financial or monetary or money) adj2 (incentive$ or competition$ or contest$ or lotter$ or reward$ or prize$)).ti,ab. (3140) |
| 52 | (contingent payment$ or deposit contract$).ti,ab. (22) |
| 53 | Decision Making/ (59912) |
| 54 | (decision$ adj2 support$).ti,ab. (7383) |
| 55 | (decision$ adj2 aid$).ti,ab. (2138) |
| 56 | (shared adj2 decision$).ti,ab. (1548) |
| 57 | or/3-56 (314750) |
| 58 | economics/ (26272) |
| 59 | exp "Costs and Cost Analysis"/ (164383) |
| 60 | Value of Life/ (5212) |
| 61 | economics, dental/ (1840) |
| 62 | exp economics, hospital/ (17897) |
| 63 | economics, medical/ (8463) |
| 64 | economics, nursing/ (3861) |
| 65 | economics, pharmaceutical/ (2327) |
| 66 | or/58-64 (212386) |
| 67 | (econom$ or cost or costs or costly or costing or price or prices or pricing or pharmacoeconomic$).tw. (370078) |
| 68 | (expenditure$ not energy).tw. (15044) |
| 69 | (value adj1 money).tw. (18) |
| 70 | budget$.tw. (15278) |
| 71 | or/67-70 (385856) |
| 72 | 66 or 71 (488196) |
| 73 | (metabolic adj cost).ti,ab,sh. (637) |
| 74 | ((energy or oxygen) adj cost).ti,ab,sh. (2417) |
| 75 | 72 not (73 or 74) (485227) |
| 76 | hospitalization/ or "length of stay"/ or patient admission/ or patient readmission/ (124493) |
| 77 | Health Resources/ (7697) |
| 78 | (length adj2 stay).ti,ab. (21033) |
| 79 | (duration adj2 stay).ti,ab. (1603) |
| 80 | (hospital adj (visit$ or contact$ or attendance$ or admission$ or episode$)).ti,ab. (20388) |
| 81 | hospital costs.ti,ab. (3345) |
| 82 | (time adj2 discharge).ti,ab. (3045) |
| 83 | hospital day$.ti,ab. (3626) |
| 84 | ((patient$ or inpatient$ or in-patient$) adj (cost$ or stay)).ti,ab. (3090) |
| 85 | (number adj2 (nights or days)).ti,ab. (7134) |
| 86 | (primary care adj (visit$ or contact$ or attendance$ or admission$ or episode$)).ti,ab. (670) |
| 87 | (surgery adj (visit$ or contact$ or attendance$ or admission$ or episode$)).ti,ab. (177) |
| 88 | ((clinic or surgery or hospital or "accident and emergency") adj2 (work-flow or work flow)).ti,ab. (3) |
| 89 | (consultation$ adj2 (time or length)).ti,ab. (791) |
| 90 | (hospitalization$ or hospitalisation$ or rehospitalization$ or rehospitalisation$ or re-hospitalization$ or re-hospitalisation$).ti,ab. (76212) |
| 91 | or/76-90 (214119) |
| 92 | 75 or 91 (662098) |
| 93 | randomized controlled trial.pt. (326816) |
| 94 | controlled clinical trial.pt. (84077) |
| 95 | randomized.ab. (230964) |
| 96 | placebo.ab. (131080) |
| 97 | clinical trials as topic.sh. (159974) |
| 98 | randomly.ab. (166761) |
| 99 | trial.ti. (99783) |
| 100 | or/93-99 (757942) |
| 101 | (animals not (humans and animals)).sh. (3623284) |
| 102 | 100 not 101 (698837) |
| 103 | 57 and 92 and 102 (5804) |

**f) Ovid MEDLINE(R) In-Process & Other Non-Indexed Citations <May 16, 2012> Via OvidSP**

| **ID** | **Search** |
| --- | --- |
| 1 | ((self administer$ adj2 questionnaire$) or (self administer$ adj2 survey$) or (selfadminister$ adj2 interview$)).ti,ab. (615) |
| 2 | self administer$.ti,ab. (948) |
| 3 | 2 not 1 (333) |
| 4 | self administration/ (0) |
| 5 | self care/ (0) |
| 6 | (self care or selfcare).ti,ab. (382) |
| 7 | (self manag$ or selfmanag$).ti,ab. (511) |
| 8 | (self monitor$ or selfmonitor$).ti,ab. (230) |
| 9 | (self help or selfhelp).ti,ab. (168) |
| 10 | (self diagnos$ or selfdiagnos$ or self assess$ or selfassess$).ti,ab. (431) |
| 11 | blood glucose self-monitoring/ (0) |
| 12 | Self initiated intervention$.ti,ab. (0) |
| 13 | Self efficacy.ti,ab. (629) |
| 14 | Self Efficacy/ (0) |
| 15 | self medication/ (0) |
| 16 | ((pharmacist$ or pharmacy or pharmacies) adj2 support$).ti,ab. (18) |
| 17 | ((pharmacist$ or pharmacy or pharmacies) adj2 assist$).ti,ab. (17) |
| 18 | ((pharmacist or pharmacy or pharmacies) adj2 (advice or advis$ or inform$)).ti,ab. (16) |
| 19 | pharmaceutical care.ti,ab. (64) |
| 20 | (self medicat$ or selfmedicat$ or self remed$ or selfremed$).ti,ab. (127) |
| 21 | (self treat$ or selftreat$ or self cure or selfcure).ti,ab. (54) |
| 22 | self help groups/ (0) |
| 23 | Social Support/ (0) |
| 24 | Social support$.ti,ab. (960) |
| 25 | (group adj1 (support$ or advice or advis$ or monitor$ or intervention$ or train$ or instruction or consult$ or assist$ or education or educate or information)).ti,ab. (1223) |
| 26 | (peer adj1 (support$ or advice or advis$ or monitor$ or intervention$ or train$ or instruction or consult$ or assist$ or education or educate or information)).ti,ab. (163) |
| 27 | expert patient$.ti,ab. (10) |
| 28 | Psychosocial support.ti,ab. (68) |
| 29 | Befriend$.ti,ab. (14) |
| 30 | Health trainer$.ti,ab. (1) |
| 31 | Telemedicine/ (0) |
| 32 | telemedicine.ti,ab. (299) |
| 33 | telecare.ti,ab. (28) |
| 34 | telenursing.ti,ab. (2) |
| 35 | telemonitor$.ti,ab. (57) |
| 36 | telehealth.ti,ab. (73) |
| 37 | Remote Consultation/ (0) |
| 38 | ((telephon$ or remote or phone) adj2 (follow$ or support or consult$ or advice or advis$ or intervention$ or train$ or instruction or assis$ or educate or education or information or monitor$)).ti,ab. (468) |
| 39 | Case Management/ (0) |
| 40 | case management.ti,ab. (205) |
| 41 | Action plan$.ti,ab. (204) |
| 42 | Management plan$.ti,ab. (213) |
| 43 | Management program$.ti,ab. (443) |
| 44 | care plan$.ti,ab. (275) |
| 45 | (nurse adj2 educator$).ti,ab. (73) |
| 46 | patient education.ti,ab. (410) |
| 47 | Patient Education as Topic/ (0) |
| 48 | (patient adj2 (education or advice or advis$ or instruct$ or educate or train$)).ti,ab. (585) |
| 49 | Consumer health information.ti,ab. (5) |
| 50 | patient informat$.ti,ab. (185) |
| 51 | ((financial or monetary or money) adj2 (incentive$ or competition$ or contest$ or lotter$ or reward$ or prize$)).ti,ab. (193) |
| 52 | (contingent payment$ or deposit contract$).ti,ab. (0) |
| 53 | Decision Making/ (0) |
| 54 | (decision$ adj2 support$).ti,ab. (594) |
| 55 | (decision$ adj2 aid$).ti,ab. (151) |
| 56 | (shared adj2 decision$).ti,ab. (134) |
| 57 | or/3-56 (8452) |
| 58 | economics/ (0) |
| 59 | exp "Costs and Cost Analysis"/ (0) |
| 60 | Value of Life/ (0) |
| 61 | economics, dental/ (0) |
| 62 | exp economics, hospital/ (0) |
| 63 | economics, medical/ (0) |
| 64 | economics, nursing/ (0) |
| 65 | economics, pharmaceutical/ (0) |
| 66 | or/58-64 (0) |
| 67 | (econom$ or cost or costs or costly or costing or price or prices or pricing or pharmacoeconomic$).tw. (28822) |
| 68 | (expenditure$ not energy).tw. (724) |
| 69 | (value adj1 money).tw. (2) |
| 70 | budget$.tw. (1467) |
| 71 | or/67-70 (30285) |
| 72 | 66 or 71 (30285) |
| 73 | (metabolic adj cost).ti,ab,sh. (42) |
| 74 | ((energy or oxygen) adj cost).ti,ab,sh. (157) |
| 75 | 72 not (73 or 74) (30087) |
| 76 | hospitalization/ or "length of stay"/ or patient admission/ or patient readmission/ (0) |
| 77 | Health Resources/ (0) |
| 78 | (length adj2 stay).ti,ab. (1158) |
| 79 | (duration adj2 stay).ti,ab. (90) |
| 80 | (hospital adj (visit$ or contact$ or attendance$ or admission$ or episode$)).ti,ab. (985) |
| 81 | hospital costs.ti,ab. (122) |
| 82 | (time adj2 discharge).ti,ab. (183) |
| 83 | hospital day$.ti,ab. (106) |
| 84 | ((patient$ or inpatient$ or in-patient$) adj (cost$ or stay)).ti,ab. (142) |
| 85 | (number adj2 (nights or days)).ti,ab. (344) |
| 86 | (primary care adj (visit$ or contact$ or attendance$ or admission$ or episode$)).ti,ab. (31) |
| 87 | (surgery adj (visit$ or contact$ or attendance$ or admission$ or episode$)).ti,ab. (8) |
| 88 | ((clinic or surgery or hospital or "accident and emergency") adj2 (work-flow or work flow)).ti,ab. (0) |
| 89 | (consultation$ adj2 (time or length)).ti,ab. (37) |
| 90 | (hospitalization$ or hospitalisation$ or rehospitalization$ or rehospitalisation$ or re-hospitalization$ or re-hospitalisation$).ti,ab. (3472) |
| 91 | or/76-90 (6050) |
| 92 | 75 or 91 (35061) |
| 93 | randomized controlled trial.pt. (608) |
| 94 | controlled clinical trial.pt. (25) |
| 95 | randomized.ab. (11733) |
| 96 | placebo.ab. (4872) |
| 97 | clinical trials as topic.sh. (0) |
| 98 | randomly.ab. (11517) |
| 99 | trial.ti. (4683) |
| 100 | or/93-99 (27008) |
| 101 | (animals not (humans and animals)).sh. (1) |
| 102 | 100 not 101 (27008) |
| 103 | 57 and 92 and 102 (209) |

**g) NHS EED – via Cochrane Library (searched 20 06-2012)**

| **ID** | **Search** |
| --- | --- |
| **ID** | **Search** |
| #1 | [(self NEXT administer*) in Economic Evaluations](http://onlinelibrary.wiley.com/o/cochrane/searchHistory?mode=runquery&qnum=1) |
| #2 | [MeSH descriptor Self Administration, this term only](http://onlinelibrary.wiley.com/o/cochrane/searchHistory?mode=runquery&qnum=2) |
| #3 | [MeSH descriptor Self Care, this term only](http://onlinelibrary.wiley.com/o/cochrane/searchHistory?mode=runquery&qnum=3) |
| #4 | ["self care" or (selfcare) or (self NEXT manage*) or (selfmonitor*) or (self NEXT monitor*) in Economic Evaluations](http://onlinelibrary.wiley.com/o/cochrane/searchHistory?mode=runquery&qnum=4) |
| #5 | [(selfhelp) or "self help" or (self NEXT diagnos*) or (selfdiagnos*) in Economic Evaluations](http://onlinelibrary.wiley.com/o/cochrane/searchHistory?mode=runquery&qnum=5) |
| #6 | [(self NEXT assess*) or (selfassess*) in Economic Evaluations](http://onlinelibrary.wiley.com/o/cochrane/searchHistory?mode=runquery&qnum=6) |
| #7 | [MeSH descriptor Blood Glucose Self-Monitoring, this term only](http://onlinelibrary.wiley.com/o/cochrane/searchHistory?mode=runquery&qnum=7) |
| #8 | ["self inititiated intervention" in Economic Evaluations](http://onlinelibrary.wiley.com/o/cochrane/searchHistory?mode=runquery&qnum=8) |
| #9 | [(self NEXT initiated NEXT intervent*) in Economic Evaluations](http://onlinelibrary.wiley.com/o/cochrane/searchHistory?mode=runquery&qnum=9) |
| #10 | [MeSH descriptor Self Efficacy, this term only](http://onlinelibrary.wiley.com/o/cochrane/searchHistory?mode=runquery&qnum=10) |
| #11 | [MeSH descriptor Self Medication explode all trees](http://onlinelibrary.wiley.com/o/cochrane/searchHistory?mode=runquery&qnum=11) |
| #12 | ["self efficacy" or (pharmacist* or pharmacy or pharmacies) NEAR/2 support* in Economic Evaluations](http://onlinelibrary.wiley.com/o/cochrane/searchHistory?mode=runquery&qnum=12) |
| #13 | [(pharmacist* or pharmacy or pharmacies) NEAR/2 assist* or (pharmacist* or pharmacy or pharmacies) NEAR/2 (advice or advis* or inform*) or "pharmaceutical care" in Economic Evaluations](http://onlinelibrary.wiley.com/o/cochrane/searchHistory?mode=runquery&qnum=13) |
| #14 | [(self NEXT medicat*) or (selfmedicat*) or (self NEXT remed*) or (selfremed*) in Economic Evaluations](http://onlinelibrary.wiley.com/o/cochrane/searchHistory?mode=runquery&qnum=14) |
| #15 | [(self NEXT treat*) or (selftreat*) or "self cure" or (selfcure) in Economic Evaluations](http://onlinelibrary.wiley.com/o/cochrane/searchHistory?mode=runquery&qnum=15) |
| #16 | [MeSH descriptor Self-Help Groups, this term only](http://onlinelibrary.wiley.com/o/cochrane/searchHistory?mode=runquery&qnum=16) |
| #17 | [MeSH descriptor Social Support explode all trees](http://onlinelibrary.wiley.com/o/cochrane/searchHistory?mode=runquery&qnum=17) |
| #18 | [(social NEXT support*) in Economic Evaluations](http://onlinelibrary.wiley.com/o/cochrane/searchHistory?mode=runquery&qnum=18) |
| #19 | [(group NEAR/1 (support* or advice or advis* or monitor* or intervention* or train* or instruction or consult* or assist* or education or information)) in Economic Evaluations](http://onlinelibrary.wiley.com/o/cochrane/searchHistory?mode=runquery&qnum=19) |
| #20 | [(peer NEAR/1 (support* or advice or advis* or monitor* or intervention* or train* or instruction or consult* or assist* or education or information)) in Economic Evaluations](http://onlinelibrary.wiley.com/o/cochrane/searchHistory?mode=runquery&qnum=20) |
| #21 | [(expert NEXT patient*) or "psychosocial support" or (befriend*) or (health NEXT trainer*) in Economic Evaluations](http://onlinelibrary.wiley.com/o/cochrane/searchHistory?mode=runquery&qnum=21) |
| #22 | [MeSH descriptor Telemedicine, this term only](http://onlinelibrary.wiley.com/o/cochrane/searchHistory?mode=runquery&qnum=22) |
| #23 | [(telemedicine) or (telecare) or (telenursing) or (telemonitor*) or (telehealth) in Economic Evaluations](http://onlinelibrary.wiley.com/o/cochrane/searchHistory?mode=runquery&qnum=23) |
| #24 | [MeSH descriptor Remote Consultation, this term only](http://onlinelibrary.wiley.com/o/cochrane/searchHistory?mode=runquery&qnum=24) |
| #25 | [(telephon* or remote or phone) NEAR/2 (follow* or support or consult* or advice or advis* or intervention or train* or instruction or assist* or educate or education or information or monitor*) in Economic Evaluations](http://onlinelibrary.wiley.com/o/cochrane/searchHistory?mode=runquery&qnum=25) |
| #26 | ["case management" or (action NEXT plan*) or (management NEXT plan*) or (management NEXT program*) or (care NEXT plan*) in Economic Evaluations](http://onlinelibrary.wiley.com/o/cochrane/searchHistory?mode=runquery&qnum=26) |
| #27 | [(nurse NEAR/2 educator*) in Economic Evaluations](http://onlinelibrary.wiley.com/o/cochrane/searchHistory?mode=runquery&qnum=27) |
| #28 | ["patient education" in Economic Evaluations](http://onlinelibrary.wiley.com/o/cochrane/searchHistory?mode=runquery&qnum=28) |
| #29 | [MeSH descriptor Patient Education as Topic, this term only](http://onlinelibrary.wiley.com/o/cochrane/searchHistory?mode=runquery&qnum=29) |
| #30 | [MeSH descriptor Case Management, this term only](http://onlinelibrary.wiley.com/o/cochrane/searchHistory?mode=runquery&qnum=30) |
| #31 | [(patient NEAR/2 (education or advice or advis* or instruct* or educate or train*)) in Economic Evaluations](http://onlinelibrary.wiley.com/o/cochrane/searchHistory?mode=runquery&qnum=31) |
| #32 | ["consumer health information" or "patient information" in Economic Evaluations](http://onlinelibrary.wiley.com/o/cochrane/searchHistory?mode=runquery&qnum=32) |
| #33 | [(financial or monetary or money) NEAR/2 (incentive* or competition* or contest* or lotter* or reward* or prize*) in Economic Evaluations](http://onlinelibrary.wiley.com/o/cochrane/searchHistory?mode=runquery&qnum=33) |
| #34 | [(contingent NEXT payment*) or (deposit NEXT contract*) or (decision NEAR/2 support*) or (decision NEAR/2 aid*) or (shared NEAR/2 decision*) in Economic Evaluations](http://onlinelibrary.wiley.com/o/cochrane/searchHistory?mode=runquery&qnum=34) |
| #35 | [MeSH descriptor Decision Making, this term only](http://onlinelibrary.wiley.com/o/cochrane/searchHistory?mode=runquery&qnum=35) |
| #36 | [(#1 OR #2 OR #3 OR #4 OR #5 OR #6 OR #7 OR #8 OR #9 OR #10 OR #11 OR #12 OR #13 OR #14 OR #15 OR #16 OR #17 OR #18 OR #19 OR #20 OR #21 OR #22 OR #23 OR #24 OR #25 OR #26 OR #27 OR #28 OR #29 OR #30 OR #31 OR #32 OR #33 OR #34 OR #35)](http://onlinelibrary.wiley.com/o/cochrane/searchHistory?mode=runquery&qnum=36) |
| #37 | [MeSH descriptor Hospitalization explode all trees](http://onlinelibrary.wiley.com/o/cochrane/searchHistory?mode=runquery&qnum=37) |
| #38 | [MeSH descriptor Health Resources, this term only](http://onlinelibrary.wiley.com/o/cochrane/searchHistory?mode=runquery&qnum=38) |
| #39 | [(length NEAR/2 stay) or (duration NEAR/2 stay) or (hospital NEAR/1 (visit* or contact* or attendance* or admission* or episode*)) or (time NEAR/2 discharge) or (hospital NEXT day*) in Economic Evaluations](http://onlinelibrary.wiley.com/o/cochrane/searchHistory?mode=runquery&qnum=39) |
| #40 | [(patient* or inpatient* or in-patient*) NEAR/1 (cost* or stay) or (number NEAR/2 (nights or days)) in Economic Evaluations](http://onlinelibrary.wiley.com/o/cochrane/searchHistory?mode=runquery&qnum=40) |
| #41 | ["primary care" NEAR/2 (visit* or contact* or attendance* or admission* or episode*) or (surgery NEAR/2 (visit* or contact* or attendance* or admission* or episode*)) in Economic Evaluations](http://onlinelibrary.wiley.com/o/cochrane/searchHistory?mode=runquery&qnum=41) |
| #42 | [(clinic or surgery or hospital or "accident and emergency") NEAR/2 (work-flow or "work flow") in Economic Evaluations](http://onlinelibrary.wiley.com/o/cochrane/searchHistory?mode=runquery&qnum=42) |
| #43 | [(consultation* NEAR/2 (time or length)) or (hospitalization* or hospitalisation* or rehospitalization* or rehospitalisation* or re-hospitalization* or re-hospitalisation*) or "hospital costs" in Economic Evaluations](http://onlinelibrary.wiley.com/o/cochrane/searchHistory?mode=runquery&qnum=43) |
| #44 | [(#37 OR #38 OR #39 OR #40 OR #41 OR #42 OR #43)](http://onlinelibrary.wiley.com/o/cochrane/searchHistory?mode=runquery&qnum=44) |
| #45 | [(#36 AND #44)](http://onlinelibrary.wiley.com/o/cochrane/searchHistory?mode=runquery&qnum=45) |

**h) PsycINFO <1806 to May Week 3 2012> Via OvidSP**

| **ID** | **Search** |
| --- | --- |
| 1 | ((self administer$ adj2 questionnaire$) or (self administer$ adj2 survey$) or (selfadminister$ adj2 interview$)).ti,ab. (4088) |
| 2 | self administer$.ti,ab. (8158) |
| 3 | 2 not 1 (4071) |
| 4 | Drug Self Administration/ (1142) |
| 5 | exp Self Help Techniques/ (7116) |
| 6 | Self Monitoring/ (2211) |
| 7 | (self care or selfcare).ti,ab. (4718) |
| 8 | (self manag$ or selfmanag$).ti,ab. (4597) |
| 9 | (self monitor$ or selfmonitor$).ti,ab. (4179) |
| 10 | (self help or selfhelp).ti,ab. (5924) |
| 11 | (self diagnos$ or selfdiagnos$ or self assess$ or selfassess$).ti,ab. (5035) |
| 12 | Self initiated intervention$.ti,ab. (0) |
| 13 | Self efficacy.ti,ab. (19044) |
| 14 | Self Efficacy/ (12331) |
| 15 | self medication/ (457) |
| 16 | ((pharmacist$ or pharmacy or pharmacies) adj2 support$).ti,ab. (25) |
| 17 | ((pharmacist$ or pharmacy or pharmacies) adj2 assist$).ti,ab. (28) |
| 18 | ((pharmacist or pharmacy or pharmacies) adj2 (advice or advis$ or inform$)).ti,ab. (37) |
| 19 | pharmaceutical care.ti,ab. (79) |
| 20 | (self medicat$ or selfmedicat$ or self remed$ or selfremed$).ti,ab. (1004) |
| 21 | (self treat$ or selftreat$ or self cure or selfcure).ti,ab. (311) |
| 22 | exp Support Groups/ (4553) |
| 23 | Social Support/ (23928) |
| 24 | Social support$.ti,ab. (28222) |
| 25 | (group adj1 (support$ or advice or advis$ or monitor$ or intervention$ or train$ or instruction or consult$ or assist$ or education or educate or information)).ti,ab. (13907) |
| 26 | (peer adj1 (support$ or advice or advis$ or monitor$ or intervention$ or train$ or instruction or consult$ or assist$ or education or educate or information)).ti,ab. (2962) |
| 27 | expert patient$.ti,ab. (62) |
| 28 | Psychosocial support.ti,ab. (867) |
| 29 | Befriend$.ti,ab. (309) |
| 30 | Health trainer$.ti,ab. (8) |
| 31 | Telemedicine/ (1805) |
| 32 | telemedicine.ti,ab. (696) |
| 33 | telecare.ti,ab. (96) |
| 34 | telenursing.ti,ab. (13) |
| 35 | telemonitor$.ti,ab. (62) |
| 36 | telehealth.ti,ab. (429) |
| 37 | ((telephon$ or remote or phone) adj2 (follow$ or support or consult$ or advice or advis$ or intervention$ or train$ or instruction or assis$ or educate or education or information or monitor$)).ti,ab. (2353) |
| 38 | exp Case Management/ (2565) |
| 39 | case management.ti,ab. (3412) |
| 40 | Action plan$.ti,ab. (1591) |
| 41 | Management plan$.ti,ab. (614) |
| 42 | Management program$.ti,ab. (2754) |
| 43 | care plan$.ti,ab. (1847) |
| 44 | (nurse adj2 educator$).ti,ab. (469) |
| 45 | patient education.ti,ab. (1727) |
| 46 | (patient adj2 (education or advice or advis$ or instruct$ or educate or train$)).ti,ab. (2517) |
| 47 | Consumer health information.ti,ab. (23) |
| 48 | patient informat$.ti,ab. (544) |
| 49 | ((financial or monetary or money) adj2 (incentive$ or competition$ or contest$ or lotter$ or reward$ or prize$)).ti,ab. (2415) |
| 50 | (contingent payment$ or deposit contract$).ti,ab. (26) |
| 51 | Decision Making/ (38754) |
| 52 | (decision$ adj2 support$).ti,ab. (2711) |
| 53 | (decision$ adj2 aid$).ti,ab. (1006) |
| 54 | (shared adj2 decision$).ti,ab. (944) |
| 55 | 5or/3-54 (161780) |
| 56 | Economics/ (12133) |
| 57 | Health Care Economics/ (291) |
| 58 | exp Costs/ and Cost Analysis/ (0) |
| 59 | Pharmacoeconomics/ (182) |
| 60 | or/56-59 (12545) |
| 61 | (econom$ or cost or costs or costly or costing or price or prices or pricing or pharmacoeconomic$).tw. (129318) |
| 62 | (expenditure$ not energy).tw. (4346) |
| 63 | (value adj1 money).tw. (26) |
| 64 | budget$.tw. (4840) |
| 65 | or/61-64 (134996) |
| 66 | 60 or 65 (135864) |
| 67 | metabolic adj cost).ti,ab,sh. (47) |
| 68 | ((energy or oxygen) adj cost).ti,ab,sh. (153) |
| 69 | 66 not (67 or 68) (135669) |
| 70 | hospitalization/ (4209) |
| 71 | exp Hospital Admission/ (3535) |
| 72 | Treatment Duration/ (2959) |
| 73 | Health Care Utilization/ (10577) |
| 74 | (length adj2 stay).ti,ab. (2943) |
| 75 | (duration adj2 stay).ti,ab. (198) |
| 76 | (hospital adj (visit$ or contact$ or attendance$ or admission$ or episode$)).ti,ab. (3068) |
| 77 | hospital costs.ti,ab. (153) |
| 78 | (time adj2 discharge).ti,ab. (489) |
| 79 | hospital day$.ti,ab. (327) |
| 80 | ((patient$ or inpatient$ or in-patient$) adj (cost$ or stay)).ti,ab. (542) |
| 81 | (number adj2 (nights or days)).ti,ab. (1513) |
| 82 | (primary care adj (visit$ or contact$ or attendance$ or admission$ or episode$)).ti,ab. (272) |
| 83 | (surgery adj (visit$ or contact$ or attendance$ or admission$ or episode$)).ti,ab. (8) |
| 84 | ((clinic or surgery or hospital or "accident and emergency") adj2 (work-flow or work flow)).ti,ab. (0) |
| 85 | (consultation$ adj2 (time or length)).ti,ab. (215) |
| 86 | (hospitalization$ or hospitalisation$ or rehospitalization$ or rehospitalisation$ or re-hospitalization$ or re-hospitalisation$).ti,ab. (17964) |
| 87 | or/70-86 (40346) |
| 88 | 69 or 87 (171172) |
| 89 | 55 and 88 (15697) |
| 90 | clinical trials/ or "treatment outcome clinical trial".md. or ((randomi?ed adj7 trial*) or ((single or doubl* or tripl* or treb*) and (blind* or mask*)) or (controlled adj3 trial*) or (clinical adj2 trial*)).ti,ab,id. (60572) |
| 91 | 89 and 90 (975) |
