# Supplementary material for: Systematic meta-review of supported self-management for asthma: a healthcare perspective
Source: BMC Med. 2017 Mar 17;15:64. doi: 10.1186/s12916-017-0823-7 (PMC5356253; doi:10.1186/s12916-017-0823-7)
Supplement: Additional file 2: — Dates of initial and update searches. (DOCX 21 kb) [file 12916_2017_823_MOESM2_ESM.docx]

**Additional file 2: Search dates (PRISMS)**

**PRISMS initial search date for systematic reviews: November 2012**

**PRISMS updated systematic review search dates: December 2012 to March 2015**

**Table 1 Search dates for the update RCTs.** Included systematic reviews were grouped according to the populations studied (children, adults, or ethnic minority groups) and the search dates of the reviews extracted. Dates for update RCT search were set from the date of the latest review search.

| **Review** | **Date of search** | **Date range of included RCTs** | **Last search date in the SRs and dates for updated RCT searches** |
| --- | --- | --- | --- |
| **SRs including children** | | | |
| Bernard Bonnin, 1995 | 1991 | 1977-1991 | **Last SR search date: September 2010**  **Dates for RCT search:**  **September 2010 to March 2015** |
| Bhogal 2006 | November 2004 | 1990-2004 |  |
| Zemek, 2008 | March 2006 | 1986-2005 |  |
| Boyd, 2009 | May 2008 | 1985-2007 |  |
| Coffman, 2009 | Not stated | 1980-2008 |  |
| Bravata 2009 | May 2006 | 1985-2006 |  |
| Stinson 2009 | January 2008 | 2003-2007 |  |
| Kirk 2012 | Sept 2010 | 1999-2009 |  |
| Toelle, 2004 | June 2004 | 1990-2001 |  |
| Gibson 2004 | Not stated | 1987-2003 |  |
| Bussey Smith, 2007 | October 2005 | 1986-2005 |  |
| Ring, 2007 | 2006 | 1993-2006 |  |
| Tapp, 2010 | November 2009 | 1979-2009 |  |
| **SRs including adults** | | | |
| Gibson, 2002 | Not stated | 1986-2001 | **Last SR search date: October 2012**  **Dates for RCT search:**  **October 2012 to March 2015** |
| Powell, 2009 | Not stated | 1994-2001 |  |
| Newman, 2004 | 2002 | 1984-2002 |  |
| Moullec, 2012 | August 2010 | 1986-2010 |  |
| de Jongh 2012 | June 2009 | 2004-2009 |  |
| Denford 2013 | October 2012 | 1993-2011 |  |
| Toelle, 2004 | June 2004 | 1990-2001 |  |
| Gibson 2004 | Not stated | 1987-2003 |  |
| Bussey Smith, 2007 | October 2005 | 1986-2005 |  |
| Ring, 2007 | 2006 | 1993-2006 |  |
| Tapp, 2010 | November 2009 | 1979-2009 |  |
| **Cultural Groups** | | | |
| Postma, 2009 | Not stated | 1996-2008 | **Last SR search date: January 2011**  **Dates for RCT search:**  **January 2011 to March 2015** |
| Bailey, 2009 | May 2008 | 1986-2008 |  |
| Welsh, 2011 | January 2011 | 1986-2010 |  |
| Chang, 2010 | Updated January 2011 | 1995-2010 |  |
| Press 2012 | Autumn 2010 | 1979-2009 |  |
