# Supplementary material for: Systematic meta-review of supported self-management for asthma: a healthcare perspective
Source: BMC Med. 2017 Mar 17;15:64. doi: 10.1186/s12916-017-0823-7 (PMC5356253; doi:10.1186/s12916-017-0823-7)
Supplement: Additional file 3: — Detailed PICOS table and inclusion/exclusion criteria. (DOCX 22 kb) [file 12916_2017_823_MOESM3_ESM.docx]

**Additional file 3.**

**Table S3a. Detailed PICOS search strategy and sources for the reviews**

|  | Description, inclusion/exclusion criteria, process | |
| --- | --- | --- |
|  | PRISMS systematic meta-review | RECURSIVE systematic review |
| Population | People with asthma, including adults and/or children, ethnic minorities and groups who were perceived as finding services ‘hard to reach’. Trials of generic self-management support was included if sub-group data for people with asthma were reported. | People with asthma (within a wider search of long-term conditions) asthma. We excluded subjects < 18 years of age and studies conducted in the developing world. |
| Intervention | Any systematic review which focused on, or explicitly incorporated, strategies to support self-management | Self-management support interventions |
| Comparator | Typically ‘usual care’. The nature of the control service was noted and accommodated within our analysis, but papers were not excluded on this basis. | Typically “usual care”. We also included studies that compared self-management support interventions with more “intense usual care” interventions. |
| Outcomes | Use of healthcare services (including unscheduled use of healthcare services and hospital admission rates), health outcomes (including asthma control), quality of life, process/intermediate outcomes (ownership of asthma action plans, self-efficacy). | Healthcare utilisation with a focus on comprehensive measures of costs (i.e. summaries including multiple sources of cost) or major cost drivers (i.e. hospital use). Other, more minor, costs (such as medication and primary care visits) were identified but not analysed.  Outcomes relating to quality of life and health outcomes (including standardised measures of disease-specific outcomes, and generic quality of life). |
| Settings | Any healthcare setting: hospital (in-patient or out-patient), community or remote (e.g. web based) settings | Any healthcare setting |
| Study design | Systematic reviews which had explicitly searched for RCTs. To be classified as a systematic review the report must describe: a systematic search strategy, a screening procedure, a data extraction procedure, a method of data synthesis.  We excluded previous versions of reviews if a more recent version had been published  The update search included RCTs which were included if they were published after the date of the last search in the included systematic reviews. Appendix 1 provides details of these dates | Randomised controlled trials |
| Databases | MEDLINE, EMBASE, CINAHL, PsycINFO, AMED, BNI, Cochrane Database of Systematic Reviews, Database of Abstracts of Reviews of Effects and ISI Proceedings (Web of Science). | CENTRAL, CINAHL, EconLit, EMBASE, Health Economics Evaluations Database, MEDLINE, MEDLINE In-Process & Other Non-Indexed Citations, NHS Economic Evaluation Database and the PsycINFO |
| Manual searching | Systematic Reviews, Health Education and Behaviour, Health Education Research, Journal of Behavioural Medicine, and Patient Education and Counseling. | Systematic reviews |
| Forward citations | A forward citation search was performed on all included systematic reviews using ISI Proceedings (Web of Science). The bibliographies of all eligible studies were scrutinised to identify additional possible studies. | None |
| Unpublished and in progress studies | Abstracts identified in the updated search were used to identify recently published trials | Abstracts identified in the updated search were used to identify recently published trials |
| Dates | Initial database search: January 1993 to July 2012. Manual and forward citations were completed in November 2012.  Update search was completed in March 2015. | Initial database search: inception to May 2012. Update search was conducted in September 2015 |
| Other exclusion criteria | We excluded previous versions of reviews if a more recent version had been published. We excluded papers not published in English. (Detailed exclusion criteria are in the following table) | Not applicable |

Table S3b PRISMS exclusion process quantitative meta-review

|  | **Exclusion criterion** |
| --- | --- |
| 1 | Exclude if it is not written in English |
| 2 | Exclude if does not include human participants |
| 3 | Exclude reviews published before 1993 |
| 4 | Exclude if it is not a systematic review of the literature |
| 5 | Exclude if the paper does not focus on, or include one or more of the exemplar LTCs. |
| 6 | Exclude if the focus is not about self-management support interventions |
| 7 | Exclude if the systematic review does not include RCTs in the search strategy |
| 8 | Exclude if does not measure one of the following outcomes: Use of healthcare services (including scheduled and unscheduled use of healthcare services and hospital admission rates), health outcomes (including biological markers of disease), symptoms, health behaviour, quality of life or self-efficacy |
| 9 | Exclude if the paper is a published conference abstract, thesis, protocol, or summary of other reviews |
| 10 | Exclude if the paper is a shorter and less detailed version of a Cochrane review or if there has been an updated version of it published |
| 11 | Exclude if unable to data extract the information on RCTs in the selected LTC separately from the rest of the findings |

**Table S3c RECURSIVE inclusion/exclusion criteria for full-text screening**

|  | **Inclusion/Exclusion criterion** (Yes No Unclear) |
| --- | --- |
| 1 | Is the study an RCT? |
| 2 | Does the intervention include a self-management component? |
| 3 | Does the study population have a long-term condition? |
| 4 | Does the study include data on quality of life |
| 5 | Does the study include data on health care utilisation and costs? |
| 6 | Are the participants adults? |
