# Supplementary material for: Systematic meta-review of supported self-management for asthma: a healthcare perspective
Source: BMC Med. 2017 Mar 17;15:64. doi: 10.1186/s12916-017-0823-7 (PMC5356253; doi:10.1186/s12916-017-0823-7)
Supplement: Additional file 4: — Quality assessment and weighting. (DOCX 43 kb) [file 12916_2017_823_MOESM4_ESM.docx]

**Additional file 4. Table S4a PRISMS: Asthma quality assessment results for systematic reviews**

| **R-AMSTAR Criteria** | **Bailey 2009** | **Bernard Bonnin 1995**^]^ | **Bhogal 2006, & Zemek 2008** | **Boyd 2009** | **Bravata 2009** | **Bussey Smith 2007** | **Chang 2010** | **Coffman 2009** | **de Jongh 2012** | **Denford 2013** | **Gibson 2002** **& Gibson 2004** | **Kirk 2012** | **Marcano Belisario 2013** | **Moullec 2012** | **Newman 2004** | **Postma 2009** | **Powell 2009** | **Press 2012** | **Ring 2007** | **Stinson 2009** | **Tapp 2010** | **Toelle 2004** | **Welsh 2011** |
| --- | --- | --- | --- | --- | --- | --- | --- | --- | --- | --- | --- | --- | --- | --- | --- | --- | --- | --- | --- | --- | --- | --- | --- |
| Was an ‘a priori’ design provided? | 4 | 4 | 4 | 4 | 4 | 3 | 4 | 3 | 4 | 3 | 4 | 2 | 4 | 3 | 4 | 4 | 4 | 3 | 3 | 2 | 4 | 4 | 4 |
| Was there duplicate study selection and data extraction? | 4 | 1 | 4 | 4 | 4 | 2 | 4 | 3 | 4 | 4 | 4 | 4 | 4 | 2 | 1 | 1 | 4 | 4 | 1 | 4 | 4 | 4 | 4 |
| Was a comprehensive literature search performed? | 4 | 4 | 4 | 4 | 3 | 4 | 4 | 2 | 4 | 4 | 4 | 3 | 4 | 4 | 3 | 2 | 4 | 4 | 4 | 4 | 4 | 4 | 4 |
| Was the status of publication used as an inclusion criterion? | 3 | 3 | 3 | 2 | 2 | 1 | 3 | 1 | 3 | 1 | 4 | 2 | 3 | 1 | 2 | 1 | 3 | 2 | 3 | 1 | 4 | 4 | 4 |
| Was a list of studies (included and excluded) provided? | 4 | 3 | 4 | 4 | 4 | 1 | 4 | 2 | 4 | 2 | 4 | 0 | 4 | 2 | 1 | 1 | 4 | 2 | 3 | 2 | 4 | 4 | 4 |
| Were the characteristics of the included studies provided? | 4 | 1 | 4 | 4 | 4 | 4 | 4 | 4 | 4 | 3 | 3 | 4 | 4 | 4 | 3 | 4 | 2 | 4 | 4 | 4 | 4 | 4 | 4 |
| Was the scientific quality of the included studies .... assessed and documented? | 4 | 4 | 4 | 4 | 4 | 1 | 4 | 4 | 3 | 4 | 4 | 4 | 4 | 1 | 2 | 2 | 4 | 3 | 4 | 4 | 4 | 4 | 4 |
| .... used appropriately in formulating conclusions? | 1 | 2 | 4 | 4 | 4 | 2 | 4 | 3 | 3 | 4 | 4 | 0 | 4 | 3 | 4 | 2 | 2 | 3 | 4 | 1 | 3 | 2 | 4 |
| Methods used to combine the findings of the studies appropriate? | 4 | 3 | 2 | 3 | 4 | 4 | 4 | 2 | 2 | 4 | 4 | 1 | 3 | 3 | 1 | 4 | 3 | 4 | 4 | 2 | 3 | 3 | 4 |
| Was the likelihood of publication bias assessed? | 1 | 1 | 4 | 3 | 4 | 1 | 3 | 3 | 1 | 4 | 1 | 1 | 2 | 2 | 1 | 1 | 1 | 2 | 2 | 1 | 2 | 2 | 2 |
| Was the conflict of interest stated? | 3 | 1 | 4 | 3 | 3 | 3 | 2 | 2 | 3 | 3 | 3 | 2 | 3 | 2 | 1 | 1 | 3 | 3 | 3 | 3 | 3 | 3 | 3 |
| **Total score/44** | **36** | **27** | **41** | **39** | **40** | **26** | **40** | **29** | **35** | **36** | **39** | **23** | **39** | **27** | **23** | **23** | **34** | **34** | **35** | **28** | **39** | **38** | **41** |

**Table S4b: Weighting of included systematic reviews (PRISMS)**

| **Review** | **Total number participants** | **Quality score** | **Weighting** |
| --- | --- | --- | --- |
| Zemek, 2008 (& Bhogal 2006) | 365 | 41 | ** |
| Bailey, 2009 | 617 | 36 | ** |
| Bernard Bonnin, 1995 | 1290 | 27 | ** |
| Boyd, 2009 | 7845 | 39 | *** |
| Bussey Smith, 2007 | 957 | 26 | * |
| Chang, 2010 | 113 | 40 | ** |
| Coffman, 2009 | 8077 | 29 | ** |
| Gibson, 2002 (& Gibson 2004) | 6090 | 39 | *** |
| Moullec, 2012 | 3006 | 27 | ** |
| Newman, 2004 | 2004 | 23 | ** |
| Postma, 2009 | 2316 | 23 | ** |
| Powell, 2009 | 2460 | 34 | *** |
| Ring, 2007 | 4588 | 35 | *** |
| Tapp, 2010 | 2157 | 39 | *** |
| Toelle, 2004 | 967 | 38 | ** |
| Welsh, 2011 | 2342 | 41 | *** |
| Bravata 2009 | 13476 | 40 | *** |
| de Jongh 2012 | 16 | 35 | ** |
| Denford 2013**^.^** | 7883 | 36 | *** |
| Kirk 2012 | 2195 | 23 | ** |
| Marcano Belisario 2013 | 408 | 39 | ** |
| Press 2012 | 1459 | 34 | *** |
| Stinson 2009 | 826 | 28 | * |

**Table S4c. Risk of bias in the PRISMS update RCTs**

|  | Random Sequence Generation (Selection Bias) | Allocation Concealment (Selection Bias) | Blinding of participants and personnel (Performance Bias) | Blinding of outcome assessment (Detection bias) | Incomplete outcome data (Attrition bias) | Selective reporting (Reporting bias) | Other bias |
| --- | --- | --- | --- | --- | --- | --- | --- |
| Al-sheyab 2012 | 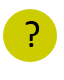 | 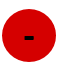 | 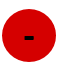 | 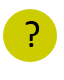 | 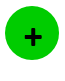 | 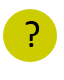 | 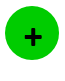 |
| Baptist 2013 | 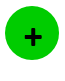 | 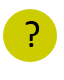 | 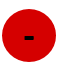 | 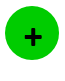 | 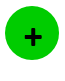 | 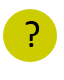 | 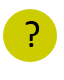 |
| Ducharme 2011^[^ | 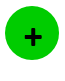 | 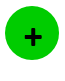 | 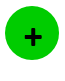 | 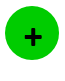 | 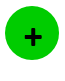 | 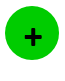 | 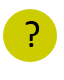 |
| Goeman 2013 | 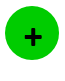 | 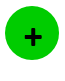 | 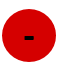 | 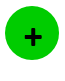 | 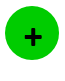 | 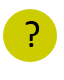 | 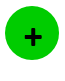 |
| Halterman 2011 | 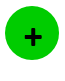 | 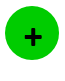 | 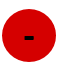 | 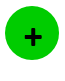 | 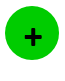 | 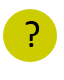 | 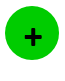 |
| Horner 2013 | 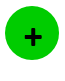 | 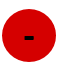 | 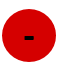 | 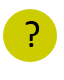 | 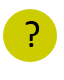 | 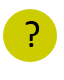 | 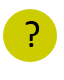 |
| Joseph 2013 | 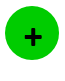 | 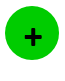 | 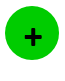 | 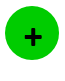 | 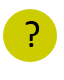 | 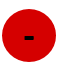 | 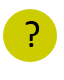 |
| Khan 2014 | 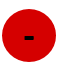 | 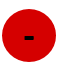 | 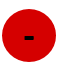 | 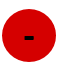 | 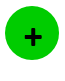 | 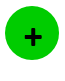 | 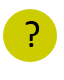 |
| Rhee 2011 | 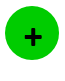 | 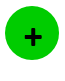 | 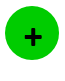 | 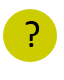 | 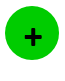 | 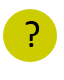 | 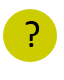 |
| Rikkers-Mutsaerts 2012 | 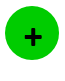 | 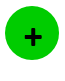 | 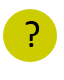 | 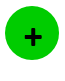 | 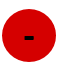 | 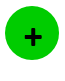 | 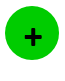 |
| Shah 2011 | 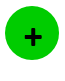 | 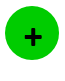 | 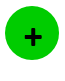 | 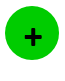 | 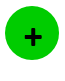 | 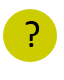 | 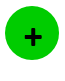 |
| van Gaalen 2014 | 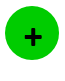 | 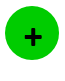 | 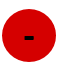 | 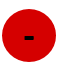 | 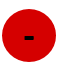 | 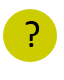 | 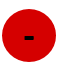 |
| Wong 2011 | 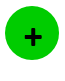 | 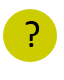 | 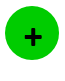 | 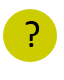 | 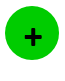 | 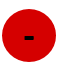 | 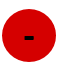 |

**Additional file 4d. Checklist of economic evaluations.**

| **Authors** | **Q1** | **Q2** | **Q3** | **Q4** | **Q5** | **Q6** | **Q7a** | | **Q7b** | **Q8a** | **Q8b** | **Q9** | **Q10a** | **Q10b** | **Q11** | **Q12** | | **Q13** | **Q14** | **Q15a** | **Q15b** | **Q16** | **Q16a** | **Q16b** | **Q16c** |
| --- | --- | --- | --- | --- | --- | --- | --- | --- | --- | --- | --- | --- | --- | --- | --- | --- | --- | --- | --- | --- | --- | --- | --- | --- | --- |
| Gallefoss et al 2001 | √ | √ | 1 | 5 | 9 | √ | √ | | √ | √ | √ | √ | Χ | NA | NA | √ | | √ | Χ | Χ | NA | √ | 12 | √ | √ |
| Kaupinnen et al 1998 | √ | √ | 2 | 5 | 9 | √ | √ | | √ | √ | √ | Χ | Χ | NA | NA | √ | | √ | Χ | Χ | NA | √ | 12 | √ | √ |
| Schermer et al 2002 | √ | √ | 1 | 5 | 11 | √ | √ | | √ | √ | √ | √ | Χ | NA | Χ | √ | | √ | Χ | Χ | NA | √ | 12 | √ | √ |
| Van der Meer et al 2011 | √ | √ | 1 | 5 | 11 | √ | √ | | √ | √ | √ | √ | Χ | NA | NA | √ | | √ | √ | X | NA | √ | 12 | x | √ |
| *Q1 Study clarity* | | | | | | | | *Q5 Economic study design* | | | | | | | | | *Q11 Discounting* | | | | | | | | |
| *Q2 Comprehensive description of competing alternatives* | | | | | | | | 9 = Cost-effectiveness Analysis (CEA) (32%) | | | | | | | | | *Q12 Incremental analysis* | | | | | | | | |
| *Q3 Perspective* | | | | | | | | 10 = Cost Consequence Analysis (CCA) (63%) | | | | | | | | | *Q13 Allowance for uncertainty* | | | | | | | | |
| 1 = Societal (26%) | | | | | | | | 11 = Cost Utility Analysis (CUA) (5%) | | | | | | | | | *Q14 Missing data handled appropriately* | | | | | | | | |
| 2 = Health care system & patient (8%) | | | | | | | | *Q6 Design adequacy given study type* | | | | | | | | | *Q15a Economic model* | | | | | | | | |
| 3 = Health care system (55%) | | | | | | | | *Q7a Relevant costs identified* | | | | | | | | | *Q15b Appropriateness of economic model* | | | | | | | | |
| 4 = Not clear (11%) | | | | | | | | *Q7b Relevant consequences identified* | | | | | | | | | *Q16 Funder stated(Y/N)*  *Q16a Type of funder*  12 = Public/voluntary sector (70%)  13 = Private sector (16%)  14 = Do not state (14%) | | | | | | | | |
| *Q4 Study design* | | | | | | | | *Q8a Costs measured accurately* | | | | | | | | |  |  |  |  |  |  |  |  |  |
| 5 = Randomised Control Trial (RCT) (55%) | | | | | | | | *Q8b Consequences measured adequately* | | | | | | | | |  |  |  |  |  |  |  |  |  |
| 6 = Case Control Trial (CCT) (13%) | | | | | | | | *Q9 Statistical analysis appropriateness given the design* | | | | | | | | |  |  |  |  |  |  |  |  |  |
| 7 = Before and after (24%) | | | | | | | | *Q10a Sub-group analysis* | | | | | | | | | *Q16b Generalisability* | | | | | | | | |
| 8 = Decision model (8%) | | | | | | | | *Q10b Sub-groups pre-specified* | | | | | | | | | *Q16c Presentation and discussion of key results* | | | | | | | | |

Χ, no; √, yes; NA, not applicable.
