# Supplementary material for: Systematic meta-review of supported self-management for asthma: a healthcare perspective
Source: BMC Med. 2017 Mar 17;15:64. doi: 10.1186/s12916-017-0823-7 (PMC5356253; doi:10.1186/s12916-017-0823-7)
Supplement: Additional file 5: — Characteristics of included studies and key outcomes. (DOCX 169 kb) [file 12916_2017_823_MOESM5_ESM.docx]

**Additional file 5. Included studies: characteristics and outcomes**

**Table S5a: Table of findings of PRISMS systematic reviews and their relevance to the meta-review questions.**

| **Reference & weighting.**  n RCTs,  R-AMSTAR;  n participants,  Date range | **Comparison** | Relevance to meta-review questions | | | | **Interventions included** | **Target group(s)** | **Synthesis** | **Main results** | **SR authors’ conclusions**  ***Meta-review authors’ comments*** |
| --- | --- | --- | --- | --- | --- | --- | --- | --- | --- | --- |
|  |  | What is the impact ? | Specific target groups ? | Which components? Components | Contextual facilitators ? |  |  |  |  |  |
| **Bailey,**  **2009** **  4 RCTs,  R-AMSTAR 36; 617 participants,  2000-2008 | Culturally-orientated programmes vs.  Usual care or limited / generic education. | ✓ | Ethnic minority groups |  |  | Education, action plans, triggers and avoidance, development of collaboration with healthcare services. Delivered by language-appropriate asthma educators. | Minority groups: Puerto Rican; African-American; Hispanic; Indian sub-continent. Adults and children. | Meta-analysis      Narrative analysis | Reduced hospitalisation in children [RR: 0.32 (95%CI 0.15 to 0.70) 1RCT] but not reported in adults  Improved QoL in adults [WMD 0.25 (95%CI 0.09 to 0.41)] [2 RCTs]  2 of 2 RCTs reported a reduction in A&E visits, and hospitalisations: one reported no difference in ‘use of healthcare resources’  2 of 3 reported improved QoL (adults) | Authors’ conclusion: The limited evidence suggests that culturally-orientated education programmes for adults and children from minority groups are effective in reducing use of healthcare resources, and improving QoL.  *Comment: This review is limited by a small number of studies and small sample sizes in two of the studies.*  4 RCTs: 617 participants (range 24-344)  Publication bias not reported |
| **Bernard Bonnin,**  **1995** **  11 RCTs,  R-AMSTAR 27; 1,290 participants,  1981-1991 | Interactive teaching on self-management  vs.  standard care. | ✓ | [Children] |  |  | Interactive teaching (one-to-one or group) to support asthma self-management. | Children: aged 1 to 18 yrs. Overall severity classified as ‘mild to moderate’ | Meta-analysis  Narrative analysis | Reduced hospitalisation [ES: 0.06 (+/- -.08)] and emergency visits [ES 0.14 (+/-0.09)] [5 RCTs]  It was among children with high baseline numbers of hospitalizations and emergency visits that the greatest subsequent reduction in morbidity was observed. | Authors’ conclusion: Modest impact of self-management teaching programs on paediatric asthma morbidity. Inclusion of pre-school children and overall mild/moderate asthma severity will have reduced impact.  *Comment: Limited reporting of data limits interpretation of this review.*  11 RCTs: 1290 participants (range not reported)  Publication bias not reported |

| **Bhogal,**  **2006** **  4 RCTs  R-AMSTAR 41; 355 participants,  1990-2004 | Symptom-based written PAAPs  vs.  Peak flow-based PAAP |  | [Children] | Sm vs peak flow monitoring |  | Asthma education plus PAAPs formatted to target both parents and children. Generally contained 3 steps: often employing ‘traffic lights’.  Monitoring varied: either daily or when symptomatic.[Most trials required completion of daily diary] | Children aged 6 -19 yrs with mild to severe asthma. | Meta-analysis | Symptom-based PAAPs reduced unscheduled care compared to peak-flow-based PAAPs [RR 0.73 (95%CI 0.55 to 0.99)] [4 RCTs]  There was no difference in hospital admissions [RR 1.51 (95%CI 0.35 to 6.65)]  Peak flow-based PAAPs reduced the number of symptomatic days/week [mean difference: 0.45 days/week (95%CI 0.04 to 0.26)] [2 RCTs]  No significant difference for adult or child QoL | Authors’ conclusion: In children, symptom-based PAAPs are superior to peak flow-based PAAPs for preventing unscheduled use of healthcare. Other outcomes were similar.  *Comment: The small number of trials identified in this review limited the power of detecting significant difference in secondary outcomes, and subgroup and sensitivity analyses.*  4 RCTs: 355 participants (range 46-168)  Publication bias ‘not enough trials’ |
| --- | --- | --- | --- | --- | --- | --- | --- | --- | --- | --- |
| **Zemek,**  **2008** **  5 RCTs  R-AMSTAR 41; 423 participants  1990 – 2005  [This SR updates Bhogal 2006] | Written PAAPs vs  No PAAP  Symptom-based written PAAPs  vs.  Peak flow-based PAAP | ✓ | [Children] | [PAAP] |  | Asthma education plus PAAPs formatted to target both parents and children. Generally contained 3 steps: often employing ‘traffic lights’.  Monitoring varied: either daily or when symptomatic. | School-aged children with mild to severe asthma | Meta-analysis | A peak flow-based PAAP reduced unscheduled care compared to no plan: [WMD -0.50 (95%CI -0.83 to -0.17)][1 RCT]  A peak flow–based PAAP compared to no plan reduced symptom scores: [WMD -11.80 (95%CI -18.22 to -5.38)] and mean difference of school days missed [WMD -1.03 (95%CI -1.85 to -0.21)] [1 RCT] | Authors’ conclusion: Written PAAPs significantly reduce acute care visits per child as compared with control subjects.    *Comment: This review updates Bhogal 2006, but includes no further evidence comparing peak flow with symptom-based PAAPs, and only one RCT comparing a PAAP with no plan.*  5 RCTs: 423 participants (range 46-168)  Publication bias: ‘no evidence’ |
| **Boyd,**  **2009** ***  38 RCTs,  R-AMSTAR 39; 7,843 participants  1985 - 2007 | Educational intervention targeted children and/or parents  vs.  Low intensity/ basic/routine education. | ✓ | [A&E] [Children] |  |  | Education + self-monitoring; therapy review; written PAAP; trigger avoidance. Settings (home, community, hospital) and professionals (nurses, educators, social workers, case managers) . Mode (including computer games) | Children (0 - 18 yrs of age) who had attended the emergency room for asthma within the previous 12 months. | Meta-analysis  Sub-group analyses | Education reduced A&E attendances: [RR 0.73 (95%CI 0.65 to 0.81) 17 RCTs], hospital admissions [RR 0.79 (95%CI 0.69 to 0.92) 18 RCTs] and unscheduled doctor visits [RR 0.68 (95%CI 0.57 to 0.81) 7 RCTs]  No effect on QoL: [WMD 0.13 (95%CI 0.73 to 0.99) 2 RCTs].  Type/timing of intervention, timing of outcome assessment, age of participants) had no effect on outcomes. | Authors’ conclusion: Asthma education aimed at children and their carers who present to the emergency department for acute exacerbations can result in lower risk of future emergency department presentation and hospital admission. The most effective type, duration and intensity of educational packages remains unclear.  *Comment:* *Large review, with significant heterogeneity between the interventions and outcomes*  38 RCTs: 7843 participants (range 16-1033)  Publication bias: ‘funnel plot: no asymmetry’ |
| **Bussey Smith,**  **2009** *  9 RCTs,  R-AMSTAR 26; 957 participants,  1986 - 2005 | Interactive computerised educational programmes  vs.  Routine care/ traditional self-management education and monitoring. | ✓ |  | IT-based interventions |  | Interactive computerised educational asthma programmes including games tailored to specific features of asthma in the individual; web-based education; personal interactive communication devices | Patients aged 3 - 75 yrs. 7 RCTs focused on children, 2 on adults. 4 RCTs evaluated urban or inner-city populations | Narrative analysis | 7 studies reported unscheduled care: 1 of 4 improved hospitalisation, and 1 of 5 reduced unscheduled care.  5 of 9 studies found statistical improvements in asthma symptoms compared to control. | Authors’ conclusion: Interactive computerised programmes appear to improve patient asthma symptoms, but there is less evidence to support the effect of these interventions on use of healthcare resources.  *Comment: Substantial diversity in the interventions. Old’ study in a fast moving field.*  9 RCTs: 957 participants (range 10-228)  Publication bias: not reported |
| **Chang,**  **2010** **  1 RCT,  R-AMSTAR 40; 113 participants  2010 | Involvement of indigenous healthcare workers (IHWs) in education programmes  vs.  Education with no IHW | ✓ | Ethnic groups |  |  | Initial clinical consultation, education reinforced by home visits from a trained IHW. Personalised, child-friendly, culturally-appropriate asthma education materials were provided. | African-American and Hispanic communities. Children aged 1-17 yrs. Mean age approx. 7 yrs. | Narrative analysis | There was no effect on hospitalisations: [OR 1.58 (95%CI 0.37 to 6.79)] or A&E attendances: [OR 0.30 (95%CI -0.17 to 0.77)] [1 RCT]  Days absent from school were reduced by 21% in the intervention group (95%CI 5-36%) [1 RCT]  Carer asthma-QoL was not significantly different: [0.25 (95%CI -0.39 to 0.89] | Authors’ conclusion: The involvement of IHW in asthma programmes targeted for their own ethnic group in one small trial was beneficial in improving most, but not all asthma outcomes in children with asthma  *Comment: This review is considerably limited by the very small sample size and the inclusion of only a single study.*  1 RCTs: 113 participants  Publication bias: not relevant |
| Coffman,  2009 **  18 asthma RCTs (of 25 studies),  R-AMSTAR 29; 8,077 participants,  1987-2007 | School-based asthma education  vs.  Usual care | ✓ | School-children |  |  | Mostly group education on asthma, medication, monitoring and avoiding triggers. Delivered by nurses; health educators; peer counsellors; teachers; computer programmes; and physicians. | Children aged 4 - 17. Only reported in some studies, severity ranged from mild to severe, and a majority of participants were black or Latino. 15 RCTs incorporated components for parents, school personnel and classmates without asthma. | Narrative analysis | Unscheduled healthcare was not reported.  School absences significantly reduced in 5 of 13 RCTs.  QoL improved in 4/6 RCTs.  Days with symptoms were reduced in 3 of 8 RCTs. Nights with symptoms improved in 1 of 4 RCTs: 1 found improvement in control group. | Asthma education was associated with improvements in self-efficacy and self-management behaviours. Asthma education was also associated with a statistically significant increase in quality of life.  *Some of the cluster RCTs may have overstated the impact of the interventions as 7 studies failed to use appropriate statistical methods.*  18 RCTs: 8077 participants (range 20-3443)  Publication bias: ‘symmetrical for school absence’ |

| **Gibson,**  **2002** ***  36 RCTs,  R-AMSTAR 39; 6,090 participants  1986 – 2001 | Self-management programmes  vs.  usual care | ✓ |  | Regular review | ✓ | Education (100%); self-monitoring of symptoms and/or peak expiratory flow (92%); regular review of treatment and asthma severity by a medical practitioner (67%); written action plan (50%).  Sub-group analyses based on these service models | Adults and children. Recruited from a variety of settings including: hospital; emergency room; OPD; community setting; general practice. | Meta-analysis  Sub-group analysis | Self-management education reduced hospitalisations [RR 0.64 (96%CI 0.50 to 0.82) 12 RCTs], A&E visits [RR 0.82 (95% CI 0.73 to 0.94) 13 RCTs], unscheduled consultations [RR 0.68 (95% CI 0.56 to 0.81) 7 RCTs]  Self-management education improved QoL [SMD 0.29 (95% CI 0.11 to 0.47) 6 RCTs], days off work or school [RR 0.79 (95% CI 0.67 to 0.93) 7 RCTs]  Optimal self-management (supported by a PAAP and regular review) reduced hospitalisations [RR 0.58 (96%CI 0.43 to 0.77) 9 RCTs], A&E visits [RR 0.78 (95% CI 0.67 to 0.91) 9 RCTs] | Authors’ conclusion: Self-management educational programmes delivered to adults with asthma results in clinically important improvements in asthma health outcomes including reduced healthcare utilisation. These benefits are most pronounced with interventions which involve a written action plan, self-monitoring and regular medical review.  *Comment: large, high quality review*  36 RCTs: 6090 participants (range 36-689)  Publication bias: not reported |
| --- | --- | --- | --- | --- | --- | --- | --- | --- | --- | --- |
| **Gibson,**  **2004** ***  26 RCTs  R-AMSTAR 39; 6,090 participants  1987 - 2002 | Comparison of different components of written PAAPs  vs.  Usual care |  |  | Components of PAAPs |  | Components of written PAAPs: Action plans were classified as being complete if they specified when and how to increase treatment (n = 17), and as incomplete if they omitted advice on increasing ICS (n = 4) or non-specific (n = 5) if they only provided general instructions | Adults and children. Recruited from a variety of settings including: hospital; emergency room; OPD; community setting; general practice. | Action points  %predicted vs %best  Treatment advice  Non-specific plans | Benefits were found for any number of action points (2 to 4).  Both reduced hospitalisations, but only % personal best reduced A&E visits.  PAAPs which included advice on increasing ICS and starting oral steroids reduced hospitalisations, and A&E visits.  Efficacy of incomplete and non-specific action plans was inconclusive. | Authors’ conclusion: The findings of this review strongly support the use of individualised complete written PAAPs. Effective action plans can be based on symptoms or PEF and use 2, 3 or 4 action points. PEF based plans should use personal best PEF and not % predicted PEF for the action point. Treatment instruction should include both inhaled and oral steroids.  *Comments: In some cases there were insufficient studies to allow a comparison.*  26 RCTs: not reported  Publication bias: not reported |

| **Moullec,**  **2012** **  18 RCTs,  R-AMSTAR 27; 3,006 participants  1990 - 2010 | Interventions for improving inhaled corticosteroid adherence  vs.  Usual care |  |  |  | ✓ | All studies included self-management (education; behaviour therapy; motivational interviewing). Some included components of the Chronic Care Model (CCM): decision support; delivery system design; clinical information systems. | Moderate - severe asthma patients (one also included COPD patients). Age range 35 - 50 yrs. Women over-represented in most studies | Meta-analysis | Effect size for adherence to ICS compared by number of components of the CCM in the study:  1 CCM components (n=13): small ES 0.29 (95%CI 0.16 to 0.42)  2 CCM components (n=5): large ES 0.53 (95%CI 0.40 to 0.66)  3 CCM components (no studies)  4 CCM components (n=4) very large ES 0.83 (95%CI 0.69 to 0.98) | Authors’ conclusions: This review concludes that the more CCM components included within interventions, the greater the effects on ICS adherence outcomes.  *Comments:*  *A relatively small number of adequately powered studies were included, particularly for sub-group analysis on interventions with 4 CCM components.*  18 RCTs: 3006 participants (range 13-204)  Publication bias: ‘not assessed’ |
| --- | --- | --- | --- | --- | --- | --- | --- | --- | --- | --- |
| **Newman,**  **2004** **  18 asthma RCTs (of 63 studies)  R-AMSTAR 23; 2,004 participants,  1997 – 2002 | Self-management interventions  vs.  standard care/basic information, | ✓ |  |  |  | Face-to-face individual or group interventions, led by professionals, and focussed on symptom monitoring trigger avoidance and adherence to medication. A few used techniques to address barriers to effective self-management. | Adults with 3 LTCs (including asthma) | Narrative analysis  and comparison between interventions | 7 of 11 studies reported a reduction in unscheduled health care.  6 of 12 studies reported improved QoL  Eight of the studies for asthma measured severity of symptoms and frequency or the percentage of symptom-free days. The three studies that recorded reductions in severity all used education and action plans.  8 of 14 reported improved adherence  Little difference recorded between symptoms or PEF guided actions | Authors conclusions: Evidence suggests importance of action plans in combination with education for reduced healthcare utilisation. However, no clear patterns can be established as to the optimal self-management provision.  *Comments: Limited date range (1997 - 2002) reduced the evidence-base. Also not all clinical outcomes were included in this review.*  18 RCTs: 2004 participants (range 27-245)  Publication bias: not reported |
| **Postma,**  **2009** **  7 RCTs,  R-AMSTAR 23; 2,316 participants  2004 - 2008 | Environmental interventions for children delivered by community health workers (CHWs)  vs.  Usual care | ✓ | Ethnic groups, [Children] |  |  | Home visits by CHWs from the same community as participants.  Education on asthma triggers/avoidance, resources to reduce allergen exposure, information about asthma and medication use, and lifestyle advice. | Participants aged 5-9 years, low-income, and ethnic minorities (mainly African American and Hispanic). 28% -75% of participants tested positive to at least 1 allergen. | Narrative review | 3 of 6 studies reported reduced hospitalisation, and reduced unscheduled consultations.  4 of 6 reported reduced A&E attendances  ‘Consistent and significant decrease in caregiver-reported asthma symptoms among intervention subjects compared with control subjects in 6 studies’ | Authors conclusions: Overall, the studies consistently identified positive outcomes associated with CHW-delivered interventions, including decreased asthma symptoms, day- time activity limitations, and urgent care.  *Comment****:*** *Difficult to interpret because there are two interventions: allergen avoidance (generally considered to be ineffectiveand education delivered by CHWs focussed on self-management/allergen avoidance.*  7 RCTs: 2316 participants (range 100-937)  Publication bias: not reported |

| **Powell,**  **2009** ***  15 RCTs,  R-AMSTAR 34; 2,460 participants  1990 - 2001 | Self-management of medication  vs.  Physician-reviewed management  Comparison of modified PAAPs with optimal self-management |  |  |  , regular review | ✓ | Self- vs physician adjustment of medication (n=6 studies)  Peak flow vs symptoms PAAPs (n=6)  Other variations (n=3) | Adults with asthma recruited from a range of primary, community, A&E, secondary care | Self- vs physician treatment  Symptoms vs peak flow  Modified PAAPs | Of 6 studies, 4 reported no difference in hospitalisation, 1 reported no difference in A&E visits, 3 reported inconsistent effects on unscheduled consultations.  Of 6 studies, 6 reported no difference in hospitalisation, 5 reported inconsistent effects on A&E visits  Omission of regular review increased unscheduled consultations.[1 RCT]  Reduction of intensity of education increased unscheduled consultations. [1 RCT]  Verbal (as opposed to written) PAAPs had no effect on hospitalisations, or A&E visits. [1 RCT] | Authors’ conclusions: Self-adjustment of treatment with the aid of a written PAAP is as effective as regular medical review in preventing unscheduled care .  Peak flow based PAAPs are equivalent to symptom-bases PAAPs.  These findings are clinically important as they enable interventions to be tailored to patient preference, patient characteristics and the resources available.  *Comments: Limited evidence for the 3 modifications of self-management*  15 RCTs: 2460 participants (range 40-569)  Publication bias: not reported |
| --- | --- | --- | --- | --- | --- | --- | --- | --- | --- | --- |
| **Ring,**  **2007** ***  14 RCTs,  R-AMSTAR 35; 4,588 participants  1993 - 2005 | Interventions encouraging use of PAAPs  vs.  usual care |  |  |  | ✓ | Interventions promoting action plan ownership and/or use. Interventions were diverse and could be categorised as educational; reinforcing or prompting; asthma clinics; asthma management systems; or quality improvement.  Most PAAPs were delivered by professionals. | Adults or children with asthma with moderate-to-severe asthma or were recruited following medical care for acute asthma  . | Narrative analysis | The only outcome was ownership and use of PAAPs. 4 of 5 studies of self-management education reported an increase in PAAP ownership, 1 reported an increase in PAAP use.  1 of 2 studies of telephone consultations reported an increase in PAAP ownership, and increased understanding of PAAPs.  1 of 2 studies of asthma clinics reported increased PAAP ownership.  1 of 2 studies of asthma management systems reported more children receiving PAAPs, and the other reported significantly greater use. Educating HCPs facilitated PAAP use for up to 2 years post intervention and a quality improvement project had no effect. | Primary care professionals could encourage the ownership and use of action plans through the implementation of proactive practice-based organisational systems    *Comments: This review reports process (PAAP ownership) and not clinical outcomes. Many potentially eligible studies did not report ownership of PAAPs (despite PAAPs being a component of the intervention) so had to be excluded*  14 RCTs: 4588 participants (range 76-637)  Publication bias: not reported |

| **Tapp,**  **2010** ***  13 RCTs,  R-AMSTAR 39; 2,157 participants  1979 - 2009 | Asthma education during/after A&E visit  vs.  Usual care | ✓ | Post A&E attendance |  |  | Education provided by asthma or A&E nurses to adults during, or within a week of attending the A&E for acute asthma, included written PAAPs; monitoring; triggers; use of medication and inhalers; importance of follow up. | Adults recruited during an A&E attendance. | Meta-analysis  Narrative analysis | The intervention reduced hospital admissions [RR 0.50 (95%CI 0.27 to 0.91) 5 RCTs], A&E visits [RR 0.66 (95% CI 0.41 to 1.07) 8 RCTs]  Effect on QoL (2 RCTs ) was inconsistent. There was no effect on days off work/school. | Authors’ conclusions: Whilst the evidence is supportive of educational interventions to reduce readmission following an episode of acute asthma in adults, the review does not provide evidence to suggest that other important markers of long-term asthma morbidity are affected.  *Comment: High quality review, heterogenous outcomes prevented meta-analysis for many of the secondary outcomes*  13 RCTs: 2157 participants (range 20-289)  Publication bias: not reported |
| --- | --- | --- | --- | --- | --- | --- | --- | --- | --- | --- |
| **Toelle,**  **2004** **  7 RCTs,  R-AMSTAR 38; 967 participants  1990 - 2001 | Individualised written PAAP  vs.  No plan,  *or* Symptom based PAAP  vs.  Peak flow based PAAP |  |  | Written PAAP |  | Peak flow based written PAAP or symptom-based written PAAP delivered in primary or tertiary care. | Adults (and children in 1 RCT) with asthma. Mean age of adults in the RCTs ranged from 28 - 45 yrs | Meta-analysis  Sub-group analysis | Unscheduled use of healthcare was only assessed in one RCT and not reported by the SR.  There was no difference between symptom and peak flow based PAAPs in hospitalisations [RR 1.17 (95%CI 0.31 to 4.43) 3 RCTs] or A&E attendances [RR 1.17 (95% CI 0.31 to 4.43) 3 RCTs].  Symptom-based PAAPs reduced unscheduled consultations [RR 1.34 (95% CI 1.01 to 1.77) 2 RCTs] | Authors conclusions: It is not possible to conclude whether use of written PAAPs alone (as opposed to part of a comprehensive self-management programme) leads to an improvement in asthma management behaviours.  *Comments: Small number of included studies and the small number of patients recruited in the studies have limited the ability to draw conclusive findings*  7 RCTs: 967 participants (range 72-250)  Publication bias: ‘no evidence’ |
| **Welsh,**  **2011*****  12 RCTs,  R-AMSTAR 41; 2,342 participants,  1986 - 2010 | Home-based self-management education  vs.  Routine care or general asthma education | ✓ | Ethnic [Children/teens] |  |  | Language appropriate asthma education (asthma, triggers, medication, inhalers, and self-management supported by written PAAPs. Additional strategies included printed materials, homework, IT electronic devices, scheduled phone calls and access to a 24-hour hotline. | Children (mostly up to 12 yrs rather than teenagers with mild – severe asthma, with recent healthcare visit. Range of demographic settings, though many were ethnic and/or deprived communities in US. | Meta-analysis  Narrative analysis | Heterogeneity of outcomes precluded meta-analysis of hospitalisation and A&E visits. There was no difference between groups in mean number of unscheduled consultations [mean difference 0.04 (95%CI -0.20 to 0.27) 2RCTs)]  2 of 5 studies reporting hospitalisation one found a reduction and one an increase in the intervention group.  Effect on A&E visits (6 RCTs), unscheduled consultations (3 RCTs) was inconsistent.  Overall no effect on QOL (5 RCTs). | Authors’ conclusion: Limited, inconsistent evidence was found for home-based interventions  Comment: Review findings are limited by heterogeneity of outcomes limiting the pooling of data.  12 RCTs: 2342 participants (range 15-473)  Publication bias: ‘insufficient trials’ |

| **Bravata 2009** ***  63 RCTs (of 79 studies)  R-AMSTAR 40; 13,476 participants.  1966-2006 | Self-management Quality Improvement (QI)strategies  vs  QI strategies other than self-management | ✓ | [Children] |  |  | Self-monitoring or self-management. Patient / Caregiver education. Provider Education. Organisational change and Interventions with multiple QI strategies. | Children (<18 years). | Meta-analysis | Interventions targeting parents/caregivers reduced hospitalisation rates by 1.2% per year (95% CI, 0.1 to 2.4%) n=5  Self-management intervention studies improved symptom-free days by 2.8% (95% CI 0.6% to 5%) = 0.8 days per month n=7; and reduced monthly school absenteeism by 0.4% (95% CI, 0%-0.7%) = 0.1 day per month n=16.  Longer duration of intervention increased the effect on school absences. | Authors conclusions: The QI strategies with the greatest body of evidence of effectiveness include the self-management and patient education interventions.  Comment: *Includes 3 types of study design: RCTs, controlled before-after trials and interrupted time series trials.*  63 RCTs:13,476 participants (range 14-1023)  Publication bias: ‘no substantial bias’ for most outcomes. Possible bias for absenteeism and hospital days |
| --- | --- | --- | --- | --- | --- | --- | --- | --- | --- | --- |
| **Denford, 2013** ***  38 RCTs,  R-AMSTAR 36; 7883 participants 1993-2000 | Interventions targeting asthma self care  vs  usual care/less intensive intervention. | ✓ |  | Behaviour change techniques |  | Behavioural change techniques including: self-monitoring (n=30), instruction (n=27), goal setting (n=26), and inhaler technique (n=24). Less commonly: breathing control; tailored information; cognitive behavioural therapy, active learning techniques, communication skills; addressing concerns, efficacy building, developing illness models | Adults (18 and over) with a diagnosis of asthma. | Meta-analysis  Meta-regression (to explore association between specific techniques and change in asthma outcomes) | Intervention group participants had reduced asthma symptoms [SMD= - 0.38, (95% CI -0.52 to 0.24) 27 RCTs] and unscheduled healthcare use [OR 0.71 (95% CI 0.56 to 0.9) 23 RCTs].  Increased adherence to preventative medication. For intervention groups compared to control [OR 2.55 (95% CI: 2.11 to 3.10)16 RCTs]  Interventions including stress management were less effective in reducing symptoms [SMD=0.01 (95% CI:-0/08, 0.1)] vs [SMD=-0.44 (95% CI -0.57, 0.31)] ‘Active involvement of participants’ associated with reduced unscheduled health care use [OR=0.50, (95% CI 0.28, 0.90) 6RCTs] vs [OR=0.79 (95% CI: 0.62, 1.01)17 RCTs]. | Authors Conclusion: Interventions targeting asthma self-care are effective. Active involvement of participants is associated with increased intervention effectiveness, but the use of stress management techniques may be counterproductive.  Comment: *Studies included describe wide ranging behaviour change techniques. Self monitoring was the most commonly reported (30 out of 38 studies)*  38 RCTs: 7883 participants (range 22-808)  Publication bias: ‘Egger test indicated significant asymmetry for symptoms and unscheduled care’ |

| **de Jongh 2012**, **  1 asthma RCT (of 4 studies)  R-AMSTAR 35; 16 asthma participants (of 182 participants).  1993 – 2009. | Mobile phone messaging applications to facilitate self- management  vs  Usual care |  |  | Mobile phone messaging |  | Self-management interventions delivered by mobile phone messaging | Included participants regardless of age, gender or ethnicity.  Did not restrict to asthma, included any long term illness. | Narrative synthesis. | In the single asthma study, there were fewer admissions (2 vs 7) but more unscheduled consultations (21 vs 15) in the intervention group compared to the usual care group.  The pooled asthma symptom score showed a significant difference between groups, favouring the intervention group (MD-0.36, 95%CI -0.56 to -0.17).  The intervention group showed no improvements in spirometry, but there was a significant reduction in peak flow variability compared to control (MD -11.12, 95% CI -19.56 to -2.68) | Author’s conclusion: the extremely small sample size of the [asthma] study means that the quality of the evidence for any of these measures is considered low.  Comment: *Extremely small sample size in asthma paper which limits the usefulness of the results.*  1 RCTs: 16 participants  Publication bias: ‘insufficient numbers’ |
| --- | --- | --- | --- | --- | --- | --- | --- | --- | --- | --- |
| **Kirk 2012** **  10 RCTs,  R-AMSTAR 23; 2,195 participants.  1995-2010. | Self-care support  Vs  Usual care | ✓ | [Children] |  |  | Any intervention aiming to help children take control of and manage their condition, promote their capacity for self-care and / or improve their health. | Children aged 18 or younger, diagnosed with a long term condition: asthma (10 RCTs), Cystic fibrosis (2), diabetes (1). | Narrative Synthesis | Significant reduction was reported in asthma admissions (2/8 RCTs), A&E attendance (5/8 RCTs) and unscheduled consultations (3/8 RCTs).  Control improved in 5 of 8 RCTs.  Qol improved in 2/5 RCTs | Authors comment: There is strong evidence of the effectiveness of interventions that target children/young people; use e-health or group-based methods; that are delivered in community settings. There is no evidence that interventions that focus on parents alone or delivered in hospital settings are effective.  Comment: *Heterogeneous interventions (3 RCTs on cystic fibrosis/diabetes)*  10 RCTs: 2195 participants (range 47-961)  Publication bias: ‘not reported’ |
| **Marcano-Belisario 2013,** **  2 RCTs  R-AMSTAR 39; 408 participants,  2000-2013. | Smart phone and tablet apps to facilitate self-management  vs  Traditional methods of supporting self-management. |  |  | Smartphone Apps. |  | Self-management support interventions provided by smartphone app. | Adults with clinician diagnosed asthma | Narrative Synthesis. | Compared to control, 2 out of 2 RCTs showed no difference in hospital admissions. 1 of 2 studies showed fewer A&E attendances, 1 RCT found no difference in unscheduled GP consultations, or out of hours consultations, but reduced primary care nurse consultations.  1 study found no difference in the mean difference in ACQ scores between the intervention and control group at 6 months. 1 of 2 studies found improved QOL in the intervention group. | Authors comment: The current evidence base is not sufficient to advise clinical practitioners, policy-makers and the general public with regards to the use of smartphone and tablet computer apps for the delivery of asthma self-management programmes. Future research should attempt to minimise the differential clinical management of patients between control and intervention groups.  Comment: *Rigorous methodology employed, however limited number of RCTs means limited conclusions possible.*  2 RCTs: 408 participants (range 120-288)  Publication bias: ‘not reported’ |
| **Press 2012** ***  7 RCTs (of15 studies),  R-AMSTAR 34; 1,459 participants.  1950-2010 | Educational interventions targeted at ethnic minority groups  vs  Usual care | ✓ | Ethnic Minority Groups |  |  | Interventions targeting ethnic minority populations in the US. 15 were education-based; a further 9 were system-level interventions.  5 were culturally tailored and community-based. The remaining 10 were hospital based. | Adults (18 or older). Ethnic minority groups.  5 studies entirely focused on African Americans (4), Asian Americans (1)  Majority of participants: 10 studies African Americans, 4 studies Latinos. | Narrative Synthesis | An education intervention reduced A&E attendance in 2 of 4 RCTs and hospital admissions in 2 of 3 RCTs  Symptoms were not reduced in any of the 3 RCTs that measured control. QoL was improved in 3 of 4 RCTs that used an asthma-related QoL outcome.. | Authors conclusion: Conclusions on the effectiveness of cultural tailoring on improving patient self-care or symptoms cannot be made as few culturally tailored studies evaluated symptoms and self-management.  System redesign showed promise, particularly the use of team-based specialty clinics and long-term follow-up after acute care visits.  Comment: *Includes US studies only. Study type varied – 10 RCTs, 6 Cohort, 2 Case Control, 6 Pre/Post*.  7 RCTs: 1459 participants (range 17-537)  Publication bias: ‘searched abstract – but not reported’ |
| **Stinson 2009**,*  4 RCTs (of 9 studies).  R-AMSTAR 28; 826 asthma participants.  1993-2008. | Internet based self-management intervention vs usual care |  | [Children] | Internet based |  | Any internet based or enabled self-management intervention | Children (6-12) or adolescents (13-18) with LTCs: asthma (4 RCTs), recurrent pain / headache (1), encopresis (1), traumatic brain injury (1), obesity (1). | Narrative synthesis. | 1 RCTs reported no difference in hospitalisations compared to control; 1 RCT reported significant reductions in A&E visits; and 1 of 2 RCTs showed fewer unscheduled consultations  4 out of 4 reported significant improvement in a measure of control.  1 of 4 asthma RCTs reported a significant benefit on QOL | Authors comment: There are the beginnings of an evidence base that self-management interventions delivered via the internet improve selected outcomes in certain childhood diseases.  Comment: *Limited by a lack of clear aims, outcomes and heterogeneity. Now 7 years old in a rapidly advancing field.*  4 RCTs: 826 participants (range 24-438)  Publication bias: ‘not reported’ |

Abbreviations: ES: effect size, FU: follow up, ICS inhaled corticosteroid, LTC: long-term conditions, OR: odds ratio, PAAP: Personalised Asthma Action Plan, PEF: peak expiratory flow, RR: risk ratio, QoL: quality of life, SMD, standardised mean difference, WMD, weighted mean difference, 95%CI: 95% confidence interval. d: day, m: week, m: month, y: year

**Table S5b. Table of findings of RCTs and their relevance to the meta-review questions.**

| **Reference & weighting.** participants, date range | **Comparison** | Relevance to meta-review questions | | | | **Study type and Interventions included** | **Target group(s)** | **Bias** | **Main results** | **RCT authors’ conclusions**  ***Meta-review authors’ comments*** |
| --- | --- | --- | --- | --- | --- | --- | --- | --- | --- | --- |
|  |  | What is the impact ? | Specific target groups ? | Which components? Components | Contextual facilitators ? |  |  |  |  |  |
| **Al-Sheyab 2012**  n=261  HIGH risk of bias | Adolescent Asthma Action program  (Triple A)  vs  Standard care  No defined 1^o^ outcome |  | Adolescents | Peer led education |  | Cluster RCT.  Triple A (Described by Gibson 1998). Peer leaders from year 11 were trained to deliver program to years 8, 9 and 10. | Adolescents in northern Jordanian high school. The Intervention group had fewer females, less symptomatic and with higher English proficiency. | Researcher not blinded. Unclear if children were recruited before/after allocation. | FU at 3m.  Compared to control improvements QoL improved [I: 5.42 (0.14) vs C: 4.07 (0.14) mean diff 1.35 1.04–1.76] | Author’s conclusion: This trial demonstrated that the Adolescent Asthma Action program can be readily adapted to suit different cultures and contexts. Adolescents in Jordan were successful in teaching their peers about asthma self-management and motivating them to avoid smoking.  *Comment: Follow up at 3 months only. Borderline significant difference in baseline QoL score. Lack of significance in results however improvements identified.* |
| **Baptist 2013** n=70  HIGH risk of bias | Personalised asthma self-regulation intervention  vs  Education session  1^o^ outcome: QoL (AQLQ) |  | Older adults | Health educator, self-regulation group |  | RCT.  Six-session program (group/ telephone). Patients selected an asthma-specific goal, identified problems, and addressed potential barriers.  Control had a single session of basic education | Aged 65 or older. Physician diagnosis of asthma, no restriction in severity. Majority Caucasian. | Researcher blind.  No baseline data. Incomplete outcome data and possible selective outcome reporting. | No between-group differences in ED visits or hospitalisations. After adjusting for baseline differences, unscheduled visits and overall healthcare utilization was significantly lower at 6, but not 12m  Asthma control (ACQ) was similar at 6 and 12 months. At 12m intervention group participants were 4.2 times more likely to have an ACQ score <0.75.  [1^o^] Mean QoL was significantly higher in the intervention group than in control at (1,6 and 12 months), | Authors comment: A self-regulation intervention can improve asthma control, quality of life, and healthcare utilization in older adults.  *Comment: No pre-intervention measures so impossible to account for possible baseline differences.* *No mention of composite healthcare utilisation index in the analysis.* |
| **Ducharme 2011**  N=219  LOW risk of bias | ‘Take-home plan’ post A&E visit with written PAAP + prescription information  vs  Prescription but no PAAP or information  1^o^ outcome: adherence to ICS at 28d |  | [Children] A&E attendees | PAAP with prescription information |  | RCT.  Intervention is written PAAP with a ‘formatted’ prescription for ICS (i.e. including information about use) issued by A&E doctor on discharge following asthma exacerbation. | Canadian children aged 1-17 recruited during A&E attendance for acute asthma (78% were under the age of 6y) | Possible contamination between groups if more than one attendance to ED. | At 28 days, there was no between group differences in unscheduled care.  Compared to control, at 28 days children given the PAAP had better asthma control. [Proportion with Asthma Quiz Score <2: I: 58% vs C: 41% RR 1.36 (1.04, 1.86)]  At 28 days, there was no between-group difference in child/caregiver QoL.  [1^o^] At 28 days both groups showed marked decline in patient adherence to ICS from 90% at Day 1 to about 50% at Day 14, with no significant group difference.  More intervention group children filled their oral steroid prescription [I: 64%. C: 53%. RR 1.31 (95%CI 1.07, 1.60)] | Author’s comments: Provision of a written PAAP significantly increased patient adherence to inhaled and oral corticosteroids and asthma control and physicians’ recommendation for maintenance fluticasone and medical follow-up, supporting its independent value in the acute-care setting.  *Comment: Predominantly pre-school children. Unscheduled care and asthma control were secondary outcomes. Possible contamination between groups if more than one attendance to ED* |
| **Goeman 2013.**  n=114  Low risk of bias | Person centred education including device technique  vs  Written information only.  1^o^ outcomes: ACQ and adherence |  | Older adults | Personalised education |  | RCT.  Personally tailored education session with asthma educator based on responses to a questionnaire.  Control is written leaflet (standard). | 55 year or older, community based asthmatics with no restriction in asthma severity. | Concealment is ensured, but not blinding of outcome assessment | [1^o^] Compared to control group, at 12 months the intervention group had better asthma control. [ACQ. Mean diff 0.3 (95% CI: 0.06–0.5); p = 0.01] and better asthma-related QoL (p = 0.01).  There was no significant difference in number of steroid courses (p=0.17)  At 12 months a significantly greater number of intervention group (n= 36, 61%) owned a PAAP compared to control (n= 21, 37.5%) p = 0.015  [1^o^] Adherence to preventer medication was similar at 12m (p = 0.015) | Authors’ comments: Asthma outcomes in older people can be significantly improved by delivering tailored education that identifies specific patient concerns and unmet needs.  *Comment: Well conducted study. Concern re blinding of outcome assessment. Baseline differences in* |

| **Khan 2014**  N=91  HIGH risk of bias | Asthma education + individualised written PAAP  vs  Asthma education (excluding PAAP)  1^o^ outcome: A&E attendances |  | Ethnic (Trinidad) | Written PAAP |  | RCT.  Both groups receive standardised individual asthma education during an OP clinic visit from a paediatrician. Intervention group receive and trained in using a WAAP  Both groups had monthly phone or face-to-face FU. | 1-14 years.  Partly controlled asthma (defined as daytime symptoms >2x/week, any activity limitation or nocturnal symptoms, lung function < 80%best or an exacerbations in previous year.), Recruited children via A&E or paediatric clinic | Randomisation by randomly selected day of clinic attendance (so no concealment).  Researcher blinded. | [1^o^] There was a trend to improved outcomes at 6m but no significant between group difference in proportion of children attending A&E I:36 vs. C:52% (p=0.141)  There was no between group difference in unscheduled doctor visits, asthma attacks, missed school days, night-time awakenings. | Authors comment: The results of this study suggest that the provision of personalized WAAPs may play a useful role in the management of children with partly controlled asthma but is no better than standard care.  *Comment: Small and short (6m) study. Treating paediatrician aware of allocation (selection bias). WAAP is different from UK – may have been unhelpful as instructions for red zone are seek help immediately! Under-powered, and results do not reach significance,* |
| --- | --- | --- | --- | --- | --- | --- | --- | --- | --- | --- |
| **Halterman 2014**  N=638  LOW risk of bias | Personalised prompts for clinicians and parents, practice training and feedback  vs Written guidelines  1^o^ outcome: symptom-free days at 2m |  | [Children], Urban deprived communities | Implementation support/monitoring | Community based | Cluster RCT.  PAIR-UP intervention practices received personalised clinician and parent prompts + blank PAAP; practice training; biannual feedback  Control practices sent guidelines. | Urban, primary care practices in deprived communities.  Parents/children aged 2-12yrs with persistent poorly controlled asthma  Recruited from waiting room over 4yr study | Recall bias: parents asked to remember their child’s symptom free days of the past fortnight | 11% of children in both groups had an A&E visits or hospitalisation.  [1^o^] Compared to control practices at 2m, children in the PAIR-UP practices had more symptom free days [I: 10.2 (SD4.8) vs C: 9.5 (5.1) d/2w (mean diff, 0.78; 95% CI, 0.29 to 1.27) but the difference was not significant at 6m.  Nights (but not days) with symptoms remained significant at 6m [I: 1.4 (3.0) vs C: 1.8 (3.2) mean diff: −0.43 (−0.77 to −0.09)] | Authors comment: The PAIR-UP intervention improved the delivery of preventive asthma care and reduced asthma morbidity at 2 months, but was no longer significant at 6 months.  *Comment: Large study. Intervention focussed at the clinician which is effective. Intervention was initiated in 2009 with feedback and training yearly. Presumably the intervention practices should get better / more effective over time. Data are not presented from different years.* |
| **Horner 2014**  N=183  UNCLEAR risk of bias | Asthma plan for kids  vs  Teaching on general health and well-being.  No defined 1^o^ outcome |  | [Children] Rural population |  |  | Cluster RCT  7-steps delivered in 16 sequential sessions of 15-min duration, 3d a week for 5.5 weeks, provided by school nurses during the children’s lunch break + home visit | Grades 2-5 (Age 7-11). Physician diagnosis of asthma | Participants not blinded. High risk of attrition (not all data available) and reporting bias. | There was no significant between group difference for admissions or ED visits.  There was no significant between group difference in QOL scores  Inhaler skill improved significantly in the intervention group compared to control after 4 months. Treatment group parents reported higher self-efficacy and home management scores than their control group counterparts | Authors comment: Providing an educational intervention in elementary schools to small groups of children during their lunch breaks allowed sufficient time for children to review information, complete problem solving exercises, and to practice using an inhaler and a peak flow meter. The four home visits for data collection may have contributed to increasing their knowledge of asthma.  *Comment: Study aimed to answer 3 hypothesis, and had no clear primary outcome. All outcome measures were questionnaire based at 4 home visits (except ED visits/ hospitalisations which were based on parent recall)* |

| **Joseph 2013**  N= 422  Unclear risk of bias | Web based asthma management intervention  vs  Control.  1^o^ outcome: symptom days |  | Adolescents, Urban deprived ethnic group | Web based targeted behavioural change |  | RCT.  Puff City a 4 module internet based management program targeted at African Americans / Urban adolescents with specific traits (rebelliousness; resistance to change; low motivation; low perceived emotional support) | 9 – 12 grade (14-18 years). Physician diagnosis of asthma and report >4 days of restricted activity in the past 30 days at baseline | Attrition and reporting bias | There was no difference in self-reported ED visits and hospitalisations at 12 months.  [1^o^] Compared to controls, at 12m the intervention group had fewer days with symptoms [I:3.9 (5.9) vs C: 5.2 (6.4) RR 0.8 (0.6 to 1.0)]  There was no difference in other measures of symptom control (nights with symptoms; schooldays missed; days of restricted activity; days had to change plans  Targeted sub-groups students characterised as with rebelliousness or low perceived emotional support reported fewer symptom- days. | Authors comment: A theory-based, tailored approach, with a referral coordinator, can improve asthma management in urban teens. Puff City represents a viable strategy for disseminating an effective intervention to high-risk and hard-to-reach populations.  *Comment: Outcomes and how they were evaluated poorly described. Some missing data (e.g. no baseline or 6 month readings reported; no baseline demographics). In general the results highlighted are those that favour the treatment group.* |
| --- | --- | --- | --- | --- | --- | --- | --- | --- | --- | --- |
| **Rhee 2011**  N=112  Unclear risk of bias | Peer led asthma education provided by peers at a day-camp.  vs  adult-led camp  1^o^ outcome QoL (PAQLQ] |  | Adolescents. | Peer leaders |  | RCT  Intervention is basic asthma education, psychosocial issues, asthma self-management skills taught at a day camp by peer leaders + monthly peer telephone contact  Control: Similar education delivered by adults. No phone FU. | 13-17 years. Mild/moderate/severe asthma. Asthma diagnosis for 1 year. Able to understand spoken and written English.  Recruitment varied: Keen to gain adolescents from low income families, but not a main thrust of inclusion. | Participants were blinded. Unclear if investigators were blinded but primary outcome measured by on-line/paper questionnaires. | [1^o^] Both groups reported significantly increased quality of life over time (F=4.31, P=.002), with the intervention group having significantly higher quality of life at 6m (difference, 11.38; 95% CI, 0.96-21.79; P=.03) and 9m (12.97; 3.46-22.48; P=.008).  Both groups reported improved attitude to asthma (F=11.94,P=.001); with greater improvement in the intervention group at 6m (mean difference, 4.11; 95% CI, 0.65-7.56; P=0.02).  Adolescents of male gender or low family income, reported more positive attitudes at 3,6 and 9 months | Authors comment: This study provides empirical evidence that an asthma self-management program assisted by peer leaders is a viable and developmentally appropriate option for adolescents with asthma as an alternative to paternalistic models of asthma programs for adolescents. The peer-led asthma program yielded greater improvements in positive attitudes and quality of life compared with the adult-led program.  *Comment: Useful in bringing an intervention to adolescents and particularly identifying socioeconomic groups and race. However main focus is on evaluating their intervention. Not clear if the sub group analysis in lower income and gender groups was a-priori* |

| **Rikkers Mutsaerts 2012**  N= 90  Unclear risk of bias | Internet based self -management  vs  Usual care.  1^o^ outcome: asthma-related QoL at 3 and 12m |  | Adolescents. | Internet based |  | RCT:  Internet based self-management comprised weekly control monitoring with treatment advice by a web-based algorithm  Programme had 4 domains: education (web-based + group), self-monitoring (FEV1 + ACQ), electronic PAAP and 3-6monthly medical review. | 12 – 18 years mild to severe persistent asthma on regular ICS medication and poorly controlled at recruitment | Blinding of participants and investigator unclear | There was no between-group difference in exacerbations, physicians’ visits or telephone contacts.  [1^o^] Compared to control, asthma-related QoL was better in the intervention group at 3m [PAQLQ I: 6.00 vs C: 5.68 diff: 0.40 (0.17 to 0.62)] but no different at 12m [I: 5.93 vs C: 6.05 diff: 0.05 (0.50 to 0.41)  Compared to control, asthma control was improved in the intervention group at 3m [ACQ I: 0.96 vs C: 1.19 diff: -0.32 (-0.56 to -0.08)] but no different at 12m [I: 0.83 vs C: 0.79 diff: -0.05 (-0.35 to 0.25)  There was no between-group difference in symptom-free days. | Authors comment: internet-based self-management improved asthma-related quality of life and asthma control in adolescents with not well controlled asthma after 3 months, but not after 12 months.  *Comment: Drop outs from the intervention group had poorer asthma control than the drop outs from the control. Numbers of those taking up the educational sessions (part of the IBSM intervention) was small 27 and 10 out of 46.* |
| --- | --- | --- | --- | --- | --- | --- | --- | --- | --- | --- |
| **Shah 2011**  N=150 GP s and 201 children  LOW risk of bias | GP training (PACE)  vs  No training  1^o^ outcome ownership of a PAAP |  | Children | GP training |  | Cluster RCT  GPs participated in 2x 3-hour workshops on communication and education strategies to facilitate quality asthma care | 150 GPs and 221 children with asthma in their care |  | There was no between-group difference in hospitalization or ED visits [I: 18% vs 12% diff 6% (− 4% to 15%)]  There was no between-group difference in school absence or parent absenteeism for child’s asthma  [1^o^] Compared to controls, more patients of GPs in the intervention group reported receipt of a PAAP [I: 61% vs 46% diff 15% (95% CI, 2% to 28%) | Author’s comments: The PACE Australia program improved GPs’ asthma management practices and led to improvements in some important patient outcomes.  *Comments: well-designed cluster RCT with careful attention to reducing bias (concealment/randomisation blinding of participants as well as researchers).* |

| **van Gaalen**  **2013**  N=107  HIGH risk of bias | Internet based Self -management vs  Control.  Analysis of participants 18 months after end of SMASHING trial. |  | Adults. | Internet Based |  | RCT (FU study)  Intervention consisted of education a PAAP, self-monitoring, and regular review  The 200 patients in the original trial were invited to complete follow-up questionnaires and undertake FEV1. No further intervention after the website terminated at the end of the trial. | Adults with asthma aged (18-50 years), with using ICS  107/200 (54%) participated: I: 47/101 (47%) C: 60/99 (61%).  The patients who participated at 30m were comparable to non-participants based on the ACQ scores at 0 and 12 months. but between group difference in AQLQ was greater in participants. | Performance bias (researcher not blinded to groups) and attrition bias (minimal data published). | Compared to control, at 12 months after baseline, participants in the control group had improved asthma-related QoL [AQLQ 0.37 (95% CI 0.14 to 0.61) and asthma control [ACQ -0.57 (95% CI -0.88 to -0.26).  At 30 months after baseline, there was a slightly attenuated improvement for both QoL [AQLQ adjusted between-group difference 0.29 [95% CI 0.01-0.57]) and ACQ (adjusted difference of -0.33 [95% CI -0.61 to -0.05]) scores in favour of the Intervention.  No differences were demonstrated for lung function measured as FEV1. | Authors Comment: Provision of internet-based self-management support for 1 year leads to sustained benefits in terms of asthma control and asthma-related quality of life as compared with usual care, even up to 1.5 years after terminating support..  *Comment: The authors providing the difference between two groups and graphical illustrations but no actual values. 5$% participation rate, greater in control group, with some differences in ACQ response at 12m* |
| --- | --- | --- | --- | --- | --- | --- | --- | --- | --- | --- |
| **Wong 2012**  N=80  High risk of bias | Symptom based written PAAP  vs  verbal counselling  No defined 1^o^ outcome |  | [Children] Ethnic | Written PAAP |  | Single blinded RCT. Intervention is symptom based WAAP given out at initial contact. Outcomes measured at baseline, 3, 6 and 9 months. | Malaysian children (mix of Malay, Chinese, and Indian) all severities of asthma. Ages 6-17 years. Recruitment process not described. | High risk of attrition bias and recall bias. | At 6m there was no difference in A&E/unscheduled care [I: 4 (10.8) vs C: 6 (21.1) p=0.35]  At 6m there was no difference in proportion controlled [ACT≥20 I: 81% vs C: 87% p=0.50] or with no exacerbations [ACT≥20 I: 89% vs C: 82% p=0.62] or in QoL [mean PAQLQ I: 6.11 (0.88) vs 6.11 (1.09) p=0.99] | There was no significant reduction in asthmatic exacerbations, improvement in asthma control or quality of life with the written PAAP when used in Malaysian children with all severities of asthma.  *Comment: Very scant description. No sample size calculation, but numbers are small. Some baseline differences.* |

Abbreviations: ACQ: asthma congtrol questionnaire, ACT: asthma control test, C: control, FEV_1_: forced expiratory volume in one second, FU: follow up, I: intervention, ICS inhaled corticosteroid, mAQLQ: mini Asthma-related Quality of Life Questionnaire, PAAP: Personalised Asthma Action Plan, PAQLQ: paediatric asthma-related quality of life, RR: risk ratio, QoL: quality of life, SD: standard deviation, 95%CI: 95% confidence interval. d: day, m: week, m: month, y: year

**Table S5c. Table of findings of RCTs in the RECURSIVE health economic analysis.**

| **Reference.**  **Country,**  Allocation concealment | **Study type and Interventions included** | **Comparison & follow-up assessment** | **Target group(s)** | **Health economic results** | | |  | **Formal health economic evaluation** |
| --- | --- | --- | --- | --- | --- | --- | --- | --- |
|  |  |  |  | **Quality of life** | **Healthcare utilisation (Hospitalisation)** | **Total costs** | **Unscheduled care** |  |
| **Baptist 2013**  **US**  n=70  Concealment not adequate | RCT.  Personalised asthma self-regulation intervention including six-sessions. | Usual care.  12 months. | Older adults with asthma (aged 65 or older).  Mean age 74yrs.  14% male. | Quality of life (Asthma Control Questionnaire score <0.75), was significantly higher in the intervention group than in control group (intervention: M=13, SD=41.9; control: M=5, SD=15.6). | The intervention group had fewer hospitalisations (intervention: n=0; control n=4, p=0.04). | n/a | No significant differences in A&E visits (intervention: n=1 control: n=2 p=0.58); The intervention group had fewer unscheduled care visits (intervention n= 6; control: n=14; p=0.048). | n/a |
| **Castro 2003**  **US**  n=96  Concealment not adequate | RCT.  Education, psychosocial support, self-management plan and co-ordination of care for ‘high-risk’ inpatients with asthma. | Usual private primary care.  12 months. | Adults with asthma.  Mean age 38yrs.  15% male. | No significant differences between the intervention and the control group in AQLQ overall score (intervention: M=4, SD=1.3; control: M=2.1, SD=0.9). | The intervention group had fewer hospital readmissions for asthma (intervention M=0.4, SD= 0.9; control: M=0.9, SD=1.5; control: n=14, p=0.04). | The intervention group had lower total healthcare costs Mean costs/patient I: $5,726 (SD $5,679) vs C: $12,188 (SD $19,352) mean difference $6,462, (p=0.03). | No significant differences in A&E visits (intervention: M=1.9 SD=4.3 control: M=1.4 SD=1.5 p=0.52); | n/a |
| **Clark 2007**  **US**  n=808  Concealment not adequate | RCT.  Individualised, nurse delivered, telephone counselling based on self-regulation theory. | Usual care.  12 months. | Adult women with asthma.  Mean age 49yrs.  All women. | No significant differences between the intervention and the control group in AQLQ overall score (intervention: M=2.1, SD=0.9; control: M=2.3, SD=6.2). | No significant differences on hospitalisations (intervention: M=0.2 SD=0.7; control: M=0.1 , SD=0.5). | n/a | The intervention group had greater reductions in unscheduled care visits and A &E visits (intervention: M change=-0.63, SD=2.4; control: M change=-0.24 SD=1.5) | n/a |
| **De** **Oliveira 1999**  **Brazil**  n=52  Concealment not adequate | RCT.  Outpatient asthma education programme, including a treatment plan. | Usual care.  6 months. | Adults with moderate to severe asthma.  Mean age 38yrs.  15% male. | No significant differences between the intervention and the control group in QoL overall score (combined AQLQ and morbidity score) (intervention: M=28, SD=17; control: M=50, SD=15; P=0.0005). | No significant differences on hospitalisations (intervention: M=0; control: M=0.5 , SD=0.8; p=0.08). | n/a | The intervention group had fewer A&E visits (intervention: M=0.7, SD=1.0; control: M=2 SD=2). | n/a |
| **Gallefoss 2001**  **Norway**  n=78  Concealment not adequate | RCT.  Group-based and individual education and counselling programme, including the provision of a written self-management plan. | Usual primary care.  12 months. | Adults with asthma.  Mean age 44yrs.  21% male. | Greater improvements were found in the intervention compared to control group in SGRQ overall score (intervention: M=20, SD=15; control: M=36.5, SD=18; p<0.001). | n/a | The intervention had higher marginally higher direct costs than the control group (p=0.054) but total costs including both direct and indirect costs were the same in both treatment groups (p=0.510). |  | **Cost effectiveness analysis** (Societal and health service perspective)  Incremental SGRQ gain 16.3; Health costs difference 1900; All cost diff -5500. |
| **Gruffydd-Jones 2005**  **UK**  n=174  Concealment not adequate | RCT.  Targeted routine asthma care by nurse-led, telephone delivered, using the Royal College of Physicians three questions, to formulate individualised written asthma action plan. | Usual primary care.  12 months. | Adults with asthma.  Mean age 50yrs.  40% male. | No significant differences between the intervention and the control group in ACQ (intervention: change from baseline -0.11, 95%CI -032 to 0.11; control change from baseline -0.18 95%CI -0.38 to 0.02; p=0.349). | n/a | Total costs were lower in the intervention group but did not reach statistical significance (Mean difference £122.35; p=0.071). | n/a | n/a |
| **Honkoop 2014**  **Netherlands**  n=611  Adequate concealment | RCT.  2 intervention groups:  Nurse-led care to symptom-based control  [Nurse-led care to FeNO control] | Care to usual standard.  12 months. | Adults with asthma. Mean age 40yrs.  28% male. | No significant differences between the intervention groups and the control group in EQ5D (QALYs) I: 0.91 (0.90 to 0.91) vs C: 0.89 (0.88 to 0.90), mean diff 0.01 (-0.02 to 0.04) (p>0.05) | n/a | No significant differences between the intervention groups and the control on total costs I: $4591 (4123 to 5060) vs C: $4180 (3818 to 4543). Mean diff $411 (-904 to 1797) (p>0.05). | n/a | n/a |
| **Kauppinen 1999**  **Finland**  n=167  Concealment not adequate | RCT.  Intensive education programme, including use of inhaled drugs, peak expiratory flow, monitoring and including self-management plan for newly diagnosed patients with asthma. | Conventional education.  36 months. | Adults with asthma.  Mean age 43yrs.  44% male. | No significant differences between the intervention and the control group in EQ5D (intervention: M=0.93, CI=0.90 to 0.94; control: M=0.91, CI=0.8.9 to 0.94; p=0.47). | n/a | The intervention had higher total direct and total costs than the control group I: £345 (247 to 1758) vs C: £294 (0 to 8078) (p<0.001). (p<0.001). Indirect costs were higher in the control group but the difference did not reach statistical significance (0.067) | **n/a** | **Cost effectiveness analysis** (Societal and health service perspective)  Intensive education associated incremental gain of 0.02 15D  Incremental diff in health costs of £51. |
| **Krieger 2015**  **US**  n=366  Adequate concealment | RCT. | Usual care.  12 months. | Adults with asthma.  Mean age 41yrs.  27% male. | Greater improvements were found in the intervention compared to control group  miniAQLQ at 12m I: 4.64 vs C: 4.17. Mean change in miniAQLQ. I: 0.95 vs C: 0.36. diff 0.50 (0.28 to 0.71), p <0.001 | No between group differences in number of urgent care episodes I: 2.10 vs C: 2.08. The number of urgent health care use episodes in the 12 months decreased significantly and similarly in both groups. Mean change I: -1.50 (-2.03 to -0.97) vs C: -1.60 (-2.13 to -1.08) diff 0.09 (-0.59 to 0.73) p=0.78 | n/a | n/a | n/a |
| **Lahdensuo 1996**  **Finland**  n=122  Concealment not adequate | RCT.  Guided self-management group, including personal education, physiotherapeutic counselling and diary recordings for patients with asthma. | Traditional treatment.  12 months. | Adults with asthma. Mean age 43yrs.  48% male. | Greater improvements were found in the intervention compared to control group in SGRQ symptom domain (intervention: M=16.6, SD=15.9; control: M=8.4, SD=18.4). | n/a | n/a | The intervention group had fewer unscheduled care visits (intervention: M=0.5 control: M=1; p=0.04). If the one year risk of traditionally treated patients is defined as 1 then for the patients in the self management group the relative risk for unscheduled outpatient visits was 0.53 (95% CI 0.29 to 0.96), | n/a |
| **Levy 2000**  **UK**  n=211  Concealment not adequate | RCT.  Structured education sessions by emergency room-based specialist nurses, using self-management plan, for emergency room attendance for asthma. | Usual primary care.  6 months. | Adults with asthma. Mean age 40yrs.  43% male. | No significant differences between the intervention and the control group in SGRQ (Intervention: M= 30.25; Control: M=28.73). mean diff 1.52 (-4.05 to 7.09). | No significant differences on hospital consultations (Median (IQR) I: 0 (1 to 3 vs C: 0 (1 to 6) Mean difference -1.3372 p=0.17). | n/a | No significant differences on emergency GP consultations Median (IQR) I: 0 (1 to 7 vs C: 0 (1 to 7) Mean difference -1.5375 P=0.14. | n/a |
| **Mancuso 2011**  **US**  n=296  Concealment not adequate | RCT.  Multicomponent, behavioural-based, emergency department education programme (workbook, behavioural contract, telephone calls, physiological feedback) for patients with asthma. | Instruction/ PF training.  12 months. | Adults with asthma. Mean age 43yrs.  23% male. | No significant differences between the intervention and the control group in AQLQ at 16w I: 5.40 (SD 1.08) vs C: 5.49 (SD 1.10) mean diff -0.09 (-0.35 to 0.17) Mean change in score I: 1.96 (SD 1.30) vs C: 1.90 (1.27) mean diff -0.06 (-0.37 to 0.25)  At 1y there was no difference in mean change I: 0.04 (95% CI -0.31 to 0.24) vs C: 0.18 (95% CI -0.08 to 0.44) difference 0.22 (95% CI _ 0.15 to 0.60). | n/a | n/a | No significant differences between the intervention and the control group in ED visits at 8 weeks Intervention: 13% (n=141), control: 11% (n=140) | n/a |
| **McLean 2003**  **Canada**  n=225  Adequate concealment | RCT.  Enhanced pharmaceutical care, including teaching of asthma self-management, medication usage and provision of asthma action plan, delivered by local community, experienced pharmacists. | Usual pharmaceutical care.  7 months. | Adults with asthma. Mean age 38yrs.  47% male. | Greater improvements were found in the intervention compared to control group in Juniper Questionnaire (intervention: M=5.13, control: M=4.45, p=0.0001). | No significant differences between the intervention and the control group in hospitalisations (Intervention: M=0.078, control: m=0.16, p=0.94). | Total costs, were lower in the intervention group compared to control group (intervention: costs per patient= $150; control: cost per patient $351). | No significant differences between the intervention and the control group in A&E visits (Intervention: M=0.04, control: m=0.21, p=0.48). | n/a |
| **Moudgil 2000**  **UK**  n=689  Concealment not adequate | RCT.  Individually-based, asthma education and optimisation of drug therapy programme, with 4-monthly review | Usual primary care.  12 months. | Adults with asthma. Mean age 35yrs.  47% male. | Greater improvements were found in the intervention compared to control group (Difference in change in AQLQ 0.22, 95%CI= 0.15 to 0.29, p<0.001). | No significant differences between the intervention and the control group in hospitalisations (OR=0.51, 95%CI=0.22 to 1.14). | n/a | No significant differences between the intervention and the control group in A&E visits (OR=0.63, 95%CI=0.23 to 1.68). | n/a |
| **Pilotto 2004**  **Australia**  N=12 practices  n=170  Concealment not adequate | Cluster RCT.  Three nurse-run asthma clinics to review the inhaler technique and encourage patients to develop action plans. | Usual medical care.  9 months. | Adults with asthma. Mean age 50yrs.  48% male. | No significant differences between the intervention and the control group in SGRQ (Mean difference= -0.5 95% CI -4.0 to 2.9). | No significant differences between the intervention and the control group in hospitalisations (intervention: n=2; control: n=0; p=0.499). | n/a | No significant differences between the intervention and the control group in A&E consultations (intervention: n=2; control: n=0; p=0.499). | n/a |
| **Pinnock 2003**  **UK**  n=278  Adequate concealment | RCT.  Nurse-delivered, routine review by telephone in primary care. | Usual primary care.  3 months. | Adults with asthma. Mean age 57yrs.  41% male. | No significant differences between the intervention and the control group in Juniper mini AQLQ (Mean difference= 0.22 95% CI -0.15 to 0.60). | None of the groups had a hospital admission for asthma (p=1.0). | n/a | None of the groups had and A&E consultation (p=1.0). | n/a |
| **Price 2004**  **UK**  n=1,553  Adequate concealment | Cluster RCT.  Use of personal action plans through implementation of adjustable dosing. | Usual care  3 months. | Adults with asthma. Mean age 48yrs.  41% male. | No significant differences in the percentage of people with improved QoL between treatment groups (intervention: 22.5%; control=23.6%). | No significant differences between the intervention and the control group in hospital admissions (intervention: n=2; control: n=2). | Expected total annual cost per patient was lower in the intervention group compared to control (total per patient daily cost was £0.17 lower in the intervention; 95% CI: 0.11 to 0.23). | Intervention group had fewer A&E visits compared to control group (intervention: n=5; control: n=11). | n/a |
| **Ryan 2012**  **UK**  n=288  Adequate concealment | RCT.  Mobile phone supported self-monitoring, including transmission of symptoms, drug use and PF with feedback according to a plan. | Paper supported self-management  6 months. | Adults with asthma. Mean age 52yrs.  41% male. | No significant differences between the intervention and the control group in AQLQ (mean change I: −0.75 (−0.94 to −0.57) vs C: −0.65 (−0.84 to −0.46). Mean difference in change scores 0.10 (−0.16 to 0.34) | No significant differences between the intervention and the control group in hospital admissions for asthma (intervention: n=3; control: n=1). | n/a | No significant differences between the intervention and the control group in A&E for asthma (intervention: n=3; control: n=0). | n/a |
| **Schermer 2002**  **Netherlands**  n=193  Concealment not adequate | RCT.  Guided, individual, SM from primary care physicians, including  educational tools for patient and physician, and PF monitoring. | Usual primary care.  24 months. | Adults with asthma. Mean age 39yrs.  42% male. | No significant differences between the intervention and the control group in AQLQ (Mean difference: 10, 95%CI= -3 to 23). | No hospital admissions in any treatment group. | Total costs was marginally lower in the self-management group but the difference was non-significant (Total cost: I: 1,084 euros (938, 1,228) vs C: 1,097 euros (933, 1,260) mean diff -13euros). | No A&E visits in any treatment group. | Cost effectiveness analysis (Societal and health service perspective)  Incremental QALY gain 0.015. Incremental total cost €-13 ; Incremental health cost €11  Incremental health ICER €33/QALY. |
| **Shelledy 2009**  **US**  n=166  Concealment not adequate | RCT.  In-home asthma disease management programme, respiratory therapist (RT) vs nurse (N)-led, involving asthma education for patient and family, educational tools and care plan. | Usual primary care.  6 months. | Adults with asthma. Mean age 44yr.  22% male. | RT intervention had significantly higher changes scores in SGRQ compared to control group I(RT) -11.0 (SD 15.0) vs I(N) -6.0 (SD 14) vs C: -2.5 (SD 15) p<0.05. | Both intervention groups had fewer hospitalisations compared to usual care (Intervention N: M=0 (SD=0); Intervention (RN): M= 0.04 SD=0.2; Control: 0.20, SD= 0.5; p<0.05) | Both intervention groups had lower hospitalisation costs compared to usual care (I(RT): $202 (SD 960); I(N): $0 (SD 0) vs C: $1,065 (SD 3,088); p<0.05) | Intervention groups had fewer A&E visits compared to usual care but the difference was non-significant (Intervention(RT): M= 0.09 SD=0.3; intervention (N) 0.26 SD 0.8; Control: 0.37, SD= 0.20; p<0.9). | n/a |
| **Sundberg 2005 Sweden**  n=97  Concealment not adequate | RCT.  Interactive computer-based education plus nurse support. The program provided; (1) basic information about asthma; (2) information about medication use as well as inhaler technique and self-monitoring; (3) information about asthma triggers | Usual care.  12 months | Young adults with asthma. Mean age 19yrs. 55% male | No between group difference in Living with Asthma Questionnaire I: 163.6 (SD4.2) vs C: 166.2 (SD4.0) (p>0.05). | No between group difference in hospital admissions (1 admission in each group). | n/a | No between group difference in A&E visits (I: n=17; C: n=16). |  |
| **van der Meer 2011**  **Netherlands**  n=200  Concealment not adequate | RCT.  Internet-based self-management program, including electronic personal action plan, group and online education. | Internet based SM vs Usual OP care.  12 months. | Adults with asthma. Mean age 37yrs.  55% male | No significant differences between the intervention and the control group in EQ5D mean difference in EQ-5D utility was 0.006 (95% CI, -0.042 to 0.054).  QALYs (Mean difference: 0.024 95% CI, 20.016 to 0.065). | No significant differences between the intervention and the control group in hospital admissions (mean cost: I: $571 vs C: $589 Mean diff $-17 p=0.95). | No significant differences between the intervention and the control group in total healthcare costs (Mean difference=37 p=0.94). | n/a | **Cost effectiveness analysis** (Societal and health service perspective)  Incremental QALY gain 0.024; Incremental total cost $641; Incremental health cost $37  Incremental health ICER $1541/QALY. |
| **Yilmaz 2002**  **Turkey**  n=80  Concealment not adequate | RCT.  Outpatient clinic, special education programme. | Usual secondary care.  36 months. | Adults with asthma.  Mean age 29yrs.  37% male. | Greater improvements were found in the intervention compared to control group in QoL AQLQ scores: intervention: M=197.1, SD=17.8; control: M=176.7, SD=33.7; p=0.009). | No significant differences between the intervention and the control group in hospitalisations (0 in intervention; 4 hospitalisations in the control group). | n/a | Intervention group had fewer A&E visits compared to usual care significant (Intervention: n=0; Control:7; p=0.01). | n/a |
| **Yoon 1993**  **Australia**  n=76  Concealment not adequate | RCT.  Brief, group-based, single session, education programme including inhaler use, adjust medication dosage using a treatment plan. | Usual OP care.  10 months. | Adults with asthma.  Mean age not reported.  28% male. | No significant differences between the intervention and the control group in QoL (intervention: M=4.0, SD=4.38; control: M=3.96, SD=3.34; p>0.05). | Fewer participants in the intervention group had hospital admissions compared to usual care (Intervention: n=1; Control: n=7, p<0.001). | n/a | Fewer participants in the intervention group made A&E visits compared to usual care but the difference was non-significant (Intervention: n=3; Control: n=7). | n/a |

Abbreviations: A&E: accident and emergency, ACQ: asthma control questionnaire, AQLQ: asthma quality of life questionnaire, C: control, EQ5D: EuroQol five dimensions questionnaire, FeNO: fractional exhaled nitric oxide, FU: follow up, I: intervention, ICER: incremental cost-effectiveness ratio, IQR: interquartile range, n/a: not available, PAAP: Personalised Asthma Action Plan, PEF: peak expiratory flow, QALY: quality adjusted life years, QoL: quality of life, SD: standard deviation, SGRQ: St George’s Respiratory Questionnaire, 95%CI: 95% confidence interval. d: day, w: week, m: month, y: year
